# Description of mitochondrial oxygen tension and its variability in healthy volunteers

**DOI:** 10.1371/journal.pone.0300602

**Published:** 2024-06-03

**Authors:** Meryem Baysan, Mark Broere, Maarten E. Wille, Jule E. Bergsma, Egbert G. Mik, Nicole P. Juffermans, Roula Tsonaka, Johanna G. van der Bom, Sesmu M. Arbous

**Affiliations:** 1 Department of Intensive Care Medicine, Leiden University Medical Center, Leiden, the Netherlands; 2 Department of Clinical Epidemiology, Leiden University Medical Center, Leiden, the Netherlands; 3 Jon J van Rood Center for Clinical Transfusion Research, Sanquin/Leiden University Medical Center, Leiden, the Netherlands; 4 Department of Anesthesiology, Laboratory of Experimental Anesthesiology, Erasmus MC- University Medical Center Rotterdam, Rotterdam, the Netherlands; 5 Department of Intensive Care Medicine, OLVG Hospital, Amsterdam, the Netherlands; 6 Department of Laboratory of Translation Intensive Care, Erasmus MC- University Medical Center Rotterdam, Rotterdam, the Netherlands; 7 Department of Biomedical Data Sciences, Leiden University Medical Center, Leiden, the Netherlands; Albert Einstein College of Medicine, UNITED STATES

## Abstract

**Objectives:**

Describing mitochondrial oxygenation (mitoPO_2_) and its within- and between-subject variability over time after 5-aminolevulinic acid (ALA) plaster application in healthy volunteers.

**Design:**

Prospective cohort study.

**Setting:**

Measurements were performed in Leiden University Medical Center, the Netherlands.

**Participants:**

Healthy volunteers enrolled from July to September 2020.

**Interventions:**

Two ALA plasters were placed parasternal left and right, with a 3-hour time interval, to examine the influence of the calendar time on the value of mitoPO_2_. We measured mitoPO_2_ at 4, 5, 7, 10, 28, and 31 hours after ALA plaster 1 application, and at 4, 5, 7, 25, and 28 hours after ALA plaster 2 application.

**Primary and secondary outcome measures:**

At each time point, five mitoPO_2_ measurements were performed. Within-subject variability was defined as the standard deviation (SD) of the mean of five measurements per timepoint of a study participant. The between-subject variability was the SD of the mean mitoPO_2_ value of the study population per timepoint.

**Results:**

In 16 completed inclusions, median mitoPO_2_ values and within-subject variability were relatively similar over time at all time points for both plasters. An increase in overall between-subject variability was seen after 25 hours ALA plaster time (19.6 mm Hg vs 23.9 mm Hg after respectively 10 and 25 hours ALA plaster time).

**Conclusions:**

The mitoPO_2_ values and within-subject variability remained relatively stable over time in healthy volunteers. An increase in between-subject variability was seen after 25 hours ALA plaster time warranting replacement of the ALA plaster one day after its application.

**Trial registration:**

ClinicalTrials.gov with trial number NCT04626661.

## Introduction

Adequate tissue and cellular oxygenation is one of the cornerstones of therapy in critically ill patients, which is guided by regular monitoring of the patient’s circulation and oxygenation [1]. Monitoring techniques used for these purposes include mean arterial pressure, lactate, venous-to-arterial carbon dioxide difference, central venous oxygen saturation, near infrared spectroscopy (NIRS) and side stream darkfield (SDF) imaging. These techniques are limited to measurement of microcirculatory or tissue oxygenation. Furthermore, newer techniques like NIRS and SDF measurements are not used as a standard in clinical practice due to its technical limitations and troublesome interpretations [2–6]. For the assessment of cellular oxygenation there are currently no standardized bedside monitoring solutions, but several techniques are being investigated for this purpose [7, 8].

One of them is the cellular oxygen metabolism measurement monitor (COMET) measuring intracellular oxygenation at the end of the oxygen cascade. It has been developed to non-invasively measure oxygen tension in the mitochondria, which is called the mitoPO_2_, using the oxygen-dependent protoporphyrin IX (PpIX)-triplet state lifetime technique (TSLT) [9, 10]. Exogenous administration of the precursor of PpIX, 5-aminolevulinic acid (ALA), is used as a dye to optimize the measurement of oxygen tension at the mitochondrial level, and to ensure mitochondrial origin of the measurement [10]. A number of studies in animals [11, 12] and humans [13–18], have shown the COMET measurement systems’ robustness and clinical potential as a system to assess cellular oxygenation.

Monitoring of mitoPO_2_ in critically ill patients with this system, and potentially guide therapy based on the measurements, would require various measurements over a time period after the initial measurements. However, little is known about measurement repeatability of the mitoPO_2_ measurement with the COMET system [13, 15, 17, 19]. A pilot study evaluating the COMET mitoPO_2_ measurement system in critically ill patients receiving red blood cell transfusion showed an increase in mitoPO_2_ values over time, and an increase in the between- and within-subject variability in mitopO_2_ values during the first 24-hours after ALA-plaster application [20]. This increased variability in the between-subject and within-subject values in the pilot study might have been caused by the instable condition of critically ill patients or might have been caused by the decreased measurement sensitivity of the COMET system after an extended period of measurements after application of ALA-plasters. In the latter case, one would expect to see the same increase in variability in healthy volunteers as in critically ill patients over time [19–21].

The present study therefore aimed to describe the mitoPO_2_, the within- and between-subject variability of the mitoPO_2_ values during the first 31 hours after application of the ALA plaster in healthy volunteers.

## Materials and methods

### Study design

We performed a prospective cohort study in healthy volunteers at Leiden University Medical Center, the Netherlands. We used LUMC notice boards to recruit volunteers. Interested individuals gave written informed consent after assessment of eligibility by a trained study team member. Adult individuals without an active or chronic disease were eligible, while pregnant or breast-feeding women, volunteers with hypersensitivity to brown plaster, and persons with insufficient comprehensibility of the Dutch language were not. Eligible healthy volunteers were recruited from July 1st to September 11^th^ 2020 and were followed-up until October 11^th^ 2020. The healthy volunteers were instructed to refrain from doing sports, perform labored work, or shower during their participation in the study. The institutional ethics committee of Leiden-The Hague-Delft approved the study, as part of a bigger project to study between- and within-subject variability in different cohorts (reference P20.003). The study was conducted according to the declaration of Helsinki and its later amendments. The authors confirm that all ongoing and related trials for this intervention were registered in ClinicalTrials.gov (NCT04626661). We used the Transparent Reporting of Evaluations with Nonrandomized Designs (TREND) checklist when writing our report (S1 Table) [22].

### Patient and public involvement

The public and patients were not involved in the preparation and design of the study, students of technical medicine were. The results of the study were shared with the study participants.

### Data collection

Demographic, physiological and safety data of each included healthy volunteer were collected at inclusion in an electronic case report form in Castor Electronic Data Capture [23] Demographic data included age, sex, comorbidities, body mass index and smoking status (S1 File). Physiological data included heart rate, blood pressure and peripheral oxygen saturation. During the study measurements, the study team member observed and collected data regarding (serious) adverse event (AE). One week and one month after the last study measurement, data regarding (serious) adverse events were collected via a digital survey, which was send to every participant. The digital survey included the following data: protective measures at measurement locations, duration of these protective measures, observed AE after study measurements (erythema, pain, itching, burning sensation, exfoliation of the skin, blistering, crusting, other), location of AE, duration of AE in days, and severity of AE according to the study participant on a visual analogous scale from 0–10.

Study data were collected using the COMET system (Photonics Healthcare, Utrecht, the Netherlands, CE marked). Description of the PpIX-TSLT technique behind the COMET system can be found in multiple studies [9, 10, 20, 24, 25]. A valid mitoPO_2_ measurement can be performed after at least four hours after ALA plaster application [10]. The output of a mitoPO_2_ measurement with the COMET measurement device includes the mitoPO_2_ value, skin temperature and the signal quality of the measurement [10]. All data were coded to ensure anonymity of the volunteers. Access to identifiable data was restricted to the coordinating investigator (M Baysan) and was only used for follow-up of the healthy volunteers regarding adverse events and monitoring of the study by independent monitors.

### Definitions of ALA plaster time, calendar time, between- and within-subject variability

Measurement repeatability has been described as “precision under similar conditions with replicate measurements over a short period of time” [19]. Within-subject and between-subject variability were used to address the measurement repeatability of the mitoPO_2_ measurement with the COMET measurement device. Within-subject variability was defined as the standard deviation (SD) of the mean of the five measurements per timepoint of a study participant. The between-subject variability was the SD of the mean mitoPO_2_ of the study population per timepoint. Within-subject variability of the population per timepoint was calculated as the mean SD of the mean of 5 mitoPO_2_ measurements per timepoint per study participant. Between-subject variability was calculated as the SD of the mean mitoPO_2_ of the total study population per timepoint.

To study the variability of mitoPO_2_ over time, we applied two plasters at the left and right side of the sternum, 3–5 centimeters apart. We applied the second plaster, three hours after the first plaster. Our assumption hereby was that despite heterogenous oxygen distribution in the skin, mitoPO_2_ values measured at the left and right of the sternum at the same time, would be comparable. The aim was to collect mitoPO_2_ measurements on two time scales: calendar time and plaster time. Calendar time was defined as the time of the day the measurement was performed. ALA plaster time was defined as the number of hours since application of the ALA plaster. With two plasters, applied with three hours’ time difference, we assumed to be able to discern the effect of duration of application and time of the day on the mitoPO_2_.

### Study procedure

We performed the study with two ALA plasters and we pragmatically used a study duration of 31 hours based on the combination of 24-hour study duration in the aforementioned pilot study and logistics of the current study [20].

After the informed consent procedure, the healthy volunteers were given oral and written instructions, ALA plasters of 2x2cm (Alacare, Photonamic GmbH, Wedel, Germany) and alcohol patches. Participants were instructed to place the first ALA plaster parasternal at 7 A.M. on measurement day, after firmly cleaning the anterior chest wall with alcohol. Three hours later, at 10 A.M., a study team member placed a second ALA plaster at the opposite parasternal location, after cleaning it with alcohol. Thus, two ALA-plasters were in place with a 3-hour time interval, one on each side of the sternum, to be able to compare ALA plaster time (i.e. the time that has passed since the plaster was administered) with calendar time (i.e. the actual time of the measurement). The study team member marked the circumference of each ALA plaster. After placement of the participant in supine position, the first ALA plaster was temporarily removed after four hours induction of PpIX. The removed ALA patch was kept next to the participant during the measurements with the COMET measurement device. One to two minutes after removal of the ALA patch, the COMET measurement device was gently placed on the skin by a study team member, within the marked circumference of the removed ALA plaster to start the mitoPO_2_ measurements. Directly following the five mitoPO_2_ measurements at the respective timepoints, the removed ALA plaster was re-placed on the skin to protect the exposed skin against phototoxicity. This procedure was repeated for each plaster at each timepoint.

MitoPO_2_ measurements consisted of a validation phase and a measurement phase. During the validation phase, a PpIX-TSLT signal quality of ≥25% during the measurement at measurement location was needed for the mitoPO_2_ measurements to be valid [20]. The signal quality of the mitoPO_2_ measurement was measured by the COMET measurement device itself, where a higher signal quality corresponds with a more robust mitoPO_2_ measurement. The mitoPO_2_ measurement was additionally validated by occlusion of the microcirculation at measurement location, by applying local pressure on the measurement device. In doing this, an immediate drop in mitoPO_2_ level was expected, as well as a fast recovery after release of the pressure on the microcirculation [10, 20]. After this validation procedure, mitoPO_2_ was measured once per minute for five minutes, to obtain a mean mitoPO_2_, with its corresponding SD for each participant at each time point. To minimize movement artifacts, the measurement probe was stabilized at the measurement location by manually holding the cable at the end of the measurement probe. To minimize the effect of different light conditions on the PpIX utilization, the room lights were turned off during all measurements.

The timepoints for mitoPO_2_ measurements with the first ALA plaster were 4, 5, 7 10, 28, and 31 hours after ALA plaster application (ALA plaster time), while the timepoints for the second ALA plaster were at 4, 5, 7, 25, and 28 hours ALA plaster time. This corresponded with calendar time of 11 A.M. (only plaster 1), 12 P.M. (only plaster 1), 2 P.M. (plaster 1 and 2), 3 P.M. (only plaster 2), 5 P.M. (plaster 1 and 2), 12 P.M. the next day (plaster 1 and 2), and 3 P.M. the next day (plaster 1 and 2) for these measurements. The timepoints were chosen to get insight into the course of the between- and within-subject variability over time, while keeping the study logistically manageable.

After completion of all measurements, the exposed skin was protected from sunlight for an additional 24 hours with a waterproof plaster (Kliniplast border, Medeco, Oud-Beijerland, the Netherlands). The participants were given instructions to minimize sun exposure on the ALA plaster locations for at least 24 hours. A schematic overview of the study procedure and mitoPO_2_ measurement can be found in S1 and S2 Figs.

### Statistical analyses

The sample size of 17 subjects was calculated to achieve a statistical power of 90%, with a significance level of 5%, to test for differences in one-sample mean within-subject variability of 8.7 mm Hg to 4mm Hg, with a standard deviation of 5.67 mm Hg and a drop-out rate of 10%, as depicted in the study protocol. The study population characteristics, signal quality and skin temperature per timepoint and per plaster were described using mean and SD, or median and interquartile ranges (IQR), as appropriate. Categorical variables were presented as number (percentage). The mitoPO_2_, its within-subject variability and between-subject variability of each plaster at each time point were described using respectively median mitoPO_2_ (IQR), median within-subject variability (IQR), and SD of mean mitoPO_2_. Overall mitoPO_2_, and overall within-subject variability were calculated using the median value of the composed data of plaster 1 and 2 in concurrent measurement time points, while the between-subject variability was based on the SD of the mean mitoPO_2_ of the composed data of plaster 1 and 2. Concurrent measurements of plaster 1 and 2 were performed at 4,5,7 and 28 hours ALA plaster time, which corresponds with the measurements respectively at 2 P.M., 5 P.M., 12 P.M.(+1 day), and 3 P.M.(+1 day) calendar time. The mitoPO_2_ and within-subject variability were visualized using a boxplot and spaghetti plot. Between-subject variability was visualized using error bars and a spaghetti plot. Data regarding adverse events were described with number (percentage). All analyses were performed using R (R foundation for Statistical Computing, Vienna, Austria) [26].

## Results

### Characteristics of study population

**Fig 1:**
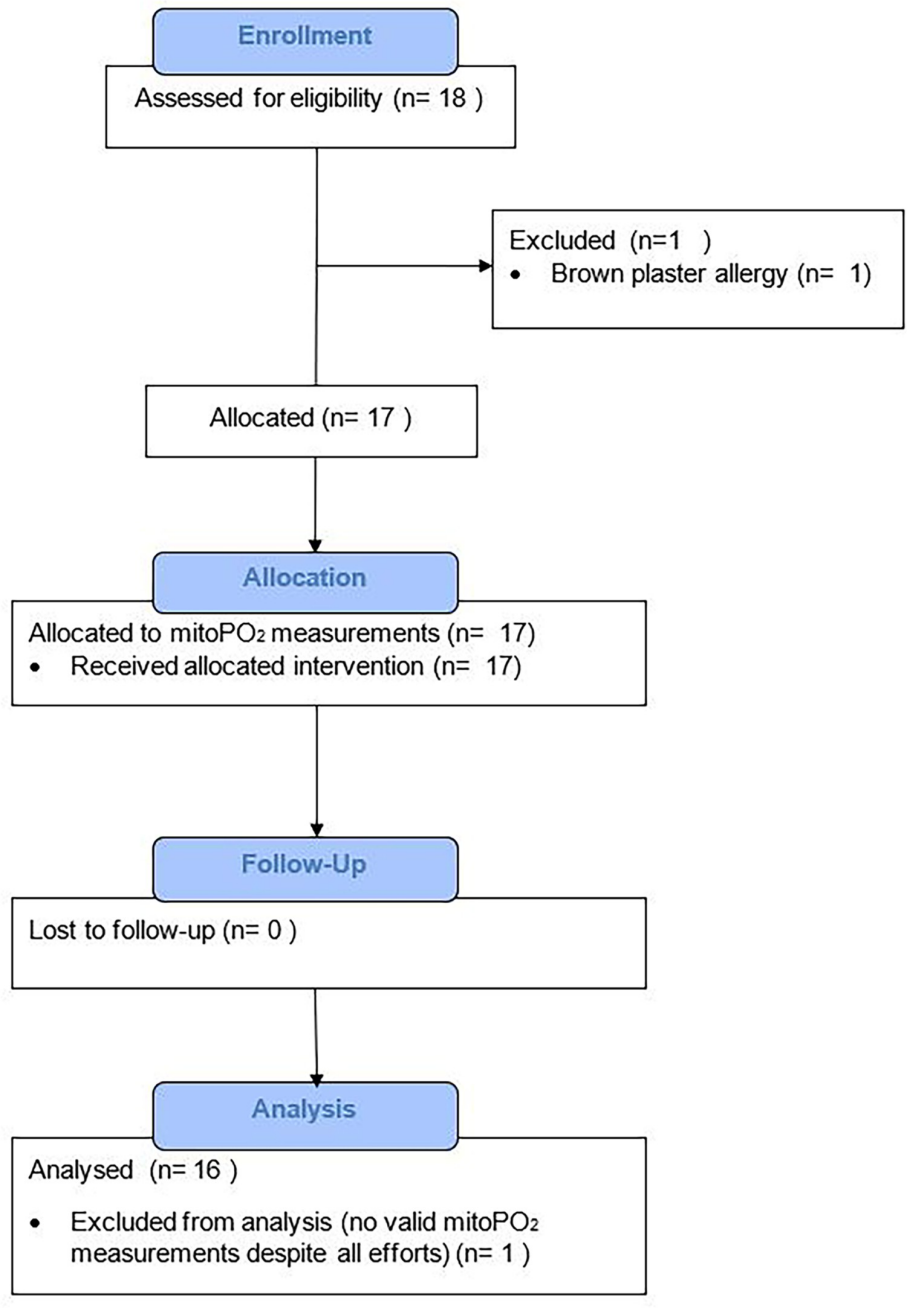
Flow chart of study cohort selection.

After assessment of eligibility, 18 healthy volunteers were included in this study, 16 were analyzed (Fig 1). One healthy volunteer was excluded due to brown plaster allergy. Another healthy volunteer was excluded from the analyses, since no valid mitoPO_2_ measurement could be achieved during the study. The signal quality of this volunteer remained below 25% after multiple efforts. Furthermore, no decrease of mitoPO_2_ could be seen after application of pressure on the measurement device at each timepoint in this volunteer. The mean age of the included 16 participants was 22.4 (SD 1.8) years, ten were female (62.5%). Further details of the study population are depicted in Table 1.

**Table 1:** Characteristics of study population.

Characteristics	Volunteers (n = 16)
Age, in year—mean (SD)	22.4 (1.8)
Male sex–n (%)	6 (37.5%)
BMI, in kg/m^2^ –median (IQR)	22.5 (21.0–25.0)
Smoker–n (%)	3 (18.8%)
*Packyears if smoker–median (IQR)*	*0*.*35 (0*.*19–0*.*35)*
SpO_2_, in %—median (IQR)	99.0 (98.8–99.0)
Heart rate, in beats per minute—median (IQR)	68.0 (54.3–92.3)
Diastolic blood pressure, in mm Hg—median (IQR)	79.5 (72.0–89.0)
Systolic blood pressure, in mm Hg—median (IQR)	120.0 (115.3–132.3)
Time between ALA plaster 1 application and start of first measurement, in min–median (IQR)	303.0 (298.0–312.5)
Time between ALA plaster 2 application and start of first measurement, in min–median (IQR)	257.0 (249.3–263.8)

*ALA* 5-aminolevulinic acid, *BMI* body mass index, *IQR* interquartile range, *SD* standard deviation, *SpO*_*2*_ peripheral measured oxygen saturation

### MitoPO_2_ measurement characteristics

Median sensor temperature was 30.4 (IQR 29.6–30.7) ⁰C in plaster 1 and 31.8 (IQR 31.7–32.2) ⁰C in plaster 2. The median sensor temperature remained between 30.0–30.7 ⁰C in plaster 1, while it was between 30.4–32.0 ⁰C in plaster 2 (S2 Table). The median signal quality was 33.2 (IQR 29.5–35.6) % in plaster 1 and 31.2 (IQR 29.0–34.4) % in plaster 2 after 4 hours after ALA plaster application. The median signal quality increased after 7 hours ALA plaster time to 41.0 (IQR 31.0–49.9) % in plaster 1 and 47.8 (IQR 44.7–62.7) % in plaster 2. The signal quality remained above the limit of 25% during all measurements (S2 Table). Furthermore, there were data missing after 4 hours ALA plaster time in plaster 1 in 4 participants (S3 Table), which was due to poor validation measurement performances in 3 participants and no measurement in 1 participant. After 5 hours ALA plaster time in plaster 1, there was 1 volunteer with remaining poor validation measurement performances. There were no missing data in measurements with plaster 2 or in later timepoints for plaster 1. The mitoPO_2_ values and within-subject variability values were not normally distributed at each time point, both at ALA plaster time and calendar time (S3 and S4 Figs).

### Course of mitoPO_2_

#### Calendar time

The median mitoPO_2_ of plaster 1 at 2 P.M. was 37.5 (IQR 28.2–66.2) mm Hg, while it was 45.9 (IQR 37.6–54.7) mm Hg in plaster 2 (Table 2). At 5 P.M. the median mitoPO_2_ of plaster 1 was 45.2 mm Hg (IQR 36.8–60.2), while it was 45.5 (IQR 41.2–50.2) mm Hg in plaster 2. The measurement at 12 P.M. the following day resulted in a median mitoPO_2_ of 50.3 (IQR 26.5–57.1) mm Hg and 48.2 (IQR 28.1–61.8) mm Hg in respectively plaster 1 and 2. Similar results were seen at 3 P.M. the following day with a median mitoPO_2_ of 38.5 (IQR 30.1–51.8) mm Hg in plaster 1 and 40.0 (IQR 29.0–50.3) mm Hg in plaster 2. A small difference in median mitoPO_2_ was only seen between the plasters at 2 P.M., while the median mitoPO_2_ values were relatively steady over the remaining calendar time (S5 Fig).

**Table 2:** The course of the median mitoPO_2_, between-subject variability and the median within-subject variability over *calendar time* per plaster. Plaster 2 was placed 3 hours after plaster 1 was placed. Despite a difference in the value of median mitoPO_2_ at 2 P.M., the median mitoPO_2_ values seem relatively steady over the remaining calendar times. The within-subject variability remains stable over time in each plaster, while an increase in between-subject variability can be seen in plaster 2.

Calendar time in 12-hour time format	Median mitoPO_2_ in mm Hg (IQR), n = 16		Median within-subject variability in mm Hg (IQR), n = 16		Mean mitoPO_2_ in mm Hg, n = 16		Between-subject variability in mm Hg, n = 16	
	*Plaster 1*	*Plaster 2*	*Plaster 1*	*Plaster 2*	*Plaster 1*	*Plaster 2*	*Plaster 1*	*Plaster 2*
11 A.M.	49.2 (39.3–67.4)^a^	*n*.*a*.	7.9 (4.7–10.2)^a^	*n*.*a*.	51.9^a^	*n*.*a*.	21.7 ^a^	*n*.*a*.
12 P.M.	40.4 (30.4–44.5)^b^	*n*.*a*.	6.4 (5.0–12.8)^b^	*n*.*a*.	37.3^b^	*n*.*a*.	14.3 ^b^	*n*.*a*.
2 P.M.	37.5 (28.8–66.2)	45.9 (37.6–54.7)	7.6 (4.1–11.0)	6.4 (4.0–9.3)	45.1	45.1	21.9	12.2
3 P.M.	*n*.*a*.	48.5 (39.4–57.7)	*n*.*a*.	12.7 (10.5–15.1)	*n*.*a*.	47.0	*n*.*a*.	13.4
5 P.M.	45.2 (36.8–60.2)	45.5 (41.2–50.2)	7.4 (5.0–10.8)	9.9 (4.5–14.3)	46.9	44.2	19.6	15.1
12 P.M. (+1 day)	50.3 (26.5–57.1)	48.2 (28.1–61.8)	8.7 (4.9–12.8)	6.9 (4.1–13.4)	44.3	47.2	22.3	23.9
3 P.M. (+1 day)	38.5 (30.1–51.8)	40.0 (29.0–50.3)	6.3 (4.5–11.0)	8.1 (5.8–12.0)	43.6	44.8	24.8	23.5

*ALA* 5-aminolevulinic acid, *IQR* interquartile range, *n*.*a*. not applicable

^a^ There were 12 valid mitoPO_2_ measurements at this timepoint, which were included in the analyses

^b^ There were 15 valid mitoPO_2_ measurements at this timepoint which were included in the analyses

#### Plaster time

**Fig 2:**
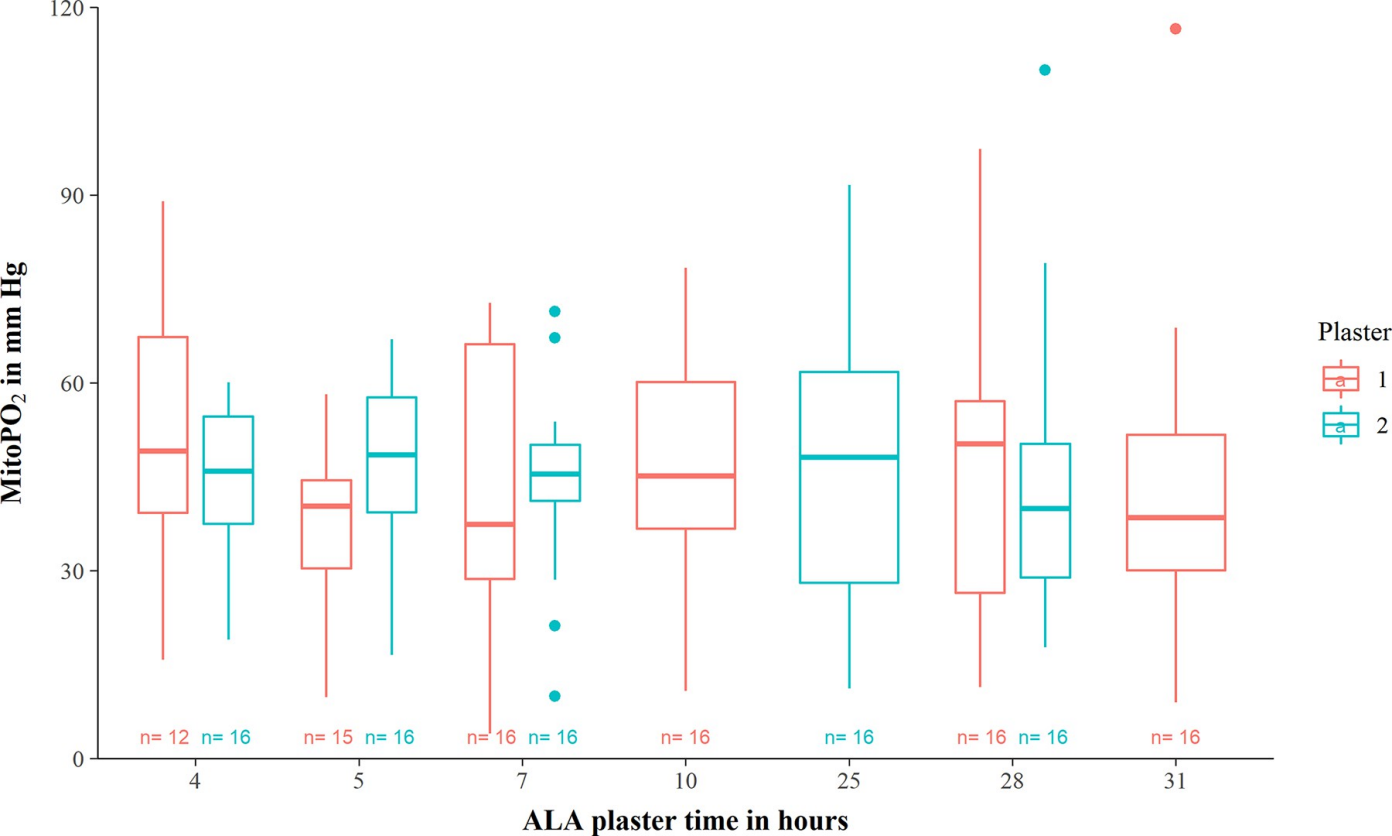
A boxplot of the course of mitoPO_2_ over ALA plaster time per plaster. The median mitoPO_2_ appears to be relatively steady over time in both plasters, despite a small difference in median mitoPO_2_ and its corresponding interquartile range after 5,7, and 28 hours ALA plaster time between plaster 1 and 2. This difference is not seen in the mean mitoPO_2_ values at that timepoint (Table 3).

The median mitoPO_2_ after 4 hours ALA plaster time was 46.6 (IQR 38.0–57.4) mm Hg, 42.4 (IQR 34.8–53.4) mm Hg after 5 hours ALA plaster time, 44.1 (IQR 33.6–56.7) mm Hg after 7 hours ALA plaster time, 45.2 (IQR 36.8–60.2) mm Hg after 10 hours ALA plaster time, 48.2 (IQR 28.1–61.8) mm Hg after 25 hours ALA plaster time, 46.3 (IQR 28.8–54.4) mm Hg after 28 hours plaster ALA time, and 38.5 (IQR 30.1–51.8) mm Hg after 31 hours ALA plaster time (Table 3 and S6 Fig). The course of the individual mitoPO_2_ values over ALA plaster time showed similar results (S7 Fig). A small difference in median mitoPO_2_ values were only seen between the plasters after 5, 7 and 28 hours ALA plaster time, while the median mitoPO_2_ values were relatively steady over the remaining calendar time (Fig 2).

**Table 3:** The course of the overall median mitoPO_2_, median within-subject variability, and between-subject variability over *ALA plaster time*. The mitoPO_2_ and within-subject variability remains stable over time, while an increase in between-subject variability can be seen after 25 hours ALA plaster time.

ALA plaster time	Median mitoPO_2_ in mm Hg (IQR), n = 32	Median within-subject variability in mm Hg (IQR), n = 32	Mean mitoPO_2_ in mm Hg, n = 32	Between-subject variability in mm Hg, n = 32
4 hours	46.6 (38.0–57.4)^a^	6.8 (4.1–10.1)^a^	48.0^a^	16.9^a^
5 hours	42.4 (34.8–53.4)^b^	11.5 (6.3–13.8)^b^	42.3^b^	14.5^b^
7 hours	44.1 (33.6–56.7)	8.0 (4.2–12.6)	44.6	18.5
10 hours	45.2 (36.8–60.2)^c^	7.4 (5.0–10.8)^c^	46.9^c^	19.6^c^
25 hours	48.2 (28.1–61.8)^d^	6.9 (4.1–13.4)^d^	47.2^d^	23.9^d^
28 hours	46.3 (28.8–54.4)	8.4 (5.5–12.6)	44.5	22.6
31 hours	38.5 (30.1–51.8)^c^	6.3 (4.5–11.0)^c^	43.6^c^	24.8^c^

*ALA* 5-aminolevulinic acid, *IQR* interquartile range, *n*.*a*. not applicable

^a^ Twelve of the sixteen mitoPO_2_ measurements were valid in ALA plaster 1 and could be included in the analysis. Total number of valid measurements included in this analysis were therefore 28.

^b^ Fifteen of the sixteen mitoPO_2_ measurements were valid in ALA plaster 1 and could be included in the analysis. Total number of valid measurements included in this analysis were therefore 31.

^c^ At this timepoint, there were only valid mitoPO_2_ measurements available from ALA plaster 1. Thus total number of valid measurements included in this analysis were therefore 16.

^d^ At this timepoint, there were only valid mitoPO_2_ measurements available from ALA plaster 2. Thus total number of valid measurements included in this analysis were therefore 16.

Overall, the value of mitoPO_2_ remained relatively stable over time, both in calendar time (Table 2, S5 and S9 Figs) and in ALA plaster time (Table 3, Fig 2 and S6 Fig).

### Within-subject variability

The individual within-subject variability and the overall median within-subject variability showed no marked increase of the within-subject variability over ALA plaster time (Tables 2 and 3, S4 Table, S8 and S10 Figs).

The within-subject variability per plaster over calendar time and ALA plaster time, showed small differences in median within-subject variability at 5 P.M. and 5 hour ALA plaster time respectively (S11–S13 Figs). S5 Table shows that the median within-subject variability after 5 hours ALA plaster time was 6.4 (IQR 5.0–12.8) mm Hg in plaster 1 and 12.7 (IQR 10.5–15.1) mm Hg in plaster 2.

The median within-subject variability over ALA plaster time was 6.8 mm Hg (IQR 4.1–10.1) mm Hg after 4 hours ALA plaster time, 11.5 (IQR 6.3–13.8) mm Hg after 5 hours ALA plaster time, 8.0 (IQR 4.2–12.6) mm Hg after 7 hours ALA plaster time, 7.4 (IQR 5.0–10.8) mm Hg after 10 hours ALA plaster time, 6.9 (IQR 4.1–13.4) mm Hg after 25 hours ALA plaster time, 8.4 (IQR 5.5–12.6) mm Hg after 28 hours ALA plaster time, and 6.3 (IQR 4.5–11.0) mm Hg after 31 hours ALA plaster time (Table 3).

### Between-subject variability

An increase in between-subject variability could be seen in calendar time from 12 P.M. next day on in both the overall between-subject variability and the between-subject variability per plaster (S14 and S15 Figs). An increase in the overall between-subject variability was observed from 17.3 mm Hg at 5 P.M. to 22.8 mm Hg at 12 P.M. the following day (S4 Table).

The between-subject variability was 16.9 mm Hg after 4 hours ALA plaster time, 14.5 mm Hg after 5 hours ALA plaster time, 18.5 mm Hg after 7 hours ALA plaster time, 19.6 mm Hg after 10 hours ALA plaster time, 23.9 mm Hg after 25 hours ALA plaster time, 22.6 mm Hg after 28 hours ALA plaster time, and 24.8 mm Hg after 31 hours ALA plaster time (Table 3). The increase of overall between-subject variability over ALA plaster time was reflected in S16 Fig, showing an increase in between-subject variability after 25 hours ALA plaster time. This increase was more pronounced in plaster 2 than in plaster 1 (S17 Fig).

### Adverse events

All healthy volunteers developed adverse events during the study measurements, as depicted in S6 Table, ranging from erythema (n = 15, 94%), itching (n = 15, 94%) to hyperpigmentation of the skin (n = 2, 13%). All adverse events resolved within 1 month after the last measurement. An overview of the adverse events and their course over time are depicted in S6 Table. Nine of the participants (56%) experienced their adverse events as only mild in unpleasantness. Six felt moderate unpleasantness due to their adverse events, while one participant did not report the severity of the adverse event. The compliance rate of the healthy volunteers with the protection instructions was 94% (n = 15). One volunteer only applied protection for one night, 10 volunteers protected the locations for 48 hours (63%), and 5 volunteers (32%) applied protection for even longer than two days, ranging from 72 hours up until a month. The manner of protection was as follows: 10 protected with clothing, 4 with the given plaster for protection at the end of the measurement moments, 1 with the plaster and clothing and 1 with clothing and sunscreen.

## Discussion

We performed a study to describe the mitoPO_2_, the within- and between subject variability of the mitoPO_2_ values during the first 31 hours after application of the ALA plaster in healthy volunteers. The mitoPO_2_ was relatively stable over a period of 28 hours of ALA plaster time with a median range of 42.4–48.2 mm Hg, while there was a decline at 31 hours ALA plaster time. Furthermore, the within-subject variability of mitoPO_2_ showed no marked increase over a period of 31 hours ALA plaster time. Interestingly the between-subject variability was slightly increased 25-hours after application of the ALA plaster.

Previous studies using the COMET system, have performed mitoPO_2_ measurements over a period of up to 6 hours ALA plaster time [13, 15–18]. Only a few of these studies have described their between-subject and/or within-subject variability of the mitoPO_2_ measurements over time, concluding moderate repeatability of the mitoPO_2_ measurements using the COMET system over a short period of time [15, 17]. As stated earlier, the repeatability is a characteristic of a measurement system indicating the precision of the measurement under similar conditions with replicate measurements over time [19]. We previously performed mitoPO_2_ measurements with 24-hours ALA plaster time using the COMET system in critically ill patients and showed an increase of both within-subject and between-subject variability after 3 hours, implicating diminished repeatability of the COMET system over a period of time [20]. However, our current results show that the within-subject variability and mitoPO_2_ measured with the COMET system are relatively repeatable up until 28 hours ALA plaster time in healthy volunteers, but that between-subject variability increases after 25 hours. We therefore recommend that the ALA plaster is replaced with a new plaster after 24 hours when mitoPO_2_ measurements with the COMET system are performed.

Biologically, 24 hours after ALA induction normalization of PpIX levels is expected, with a peak fluorescence after 4–14 hours ALA plaster time and limited PpIX fluorescence detectability after 24 hours [27, 28]. The diminished fluorescence of PpIX after 4–14 hours ALA plaster time might explain the increased between-subject variability seen in our study after 25 hours ALA plaster time. However, even after 25–31 hours ALA plaster time, the signal quality of the measurements remained above 25%, suggesting the fluorescence signal of PpIX was still substantial and not causing the increase in the between-subject variability. The biological mechanism of the increase in between-subject variability after 25 hours ALA plaster time is therefore not fully understood with the current knowledge.

Furthermore, our results showed that longer time since application of ALA plaster resulted in better signal quality, especially after 5 hours ALA plaster time, strengthening the earlier report of the correlation between ALA plaster time and the signal quality [17]. In one participant we did not gain sufficient signal quality for our measurements despite protocol adherence. Despite 7 hours of ALA plaster time, no increase in signal quality was seen. Furthermore, no drop in mitoPO_2_ values was seen in this heathy volunteer after occlusion of the microcirculation. Looking into patient characteristics, the only difference with other participants was that this participant had a dark skin tone. It is known that the depth of COMET measurement is dependent on the stratum corneum thickness [10], which has been associated with sun exposure, smoking (negative association), and pigmentation independent of sun exposure [29, 30]. Further investigation is needed to clarify these effects on the mitoPO_2_ measurement with the COMET measurement device. The manufacturer was notified, but the cause of the low signal quality was not completely understood.

Interestingly, an unexpected high incidence of adverse events was seen in our study, since 100% of the healthy volunteers developed local adverse events during the measurements. Previously reported incidence of adverse events of mitoPO_2_ measurements was 45% in healthy volunteers [31], while minimal to no adverse events have been reported in mitoPO_2_ measurements with critically ill patients [15, 20, 32]. As in previous studies, the adverse events were present at the measurement sites up to one month after the last mitoPO_2_ measurement. The higher incidence of adverse events in this study compared to the study of Harms et al. could partly be explained by the difference in measurement devices and total ALA plaster time. We used the COMET measurement device for the mitoPO_2_ measurements, while the study of Harms et al. reported measurements with the precursor of the COMET device. The total ALA plaster time in the study of Harms et al. was 5 hours, while it was 31 hours in our study [31]. No detailed information was given regarding protective measures of the PpIX induced skin in the study of Harm et al., while we used an island plaster [8]. Other studies have used an occlusive dressing or a plaster as protective measures which could contribute to variation in incidence of adverse events [8, 13]. Non-compliance to instructions, might not fully explain the high incidence of adverse events, since one participant did not protect the measurement site for at least 24 hours, which resulted in hyperpigmentation of the skin after the measurements for a month, after which the hyperpigmentation resolved. Furthermore, the cumulative dose of light at the measurement site could not explain the increased incidence of adverse events, since similar cumulative dose of light at measurement sites in critically ill patients did result in low incidence of adverse events [20]. We advise to use occlusive dressings after ALA plaster application to minimize the risk of adverse events in future mitoPO_2_ measurements with the COMET measurement device.

A strength of our study was that we strictly protocolized the measurement procedure, with strict instructions to participants, timing of measurements, dark environment during the measurements, supine position of the participants during the measurements, and the application of 2 ALA plasters 3 hours apart from each other. Therefore, the measurement conditions could have had minimal influence [19].

One of the study limitations included possible effects of small movements of the measurement probe on the within-subject variability during measurements [31], which were minimised by using gentle pressure on the cable of the measurement device to fixate the measurement probe. MitoPO_2_ measurement at the anterior chest wall in supine position strengthened the stabilization of the measurement probe despite respiratory movements of the chest wall. A double-sided plaster for fixation of the measurement probe is now handed out by the manufacturer to further minimise the effect of movement artefacts of the measurement probe on measurement variability [14, 32]. Despite no availability of double-sided plaster during the measurements in this study, the within-subject variability over time was within acceptable limits as described in previous studies [15, 17]. A within-subject variability of approximately 11.71 mm Hg was seen in replicate mitoPO_2_ measurements 10 minutes apart from each other in a study in healthy volunteers [17]. Similar results were seen in a feasibility study with critically ill patients with sepsis. Repetitive mitoPO_2_ measurements before and after local occlusion of the microcirculation resulted in within-subject variabilities of 13.62 mm Hg before occlusion and 11.24 mm Hg after occlusion [15]. However, the absolute within-subject variability was higher than seen in our pilot study, possibly due the movement artefacts [20]. We therefore advise to use the double-sided plaster to fixate the measurement probe, thereby improving the mitoPO_2_ measurement precision.

During the validation phase of the mitoPO_2_ measurements, we assumed that the local perfusion of the measurement location was not impaired in healthy volunteers. We did not perform any measurements to assess the local perfusion. In future studies, capillary refill time could be an instrument to assess the local perfusion, while keeping in mind the factors influencing the capillary refill time including ambient temperature [33, 34].

Another limitation of our study is our study size leading to broader confidence intervals, attenuating the careful interpretation of our results.

The results of this study suggest that the previously reported increased mitoPO_2_ and within-subject variability over a 24-hour time period in critically ill patients, is not a consequence of ALA plaster time. The repeatability of the mitoPO_2_ measurements seem reliable and robust in healthy volunteers, however an increased between-subject variability was seen over time. Possible explanations for the observed increased between-subject variability need to be examined in future studies [20]. Possibly it is the consequence of diminished fluorescence properties or clearance of the ALA enhanced PpIX after 24 hours [35] or other unknown biomechanical factors. Current studies with the COMET device are mostly focused on monitoring mitoPO_2_ in critically ill patients in the ICU and operation room. However, mitoPO_2_ measurements might be an additional tool in other settings as well [36]. For example, mitoPO_2_ might be an additional tool to assess sleep apnea severity, like serum Romo1 [37]. The COMET device could also be useful in assessment of mitoPO_2_ in COVID-19 patients and its possible association with balance disorders [38]. However, there is currently no evidence to support these uses and future studies into specific patient population are needed.

## Conclusions

The mitoPO_2_ values and within-subject variability remained relatively steady in healthy volunteers during the first 31 hours after ALA plaster application, with a median mitoPO_2_ of 42.4–48.2 mm Hg and within-subject variability of 6.3–11.5 mm Hg respectively, suggesting no deleterious effect of prolonged ALA plaster time. However, an increase in between-subject variability was seen after 25 hours ALA plaster time warranting replacement of the ALA plaster one day after its application. Validation of these results are needed in future studies and in different study populations.

## Supporting information

S1 Fig Overview of study procedure. (TIF)

S2 Fig Overview of the standardized mitoPO_2_ measurement per timepoint. The measurement was standardized to minimize influence of different measurement techniques on the result of the within-subject and between-subject variability.
(TIF)

S3 Fig Histograms of distribution of mitoPO_2_ over ALA plaster time. The histograms show normal distribution of mitoPO_2_ after 4 and 7 hours ALA plaster time. At remaining timepoints, no normal distribution of the mitoPO_2_ values is seen.
(TIF)

S4 Fig Histograms of distribution of mitoPO_2_ over calendar time. The histograms show normal distribution of mitoPO_2_ at 11 A.M. and 2 P.M. At remaining timepoints, no normal distribution of the mitoPO_2_ values is seen.
(TIF)

S5 Fig A boxplot of the course of mitoPO2 over calendar time per plaster. The median mitoPO_2_ appears to be relatively steady over time in both plasters, despite a small difference in median mitoPO_2_ and its corresponding interquartile range at 2 P.M. between plaster 1 and 2. This difference is not seen in the mean mitoPO_2_ values at that timepoint (Table 2).
(TIF)

S6 Fig The course of the overall median mitoPO_2_ over ALA plaster time. Concurrent measurements were performed at 4,5,7 and 28 hours ALA plaster time in plaster 1 and 2. The median mitoPO_2_ appears to be relatively steady over ALA plaster time.
(TIF)

S7 Fig Spaghetti plot of the course of the overall median mitoPO_2_ over ALA plaster time. A range of mitoPO_2_ values between 20–60 mm Hg can be seen after 4 hours ALA plaster time, which remains relatively stable up to 31 hours ALA plaster time.
(TIF)

S8 Fig Spaghetti plot of the overall median within-subject variability over ALA plaster time per participant. A range of within-subject variability between 3-13mmHg can be seen after 4 hour ALA plaster time which remains relatively steady up to 31 hours ALA plaster time. Small outliers can be seen at 10 and 25 hours ALA plaster time, probably due to measurements with only one plaster instead of 2 plasters.
(TIF)

S9 Fig The course of the overall median mitoPO_2_ over calendar time. Concurrent measurements were performed at 2 P.M., 5 P.M., 12 P.M.(+1), and 3 P.M.(+1) in plaster 1 and 2. The median mitoPO_2_ appears to be relatively steady over both calendar time.
(TIF)

S10 Fig The course of the overall median within-subject variability over ALA plaster time. Concurrent measurements were performed at 2 P.M., 5 P.M., 12 P.M.(+1), and 3 P.M.(+1) in plaster 1 and 2. The median within-subject variability appears to be relatively steady over ALA plaster time.
(TIF)

S11 Fig The course of the overall median within-subject variability over calendar time. Concurrent measurements were performed at 4,5,7 and 28 hours ALA plaster time in plaster 1 and 2. The median within-subject variability appears to be relatively steady over calendar.
(TIF)

S12 Fig The course of the within-subject variability over calendar time per plaster. The median within-subject variability appears to be relatively steady over time in both plasters. A small difference in median within-subject variability was seen at 5 P.M. between plaster 1 and 2.
(TIF)

S13 Fig The course of the within-subject variability over ALA plaster time per plaster. The median within-subject variability appears to be relatively steady over time in both plasters. A small difference in median within-subject variability was seen at 5 hours ALA plaster time between plaster 1 and 2.
(TIF)

S14 Fig The course of the overall mean mitoPO_2_ and its corresponding between-subject variability over calendar time. The dots correspond with the mean mitoPO_2_, while the line corresponds with the standard deviation and therefore the between-subject variability. Concurrent measurements were performed at 2 P.M., 5 P.M., 12 P.M.(+1), and 3 P.M.(+1) in plaster 1 and 2. The between-subject variability appears to increase after 12 P.M.(+1).
(TIF)

S15 Fig The course of the mean mitoPO_2_ and its corresponding between-subject variability over calendar time. The dots correspond with the mean mitoPO_2_, while the line corresponds with the standard deviation and therefore the between-subject variability. Concurrent measurements were performed at 2 P.M., 5 P.M., 12 P.M.(+1), and 3 P.M.(+1)in plaster 1 and 2. The between-subject variability appears to increase after 12 P.M.(+1 in especially plaster 2.
(TIF)

S16 Fig The course of the overall mean mitoPO_2_ and its corresponding between-subject variability over ALA plaster time. The dots correspond with the mean mitoPO_2_, while the line corresponds with the standard deviation and therefore the between-subject variability. Concurrent measurements were performed at 4,5,7 and 28 hours ALA plaster time in plaster 1 and 2. The between-subject variability appears to increase after 25 hours ALA plaster time.
(TIF)

S17 Fig The course of the mean mitoPO_2_ and its corresponding between-subject variability over ALA plaster time per plaster. The dots correspond with the mean mitoPO_2_, while the line corresponds with the standard deviation and therefore the between-subject variability. Concurrent measurements were performed at 4,5,7 and 28 hours ALA plaster time in plaster 1 and 2. The between-subject variability appears to increase after 25 hours ALA plaster time in especially plaster 2.
(TIF)

S1 Table Checklist of items that should be included in this manuscript. (PDF)

S2 Table An overview of characteristics of the mitoPO_2_ measurements with the COMET probe. The course of the overall median skin temperature and signal quality over ALA plaster time are depicted, as well as the median skin temperature and signal quality per plaster over ALA plaster time.
(PDF)

S3 Table Overview of missing data in the study population. There was only missing data in plaster 1 after 4 and 5 hours ALA plaster time due to poor calibration measurement performances.
(PDF)

S4 Table The course of the overall median mitoPO_2_, between-subject variability and the median within-subject variability over calendar time. Plaster 2 was placed 3 hours after plaster 1 was placed. An increase in between-subject variability can be seen after 12 P.M. the next day.
(PDF)

S5 Table The course of the median mitoPO_2_, between-subject variability and the median within-subject variability over ALA plaster time per plaster. Plaster 2 was placed 3 hours after plaster 1 was placed. The mitoPO_2_ and within-subject variability remains relatively stable over time in each plaster, while an increase in between-subject variability can be seen after 25 hours ALA plaster time in plaster 2 and after 28 hours in plaster 1.
(PDF)

S6 Table Adverse events during and after study measurements. (PDF)

S1 File A CSV file of our study database, which we used for the analyses in this manuscript. (CSV)

S2 File (PDF)
