# Supplementary figures and images for: Description of mitochondrial oxygen tension and its variability in healthy volunteers
Source: PLoS One. 2024 Jun 3;19(6):e0300602. doi: 10.1371/journal.pone.0300602 (PMC11146699; doi:10.1371/journal.pone.0300602)

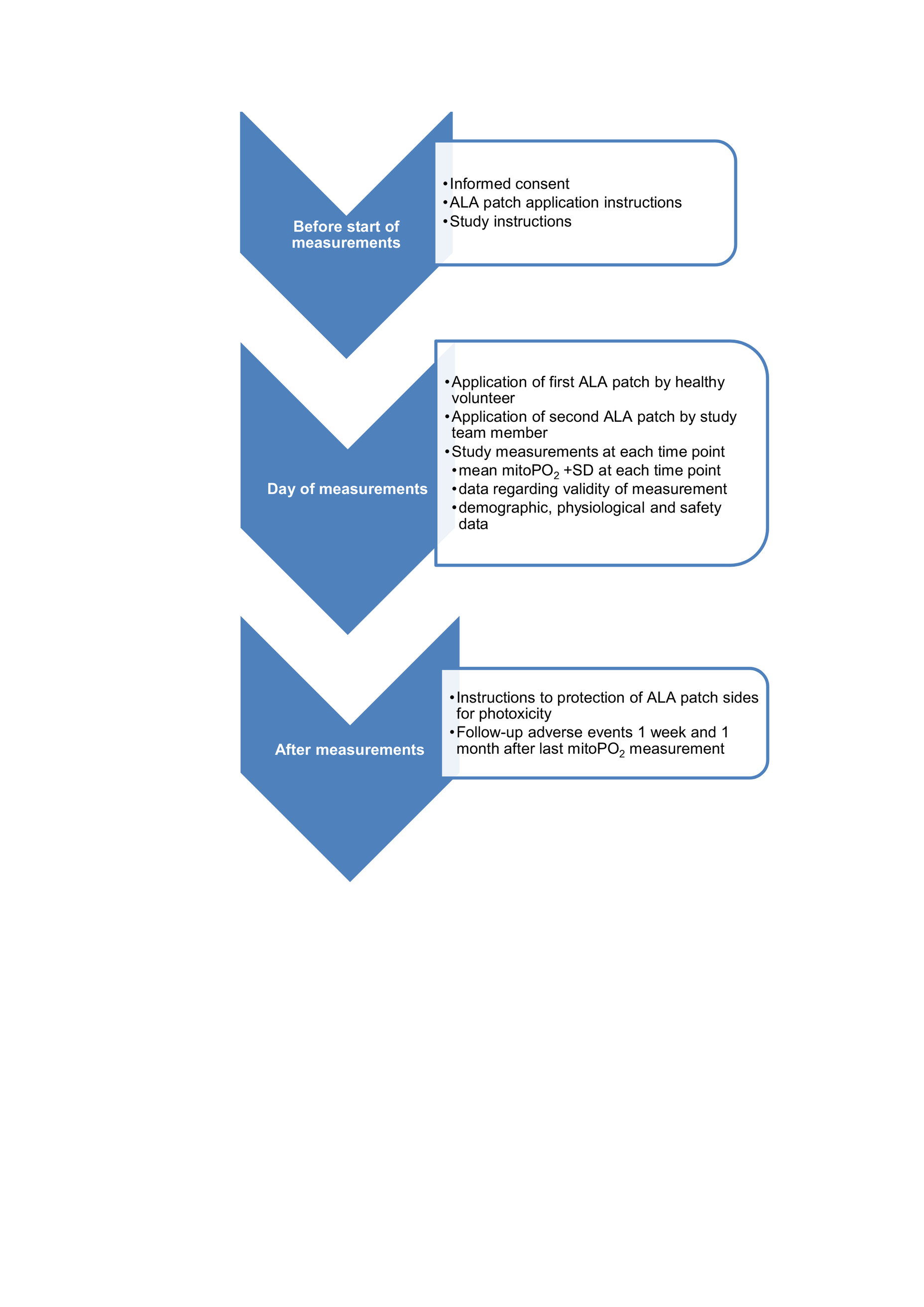

Supplement: S1 Fig — (TIF) [file pone.0300602.s001.tif]

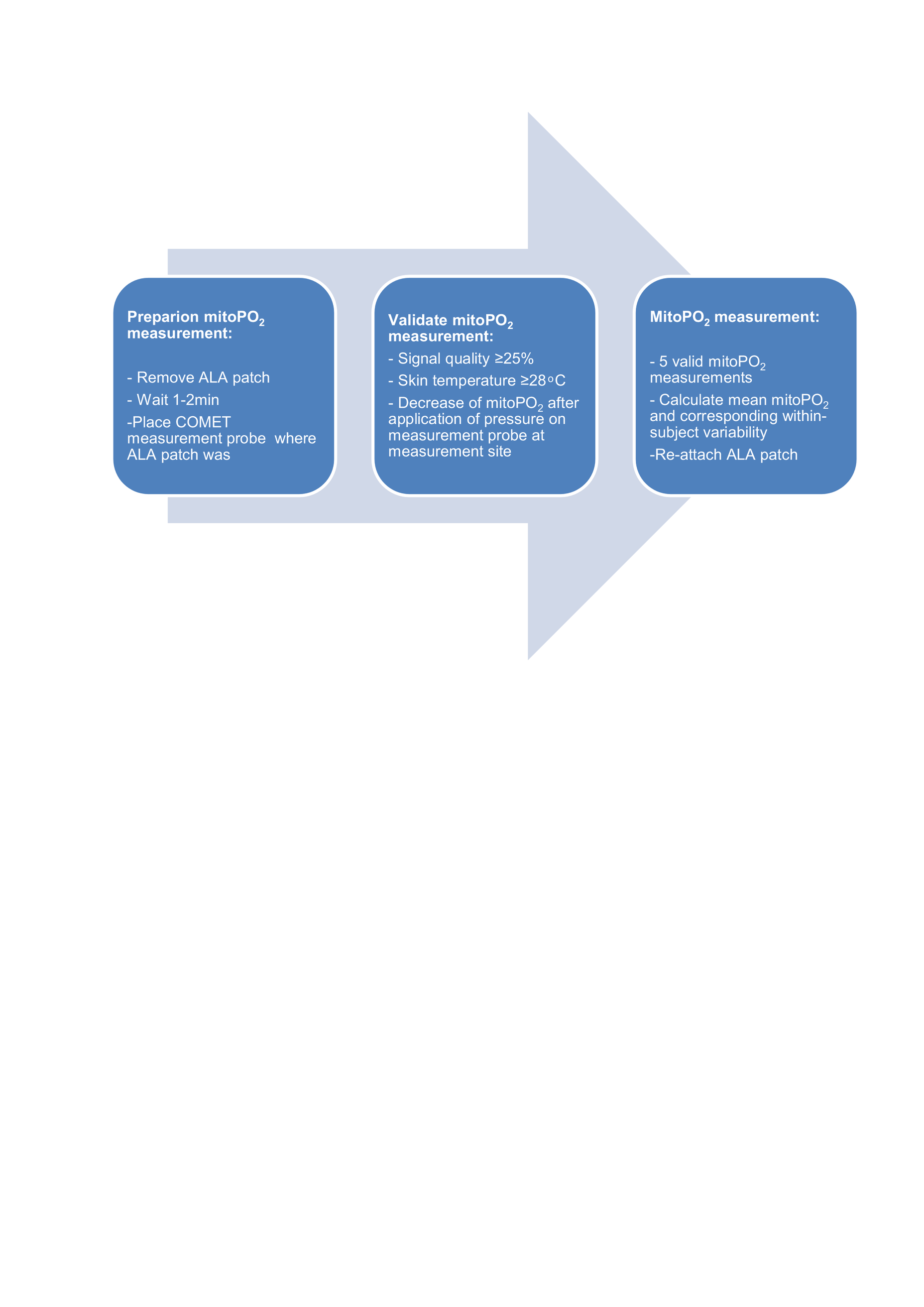

Supplement: S2 Fig — The measurement was standardized to minimize influence of different measurement techniques on the result of the within-subject and between-subject variability. (TIF) [file pone.0300602.s002.tif]

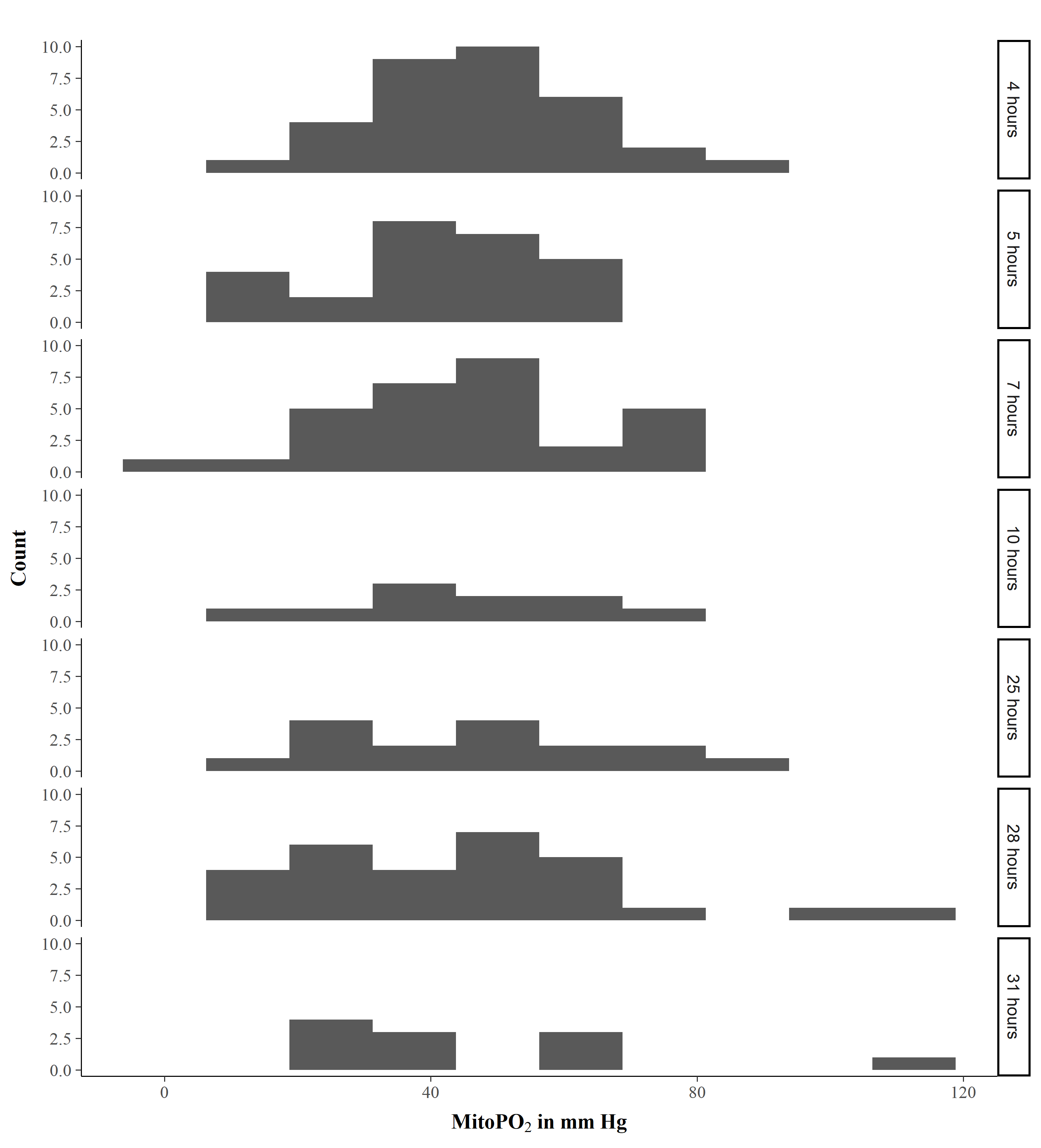

Supplement: S3 Fig — The histograms show normal distribution of mitoPO2 after 4 and 7 hours ALA plaster time. At remaining timepoints, no normal distribution of the mitoPO2 values is seen. (TIF) [file pone.0300602.s003.tif]

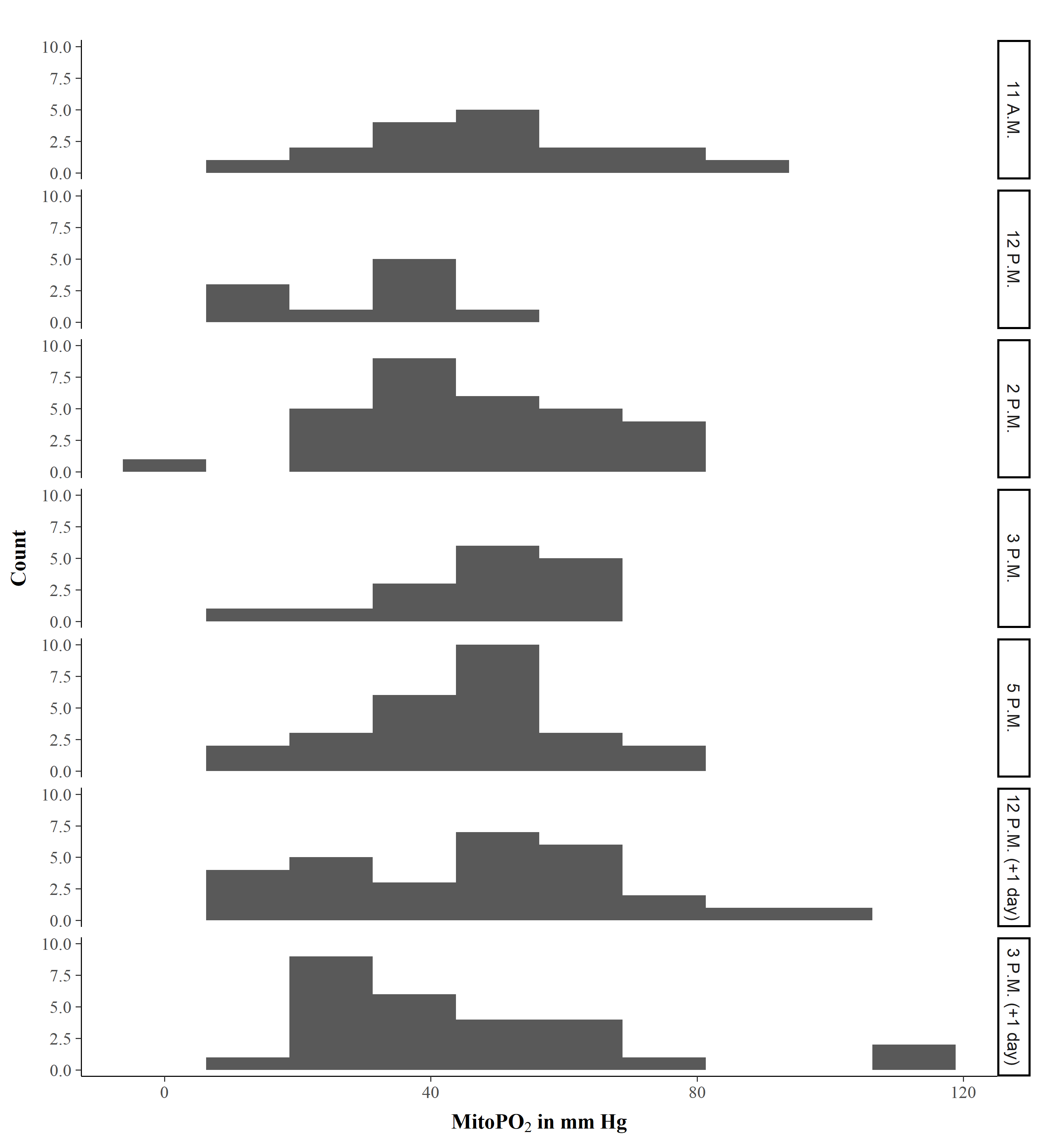

Supplement: S4 Fig — The histograms show normal distribution of mitoPO2 at 11 A.M. and 2 P.M. At remaining timepoints, no normal distribution of the mitoPO2 values is seen. (TIF) [file pone.0300602.s004.tif]

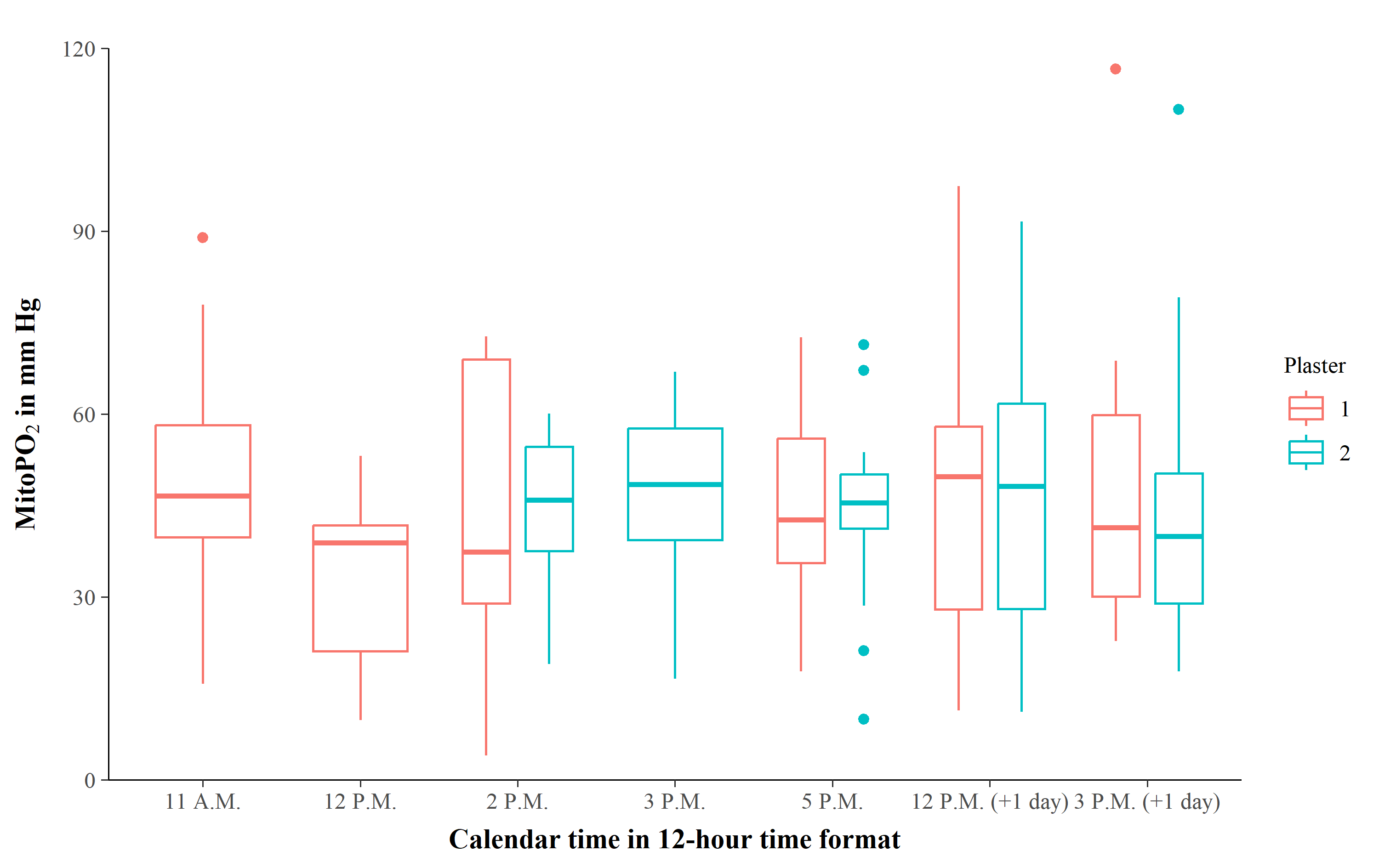

Supplement: S5 Fig — The median mitoPO2 appears to be relatively steady over time in both plasters, despite a small difference in median mitoPO2 and its corresponding interquartile range at 2 P.M. between plaster 1 and 2. This difference is not seen in the mean mitoPO2 values at that timepoint (Table 2). (TIF) [file pone.0300602.s005.tif]

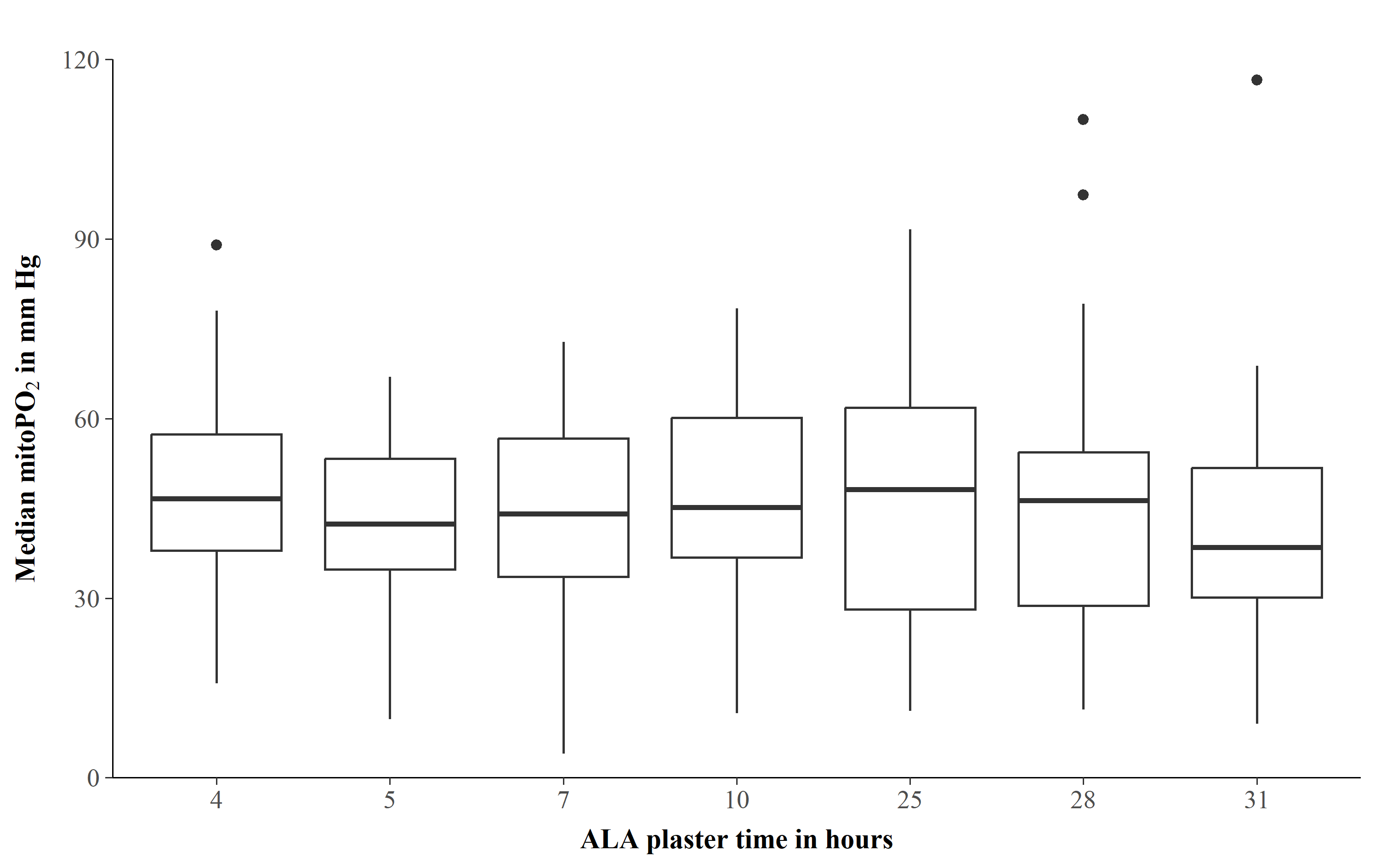

Supplement: S6 Fig — Concurrent measurements were performed at 4,5,7 and 28 hours ALA plaster time in plaster 1 and 2. The median mitoPO2 appears to be relatively steady over ALA plaster time. (TIF) [file pone.0300602.s006.tif]

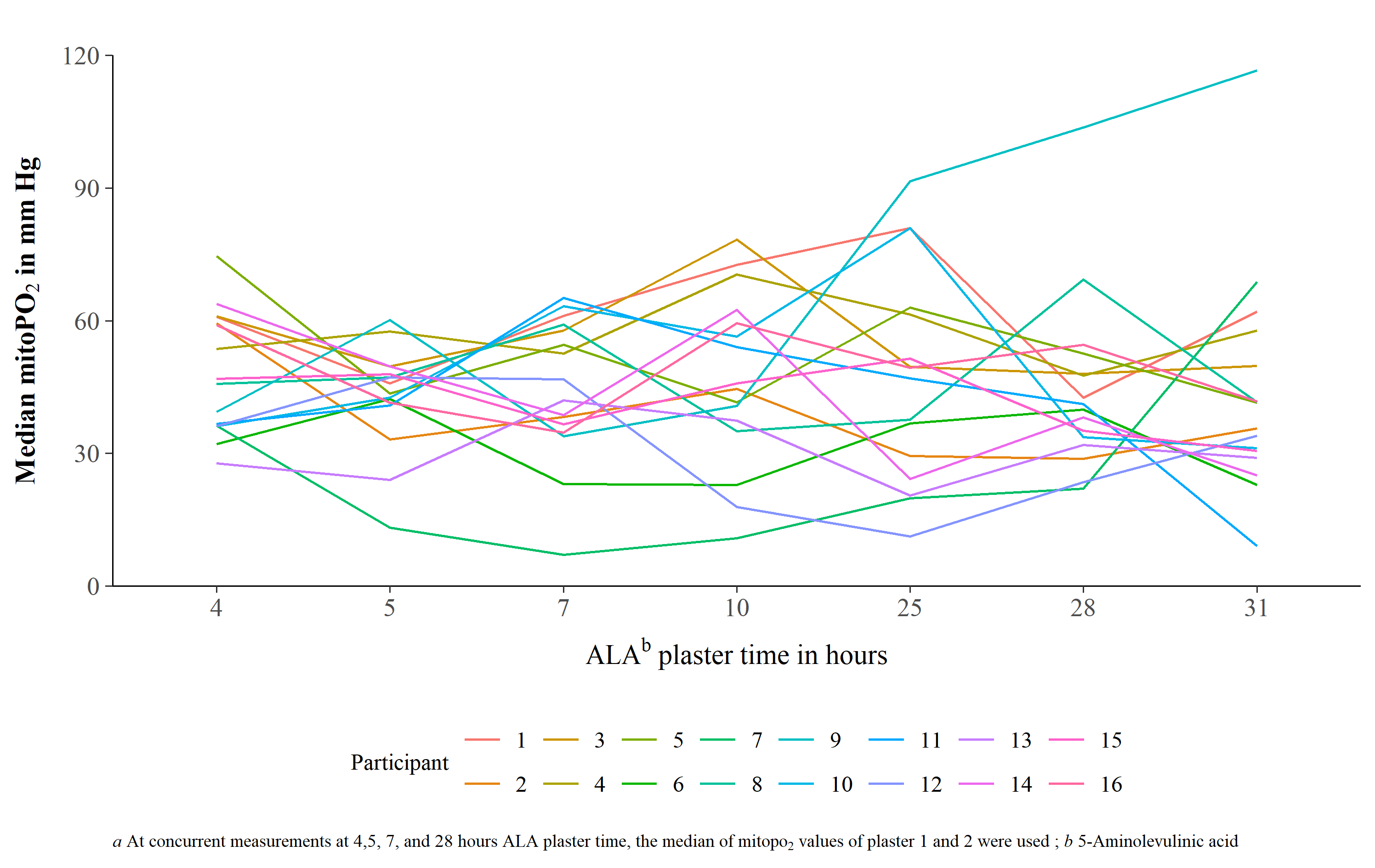

Supplement: S7 Fig — A range of mitoPO2 values between 20–60 mm Hg can be seen after 4 hours ALA plaster time, which remains relatively stable up to 31 hours ALA plaster time. (TIF) [file pone.0300602.s007.tif]

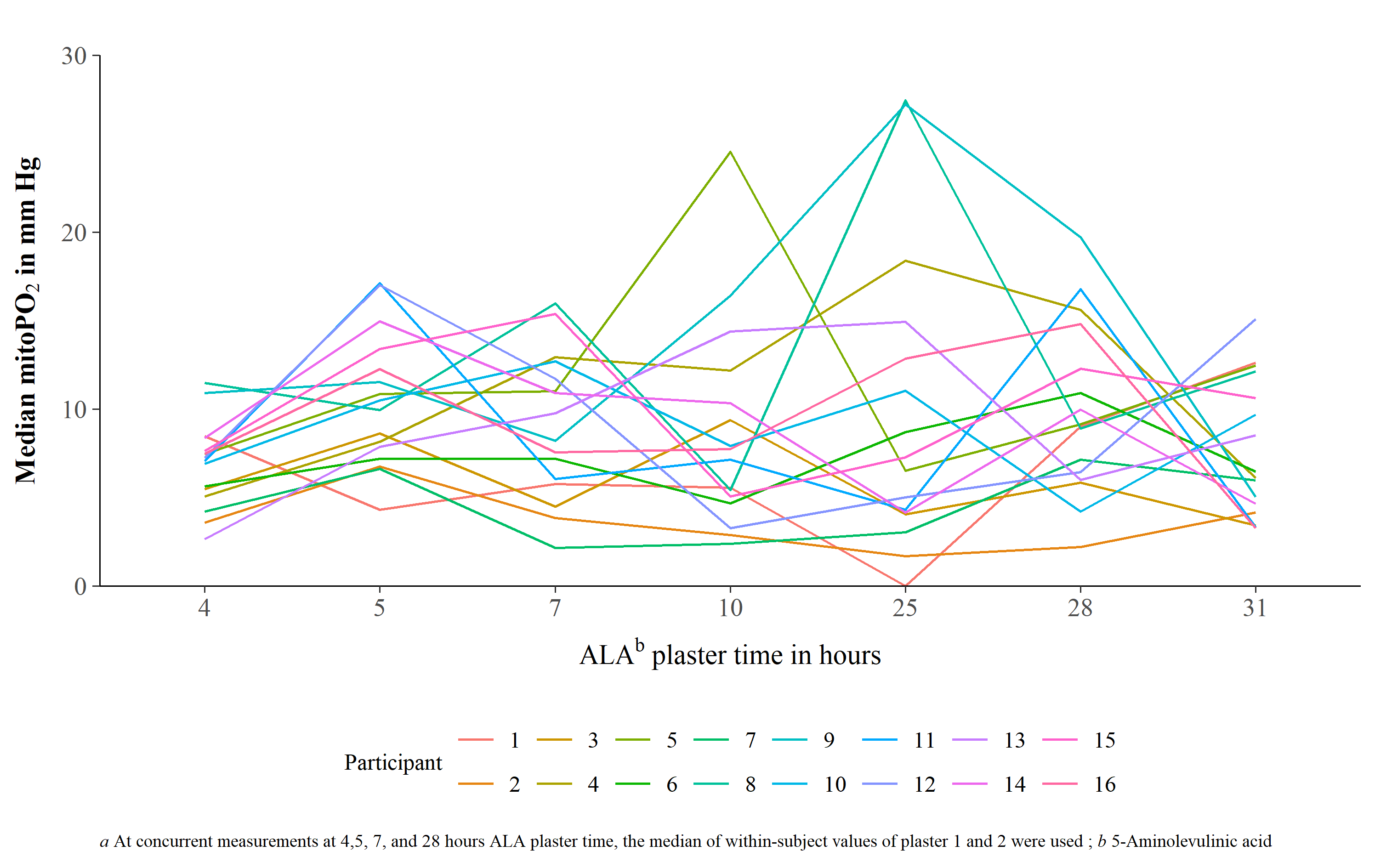

Supplement: S8 Fig — A range of within-subject variability between 3-13mmHg can be seen after 4 hour ALA plaster time which remains relatively steady up to 31 hours ALA plaster time. Small outliers can be seen at 10 and 25 hours ALA plaster time, probably due to measurements with only one plaster instead of 2 plasters. (TIF) [file pone.0300602.s008.tif]

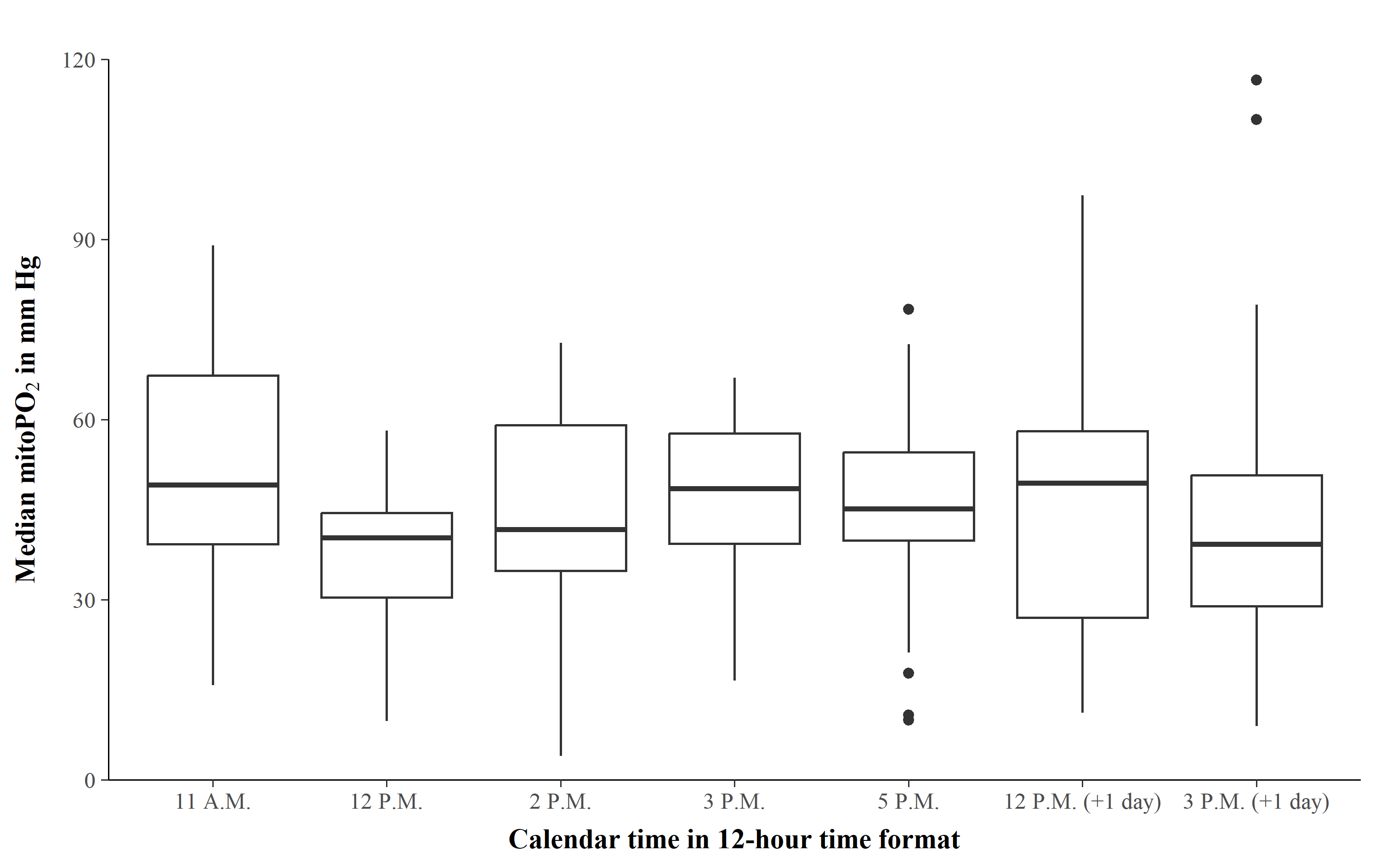

Supplement: S9 Fig — Concurrent measurements were performed at 2 P.M., 5 P.M., 12 P.M.(+1), and 3 P.M.(+1) in plaster 1 and 2. The median mitoPO2 appears to be relatively steady over both calendar time. (TIF) [file pone.0300602.s009.tif]

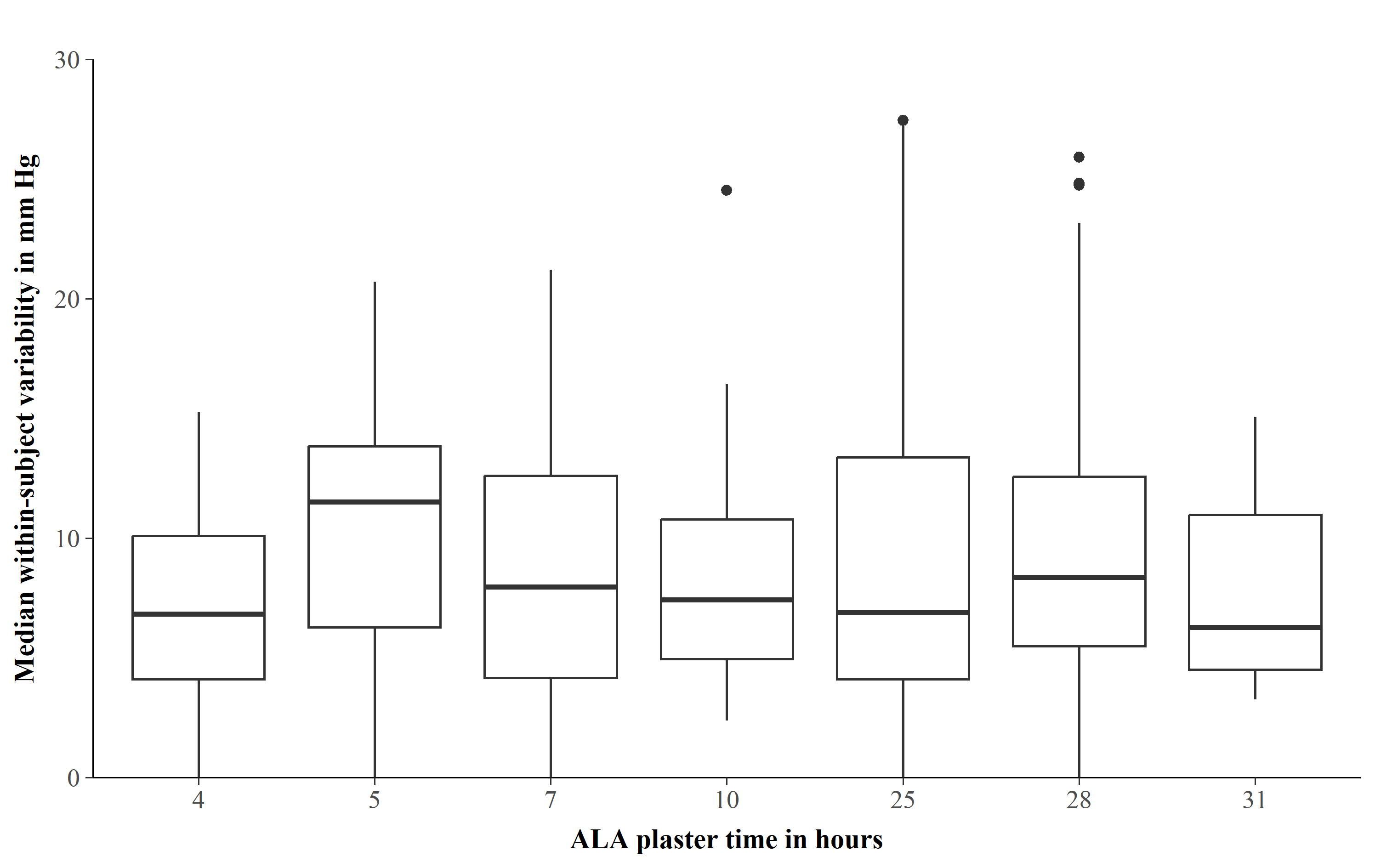

Supplement: S10 Fig — Concurrent measurements were performed at 2 P.M., 5 P.M., 12 P.M.(+1), and 3 P.M.(+1) in plaster 1 and 2. The median within-subject variability appears to be relatively steady over ALA plaster time. (TIF) [file pone.0300602.s010.tif]

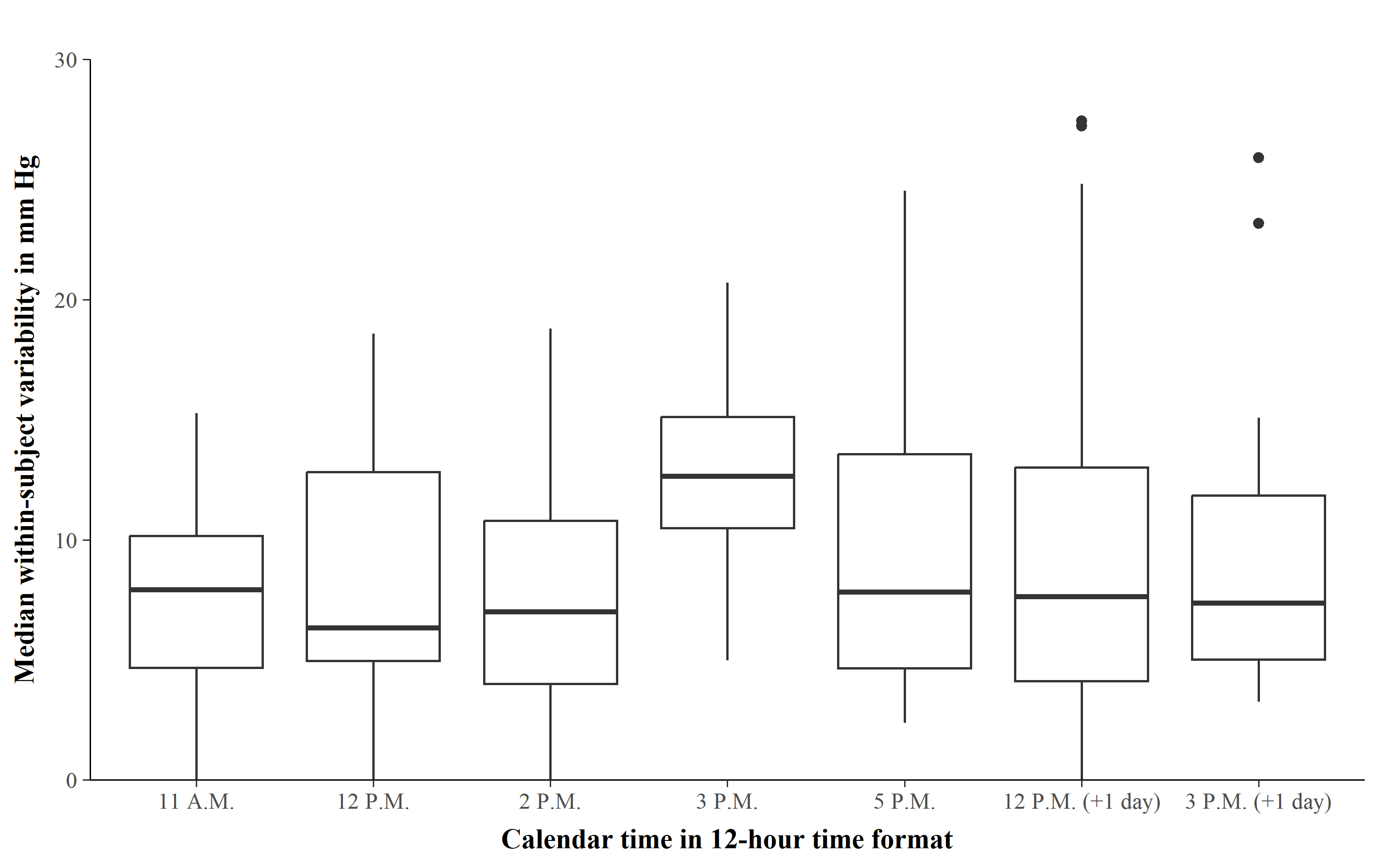

Supplement: S11 Fig — Concurrent measurements were performed at 4,5,7 and 28 hours ALA plaster time in plaster 1 and 2. The median within-subject variability appears to be relatively steady over calendar. (TIF) [file pone.0300602.s011.tif]

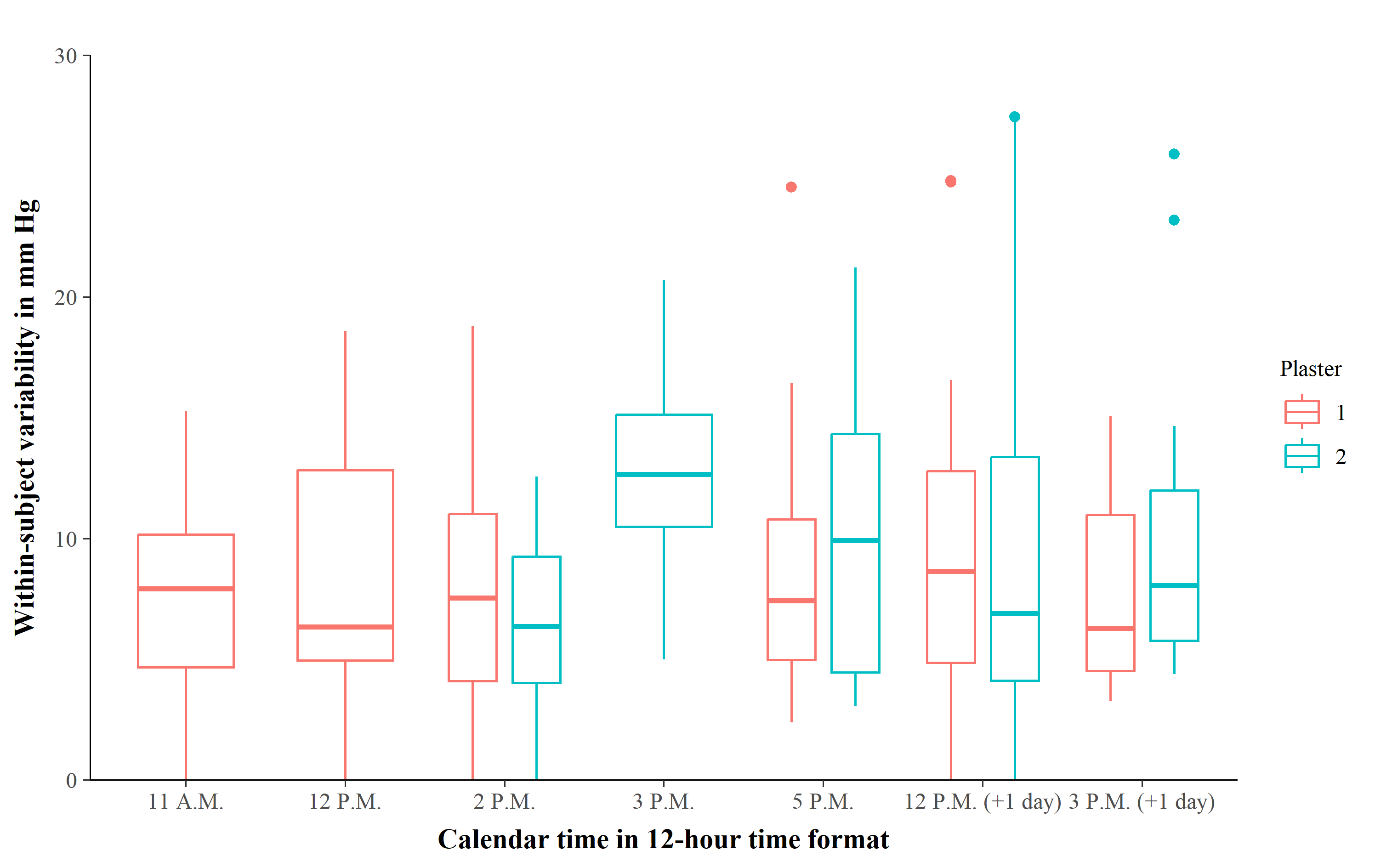

Supplement: S12 Fig — The median within-subject variability appears to be relatively steady over time in both plasters. A small difference in median within-subject variability was seen at 5 P.M. between plaster 1 and 2. (TIF) [file pone.0300602.s012.tif]

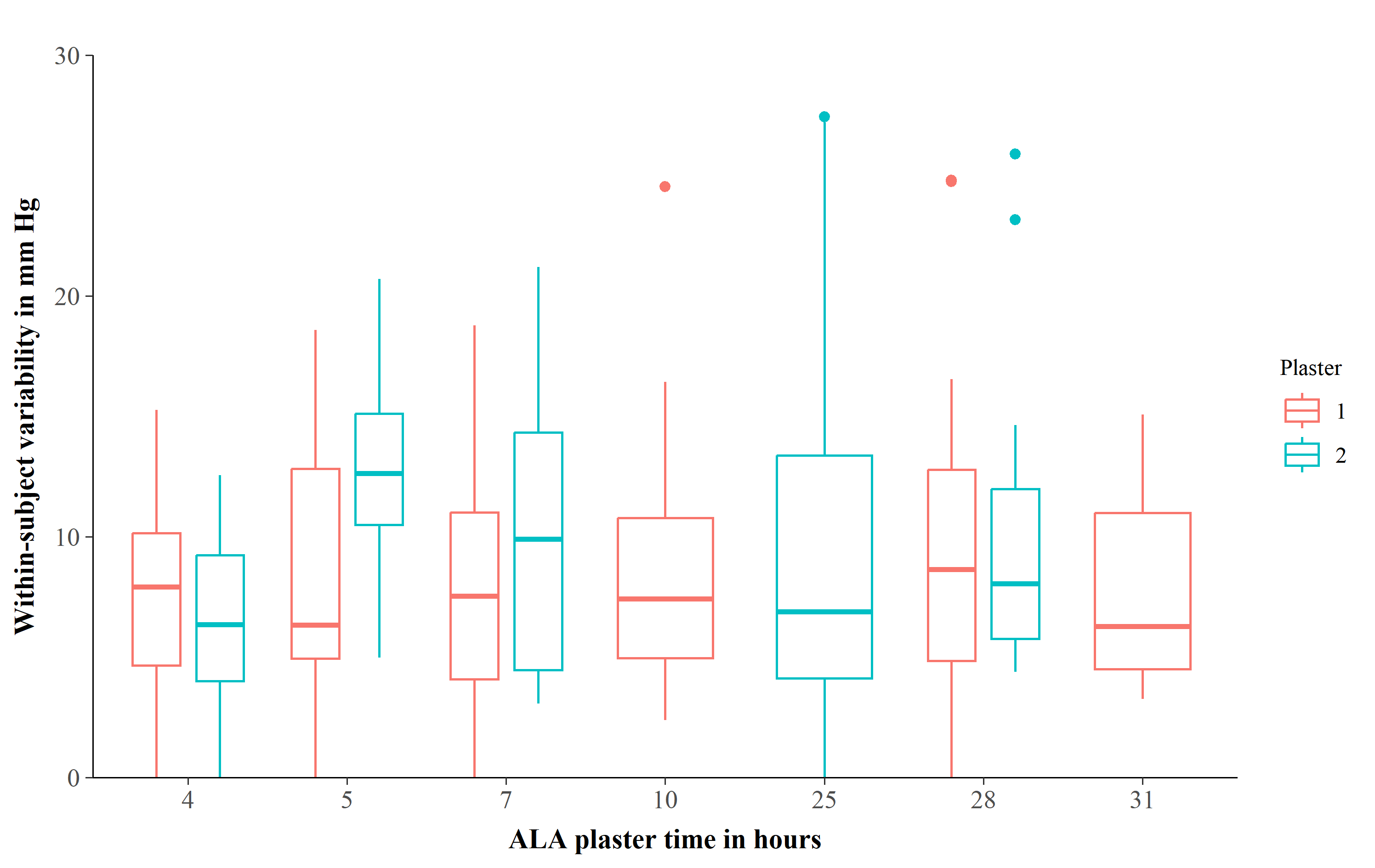

Supplement: S13 Fig — The median within-subject variability appears to be relatively steady over time in both plasters. A small difference in median within-subject variability was seen at 5 hours ALA plaster time between plaster 1 and 2. (TIF) [file pone.0300602.s013.tif]

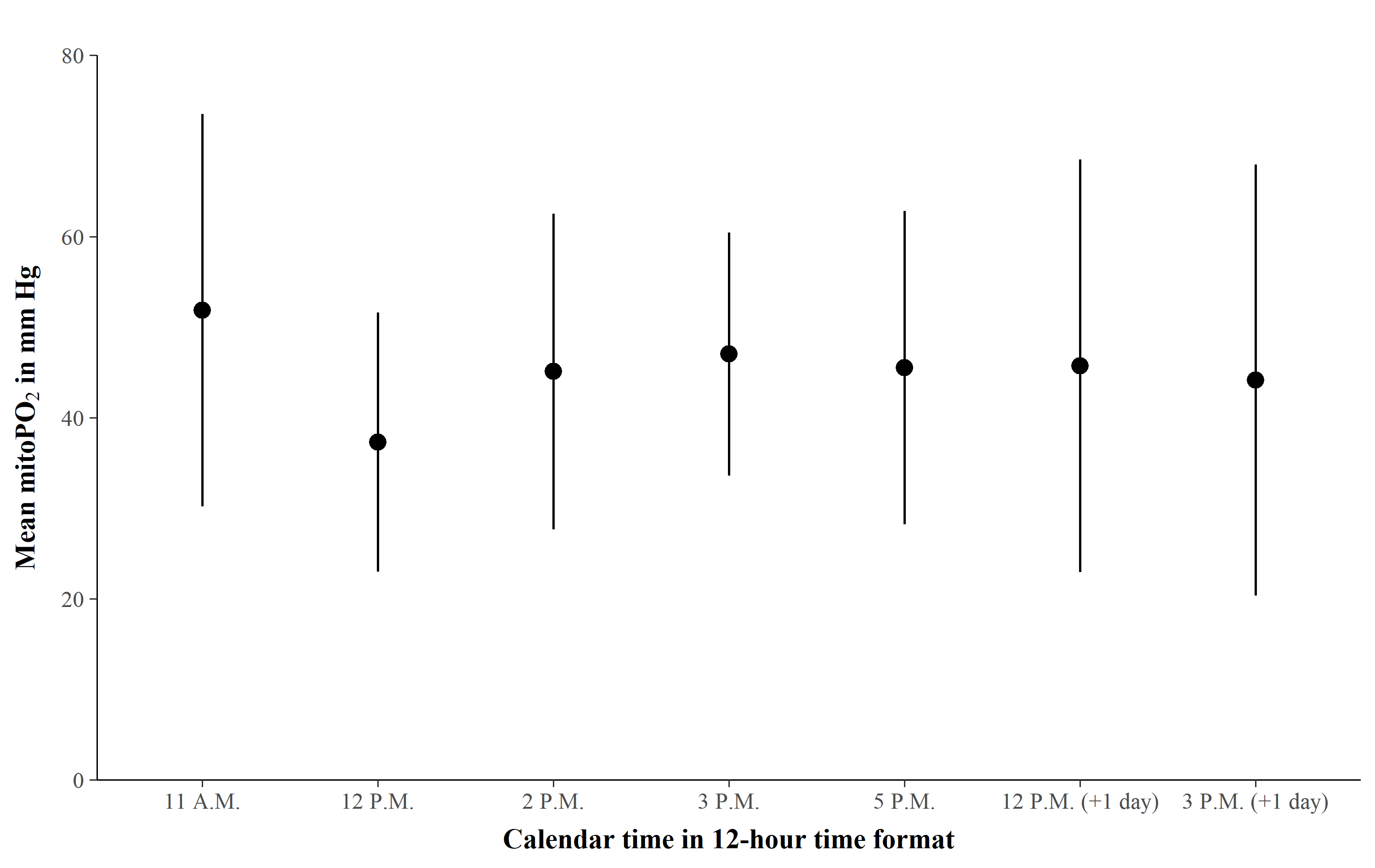

Supplement: S14 Fig — The dots correspond with the mean mitoPO2, while the line corresponds with the standard deviation and therefore the between-subject variability. Concurrent measurements were performed at 2 P.M., 5 P.M., 12 P.M.(+1), and 3 P.M.(+1) in plaster 1 and 2. The between-subject variability appears to increase after 12 P.M.(+1). (TIF) [file pone.0300602.s014.tif]

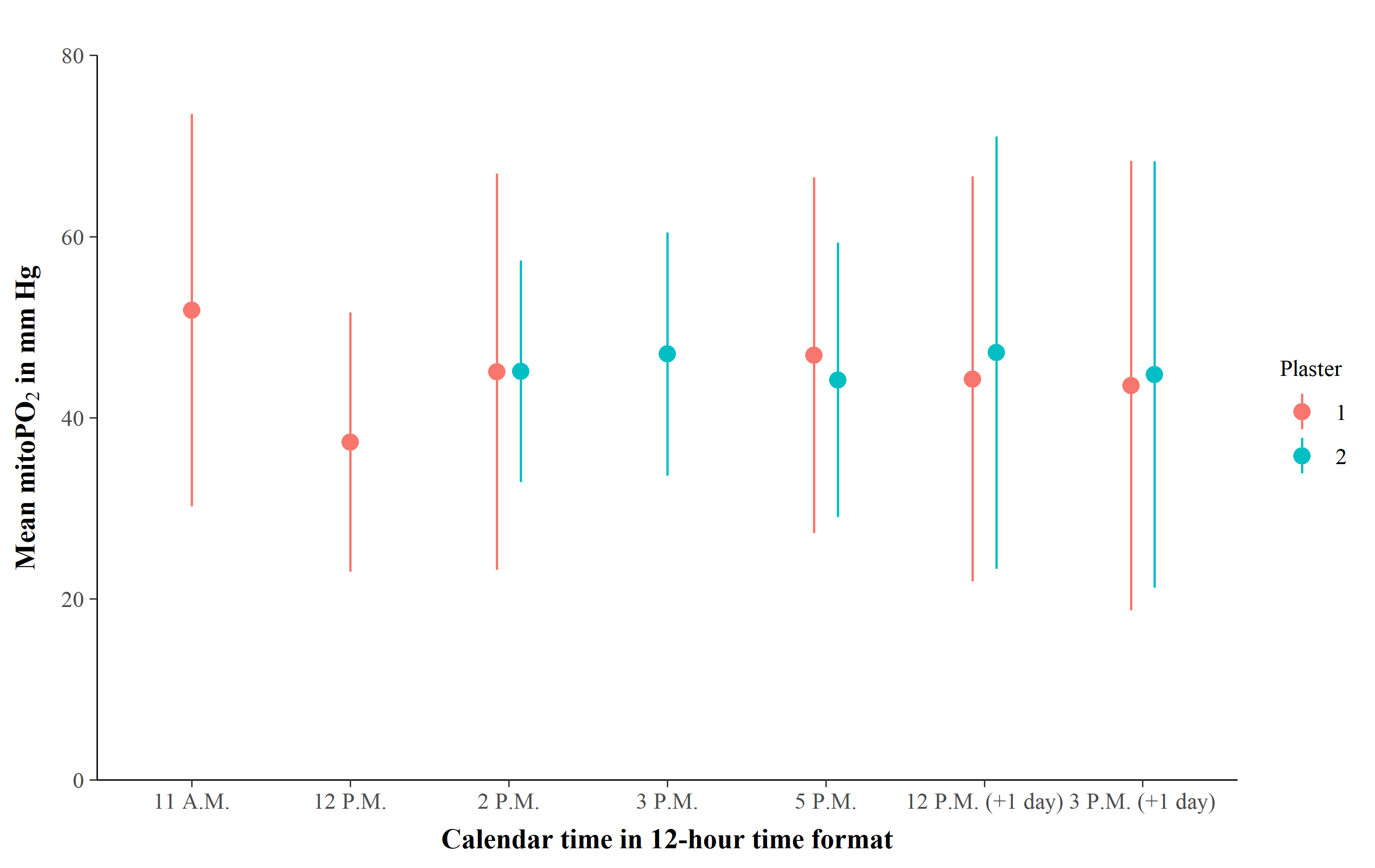

Supplement: S15 Fig — The dots correspond with the mean mitoPO2, while the line corresponds with the standard deviation and therefore the between-subject variability. Concurrent measurements were performed at 2 P.M., 5 P.M., 12 P.M.(+1), and 3 P.M.(+1)in plaster 1 and 2. The between-subject variability appears to increase after 12 P.M.(+1 in especially plaster 2. (TIF) [file pone.0300602.s015.tif]

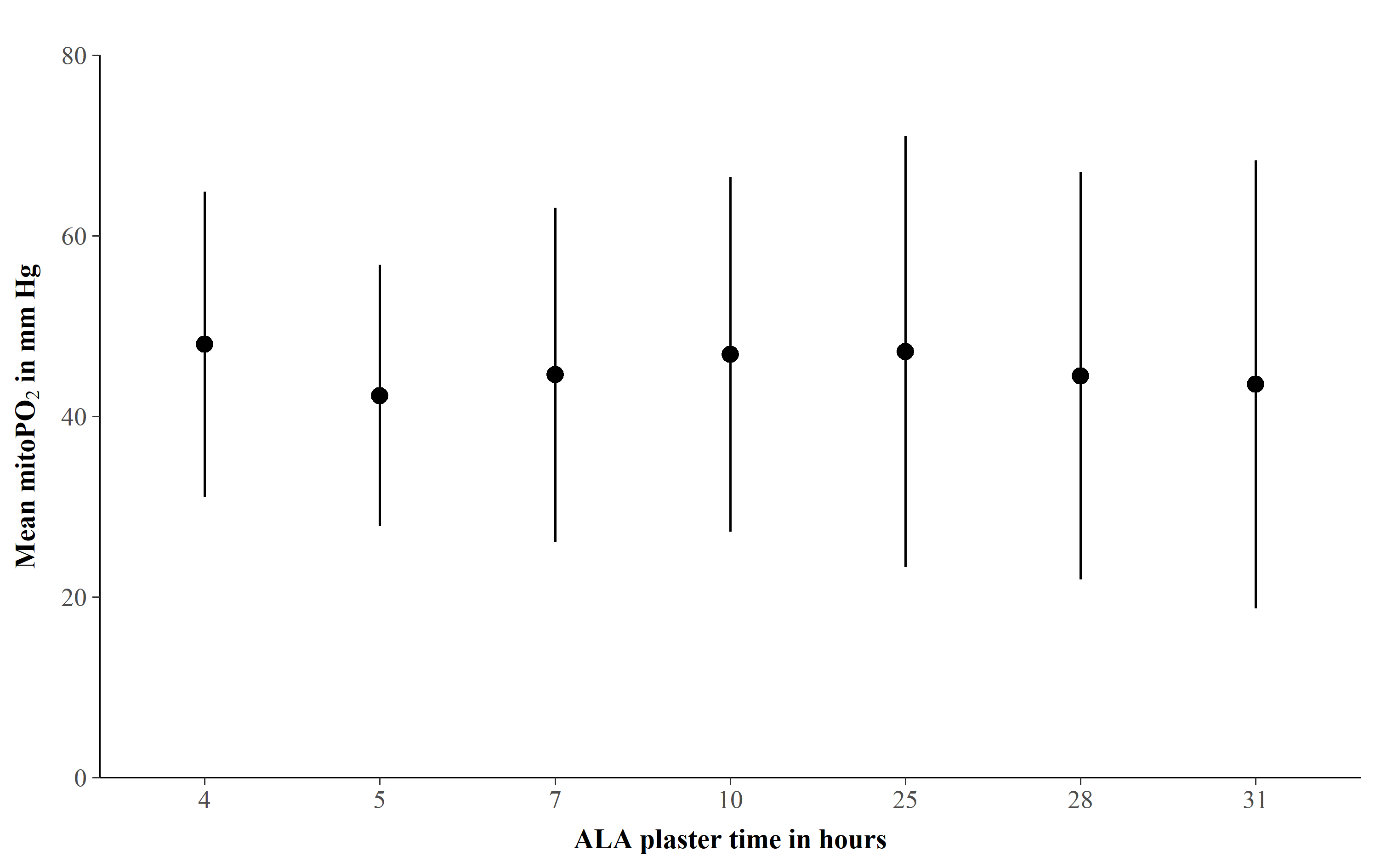

Supplement: S16 Fig — The dots correspond with the mean mitoPO2, while the line corresponds with the standard deviation and therefore the between-subject variability. Concurrent measurements were performed at 4,5,7 and 28 hours ALA plaster time in plaster 1 and 2. The between-subject variability appears to increase after 25 hours ALA plaster time. (TIF) [file pone.0300602.s016.tif]

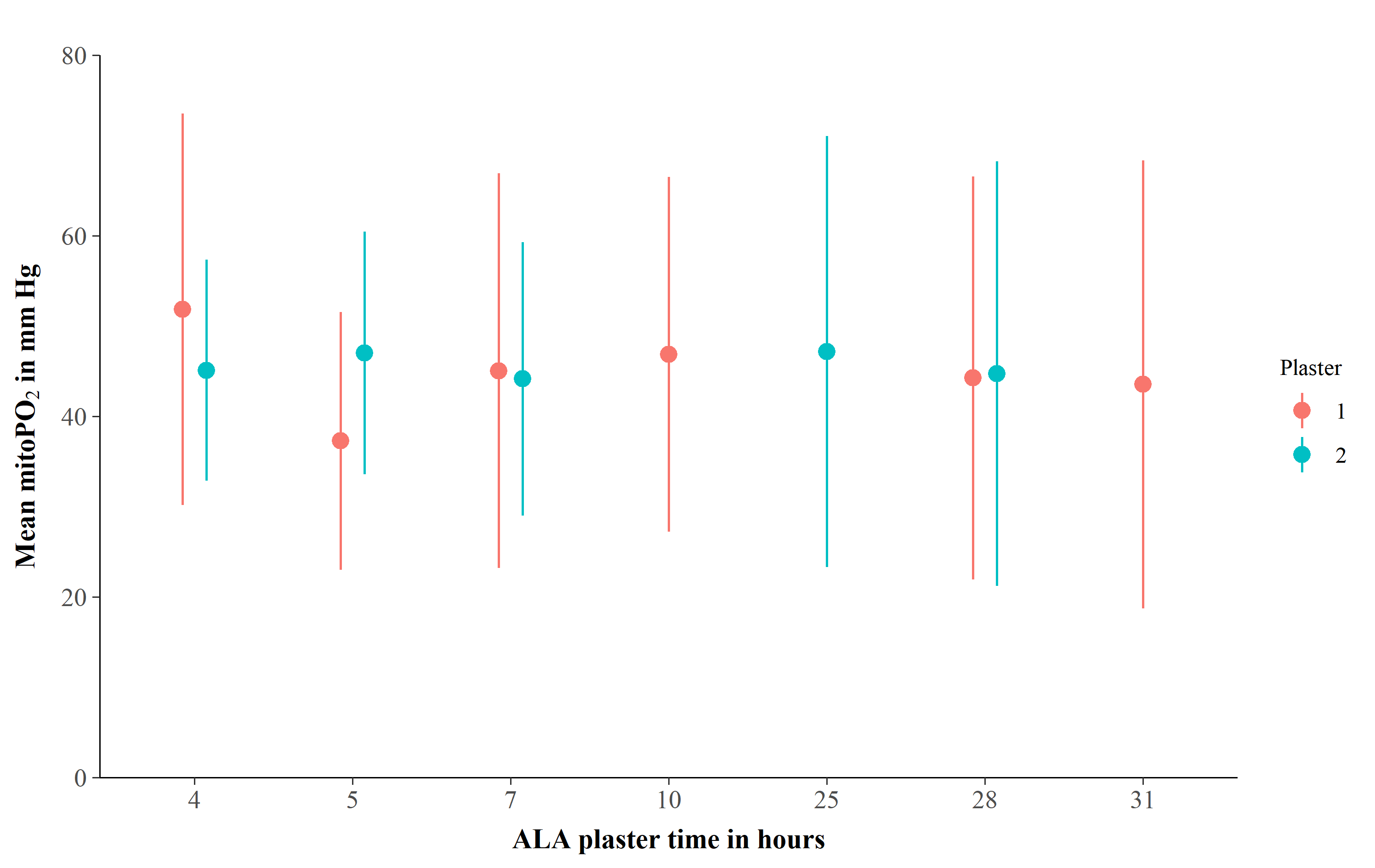

Supplement: S17 Fig — The dots correspond with the mean mitoPO2, while the line corresponds with the standard deviation and therefore the between-subject variability. Concurrent measurements were performed at 4,5,7 and 28 hours ALA plaster time in plaster 1 and 2. The between-subject variability appears to increase after 25 hours ALA plaster time in especially plaster 2. (TIF) [file pone.0300602.s017.tif]
