# Supplementary material for: Description of mitochondrial oxygen tension and its variability in healthy volunteers
Source: PLoS One. 2024 Jun 3;19(6):e0300602. doi: 10.1371/journal.pone.0300602 (PMC11146699; doi:10.1371/journal.pone.0300602)
Supplement: S2 Table — The course of the overall median skin temperature and signal quality over ALA plaster time are depicted, as well as the median skin temperature and signal quality per plaster over ALA plaster time. (PDF) [file pone.0300602.s019.pdf]

S2 Table. An overview of characteristics of the mitoPO$_2$ measurements with the COMET probe. The course of the overall median skin temperature and signal quality over ALA plaster time are depicted, as well as the median skin temperature and signal quality per plaster over ALA plaster time.

**ALA plaster time**	**Median signal quality in % (IQR)**			**Median sensor temperature in degrees Celsius (IQR)**		
	Plaster 1	*Plaster 2*	*Overall*	*Plaster 1*	*Plaster 2*	*Overall*
**4 hours**	33.3 (29.8-39.6)	30.8 (28.8-32.5)	31.6 (28.9-36.0)	30.4 (29.6-30.7)	31.8 (31.7-32.2)	31.6 (30.1-32.0)
**5 hours**	33.4 (29.5-47.4)	34.8 (30.0-42.8)	34.4 (29.6-43.0)	30.0 (29.3-31.4)	30.4 (30.1-31.3)	30.4 (29.6-31.4)
**7 hours**	41.0 (31.0-49.9)	47.8 (44.7-62.7)	45.9 (36.1-56.6)	30.7 (30.1-31.0)	31.6 (31.0-32.0)	31.0 (30.3-31.8)
**10 hours**	47.6 (43.7-63.9)	*n.a.*	47.6 (43.7-63.9)	30.3 (30.0-30.8)	*n.a.*	30.3 (30.0-30.8)
**25 hours**	*n.a.*	49.4 (40.7-58.4)	49.4 (40.7-58.4)	*n.a.*	32.0 (31.6-32.9)	32.0 (31.6-32.9)
**28 hours**	46.9 (35.5-58.7)	39.2 (35.1-50.3)	44.2 (35.1-56.6)	30.5 (29.7-31.5)	31.5 (31.0-32.3)	31.1 (30.0-32.0)
**31 hours**	38.6 (29.0-48.1)	*n.a.*	38.6 (29.0-48.1)	30.3 (29.5-30.9)	*n.a.*	30.3 (29.5-30.9)

ALA 5-aminolevulinic acid, *IQR* interquartile range, *n.a.* not applicable
