# Supplementary material for: Description of mitochondrial oxygen tension and its variability in healthy volunteers
Source: PLoS One. 2024 Jun 3;19(6):e0300602. doi: 10.1371/journal.pone.0300602 (PMC11146699; doi:10.1371/journal.pone.0300602)
Supplement: S3 Table — There was only missing data in plaster 1 after 4 and 5 hours ALA plaster time due to poor calibration measurement performances. (PDF) [file pone.0300602.s020.pdf]

S3 Table. Overview of missing data in the study population. There was only missing data in plaster 1 after 4 and 5 hours ALA plaster time due to poor calibration measurement performances.

Time point	N	Reason missing
4hr ALA plaster time plaster 1	4	- Quality of validation measurement poor* (n=3)<br>- Missed measurement (n=1)
5hr ALA plaster time plaster 1	1	Quality of validation measurement poor*

** Quality of validation measurement based on signal quality >25% and drop in mitoPO_2 with local pressure on measurement probe*
