# Supplementary material for: Description of mitochondrial oxygen tension and its variability in healthy volunteers
Source: PLoS One. 2024 Jun 3;19(6):e0300602. doi: 10.1371/journal.pone.0300602 (PMC11146699; doi:10.1371/journal.pone.0300602)
Supplement: S4 Table — Plaster 2 was placed 3 hours after plaster 1 was placed. An increase in between-subject variability can be seen after 12 P.M. the next day. (PDF) [file pone.0300602.s021.pdf]

S4 Table. The course of the overall median mitoPO$_2$, between-subject variability and the median within-subject variability over calendar time. Plaster 2 was placed 3 hours after plaster 1 was placed. An increase in between-subject variability can be seen after 12 P.M. the next day.

**Calendar time in 12-hour time format**	**Median mitoPO$_2$ in mm Hg (IQR)**	**Median within-subject variability in mm Hg (IQR)**	**Mean mitoPO$_2$ in mm Hg**	**Between-subject variability in mm Hg**
	Overall	*Overall*	*Overall*	*Overall*
11 A.M.	49.2 (39.3-67.4)	7.9 (4.7-10.2)	51.9	21.7
12 P.M.	40.4 (30.4-44.5)	6.4 (5.0-12.8)	37.3	14.3
2 P.M.	41.7 (34.9-59.1)	7.0 (4.0-10.8)	45.1	17.4
3 P.M.	48.5 (39.4-57.7)	12.7 (10.5-15.1)	47.0	13.4
5 P.M.	45.2 (39.9-54.6)	7.8 (4.7-13.6)	45.5	17.3
12 P.M. (+1 day)	49.5 (27.1-58.1)	7.7 (4.1-13.0)	45.7	22.8
3 P.M. (+1 day)	39.3 (29.0-50.8)	7.4 (5.0-11.9)	44.2	23.8

IQR interquartile range, *mitoPO$_2$* mitochondrial oxygen tension
