# Supplementary material for: Description of mitochondrial oxygen tension and its variability in healthy volunteers
Source: PLoS One. 2024 Jun 3;19(6):e0300602. doi: 10.1371/journal.pone.0300602 (PMC11146699; doi:10.1371/journal.pone.0300602)
Supplement: S5 Table — Plaster 2 was placed 3 hours after plaster 1 was placed. The mitoPO2 and within-subject variability remains relatively stable over time in each plaster, while an increase in between-subject variability can be seen after 25 hours ALA plaster time in plaster 2 and after 28 hours in plaster 1. (PDF) [file pone.0300602.s022.pdf]

S5 Table. The course of the median mitoPO$_2$, between-subject variability and the median within-subject variability over ALA plaster time per plaster. Plaster 2 was placed 3 hours after plaster 1 was placed. The mitoPO$_2$ and within-subject variability remains relatively stable over time in each plaster, while an increase in between-subject variability can be seen after 25 hours ALA plaster time in plaster 2 and after 28 hours in plaster 1.

**ALA plaster time**	**Median mitoPO$_2$ in mm Hg (IQR)**		**Median within-subject variability in mm Hg (IQR)**		**Mean mitoPO$_2$ in mm Hg**		**Between-subject variability in mm Hg**	
	Plaster 1	*Plaster 1*	*Plaster 2*	*Plaster 2*	*Plaster 1*	*Plaster 2*	*Plaster 1*	*Plaster 2*
4 hours	49.2 (39.9-67.4)	7.9 (4.7-10.2)	6.4 (4.0-9.3)	45.9 (37.6-54.7)	51.9	45.1	21.7	12.2
5 hours	40.4 (30.4-44.5)	6.4 (5.0-12.8)	12.7 (10.5-15.1)	48.5 (39.4-57.7)	37.3	47.0	14.3	13.4
7 hours	37.5 (28.8-66.2)	7.6 (4.1-11.0)	9.9 (4.5-14.3)	45.5 (41.2-50.2)	45.1	44.2	21.9	15.1
10 hours	45.2 (36.8-60.2)	7.4 (5.0-10.8)	*n.a.*	*n.a.*	46.9	*n.a.*	19.6	*n.a.*
25 hours	*n.a.*	*n.a.*	6.9 (4.1-13.4)	48.2 (28.1-61.8)	*n.a.*	47.2	*n.a.*	23.9
28 hours	50.3 (26.5-57.1)	8.7 (4.9-12.8)	8.1 (5.8-12.0)	40.0 (29.0-50.3)	44.3	44.8	22.3	23.5
31 hours	38.5 (30.1-51.8)	6.3 (4.5-11.0)	*n.a.*	*n.a.*	43.6	*n.a.*	24.8	*n.a.*

ALA 5-aminolevulinic acid, *IQR* interquartile range, *n.a.* not applicable
