# Supplementary material for: Description of mitochondrial oxygen tension and its variability in healthy volunteers
Source: PLoS One. 2024 Jun 3;19(6):e0300602. doi: 10.1371/journal.pone.0300602 (PMC11146699; doi:10.1371/journal.pone.0300602)
Supplement: S6 Table — (PDF) [file pone.0300602.s023.pdf]

S6 Table. Adverse events during and after study measurements.

**Type of adverse event**	**During the study, n (%)**	**1 week after, n (%)**	**1 month after, n (%)**
Erythema	15 (94.0%)	11 (91.7%)	5 (100.0%)
Pruritus	15 (94.0%)	1 (8.3%)	0 (0.0%)
Burning sensation of skin	4 (25.0%)	1 (8.3%)	0 (0.0%)
Skin exfoliation	5 (31.3%)	5 (41.7%)	0 (0.0%)
Crusting	3 (18.8%)	1 (8.3%)	0 (0.0%)
Hyperpigmentation	2 (12.5%)	2 (16.7%)	2 (40.0%)
**Total**	**16 (100.0%)**	**12 (75.0%)**	**5 (31.3%)**
