# Supplementary material for: Description of mitochondrial oxygen tension and its variability in healthy volunteers
Source: PLoS One. 2024 Jun 3;19(6):e0300602. doi: 10.1371/journal.pone.0300602 (PMC11146699; doi:10.1371/journal.pone.0300602)
Supplement: S2 File — (PDF) [file pone.0300602.s025.pdf]

Mitochondrial oxygen measurement variability in critically ill patients

Validation of the COMET measurement system

Coordinating investigator
Msc. M. Baysan, Department of Intensive Care, Clinical Epidemiology and Transfusion Research, Leiden University Medical Center and Sanquin Research

Principal Investigators
- Dr. M.S. Arbous, Department of Intensive Care and Department of Clinical Epidemiology, Leiden University Medical Center
- Prof. Dr. J.G. van der Bom, Department of Clinical Epidemiology and Clinical Transfusion Research, Sanquin Research

Participating investigators
- Dr. E.G. Mik, Department of Anaesthesiology, Erasmus University Medical Center
- Prof. Dr. W.C. Peul, Department of Neurosurgery, Leiden University Medical Center
- Bsc. M. Broere, Department of Intensive Care, Leiden University Medical Center

Independent Physician

Drs. J. van Paassen, Department of Intensive Care, Leiden University Medical Center

Institute: Departments of Intensive Care, Clinical Epidemiology, and Neurosurgery of the Leiden University Medical Centre, Leiden, The Netherlands

Sponsor: Leiden University Medical Center

Funding: Sanquin Research (PPOC 16-31)

ABR number: NL71914.058.19

Protocol number: P20.002

Version: 3.0

Date: 20-03-2020

Contact details

M Baysan, MD

Department of Intensive Care

Location J-04-027, LUMC

Albinusdreef 2

2333 ZA Leiden

Telephone: +31715297274

Email: M.Baysan@lumc.nl

TITLE: Mitochondrial oxygen measurement variability in critically ill patients: validation of the COMET measurement system

**Protocol ID**	P20.002
**Short title**	INOX variability
**Version**	3.0
**Date**	20-03-2020
**Coordinating investigator**	Msc. M. Baysan<br>Leiden University Medical Center and Sanquin Research Leiden<br>Department of Intensive Care, Clinical Epidemiology, and Clinical Transfusion Research<br>Plesmanlaan 1A, 2333 BZ Leiden, the Netherlands (Sanquin)<br>Albinusdreef 2, 2333 ZA Leiden, the Netherlands (LUMC)<br>**Room:** J-04-027<br>**Telephone**: +31715297274<br>**Email**: M.Baysan@lumc.nl
**Principal investigators**	Prof. Dr. J.G. van der Bom<br>Leiden University Medical Center and Sanquin Research Leiden<br>Department of Clinical Epidemiology and Clinical Transfusion Research<br>Plesmanlaan 1A, 2333 BZ Leiden, the Netherlands (Sanquin)<br>Albinusdreef 2, 2333 ZA Leiden, the Netherlands (LUMC)<br>**Room** C-07-093(LUMC)<br>**Telephone:** +31715268871<br>**Email**: J.G.van_der_Bom@lumc.nl<br><br>Dr. M.S. Arbous<br>Leiden University Medical Center<br>Department of Intensive Care and Department of Clinical Epidemiology<br>Albinusdreef 2, 2333 ZA Leiden, the Netherlands<br>**Room**: J-04-026<br>**Telephone**: +31715261678

	**Email**: M.S.Arbous@lumc.nl
**Other members research group**	Prof. Dr. W.C. Peul<br>Leiden University Medical Center<br>Department of neurosurgery<br>Albinusdreef 2, 2333 ZA Leiden, the Netherlands<br>**Room**: J-11-087<br>**Telephone**: +31715299228<br>**Email**: W.C.Peul@lumc.nl<br><br>Dr. E.G. Mik<br>Erasmus Medical Center<br>Department of Anesthesiology<br>'s-Gravendijkwal 230, 3015 CE Rotterdam, the Netherlands<br>Email: e.mik@erasmusmc.nl<br><br>Bsc. M. Broere<br>Leiden University Medical Center<br>Department of Intensive Care<br>Albinusdreef 2, 2333 ZA Leiden, the Netherlands<br>Email: M.Broere@lumc.nl
**Sponsor**	Leiden University Medical Center<br>Albinusdreef 2, 2333 ZA Leiden, the Netherlands
**Subsidising party**	Sanquin Research<br>Plesmanlaan 1A, 2333 BZ Leiden, the Netherlands
**Independent expert**	Drs. J. van Paassen<br>Department intensive care<br>Albinusdreef 2, 2333 ZA Leiden, the Netherlands<br>Room: J-04-024<br>Telephone: +31715262782<br>Email: J.van_Paassen@lumc.nl

Protocol signature sheet

Name	Signature	Date
**Coordinating Investigator:**<br><br>M. Baysan<br>PhD student<br>Department of Intensive Care, Department of Epidemiology LUMC and Sanquin Clinical Research		
**Principal Investigators:**<br><br>Prof. Dr. J.G. van der Bom<br>Epidemiologist<br>Sanquin Clinical Research and Department of Epidemiology LUMC		
**Principal Investigators:**<br><br>Dr. M.S. Arbous<br>Intensivist- Anesthetist<br>Department of Intensive Care LUMC		
**Investigator:**<br><br>Prof. Dr. W.C. Peul<br><br>Neurosurgeon<br>Department of Neurosurgery LUMC		
**Investigator:**<br><br>Dr. E.G. Mik<br>Anesthetist<br>Department of Anesthesiology Erasmus Medical Center		
**Investigator:**<br><br>M. Broere<br>Student<br>Department of Intensive Care LUMC		

Table of contents

Table of contents 5

List of abbreviations and relevant definitions 7

Summary 8

1. Introduction and rationale 10

2. Objectives 12

   2.1 General 12

   2.2 Endpoint 12

3. Design and population 13

   3.1 Study design 13

4. Study population 15

   4.1 Population (base) 15

   4.2 Inclusion criteria 15

4.3 Exclusion criteria 15

   4.4 Sample size calculation 16

5. Treatment of subjects 17

   5.1 Investigational product 17

   5.2 Use of co-intervention 18

   5.3 Escape medication 18

6. Investigational product: the COMET 19

   6.1 Name and description of medical device 19

      6.1.1 The measurement specifications 20

      6.1.2 Current stage of development 20

   6.2 Summary of known and potential risks and benefits 21

   6.3 Description and justification of route of administration and dosage 23

   6.4 Method of administration 23

   6.5 Preparation and labelling of medical device 23

7. Methods 24

   7.1 Study procedures 24

      7.1.1 General 24

      7.1.1 Clinical Care 24

   7.2 Data collection 25

      7.2.1 Sample handling and Measurements: 26

7.3 Withdrawal of individual subjects 27
8. Safety reporting 28
8.1 Section 10 WMO event 28
8.2 Possible (serious) adverse events and suspected unexpected serious adverse reactions 28
8.3 Adverse events (AEs) 28
8.4 Serious adverse events (SAEs) 29
8.5 Suspected unexpected serious adverse reactions (SUSARs) 29
8.6 Annual safety report 30
8.7 Follow-up of adverse events 30
8.8 Data Safety Monitoring Board 31
9. Statistical analysis 32
9.1 Primary study outcome 32
9.2 Secondary study outcomes 32
9.3 Statistical methods to be employed 32
10. Ethical considerations 33
10.1 Regulation Statement 33
10.2 Recruitment and Consent 33
10.3 Objection by minors or incapacitated subjects 33
10.4 Benefits and risk assessment, group relatedness 33
10.5 Compensation for injury 34
11. Administrative Aspects and Publication 35
11.1 Handling and storage of data and documents 35
11.2 Monitoring and Quality assurance 35
11.3 Amendments 35
11.4 Annual progress report 36
11.5 End of study report 36
11.6 Public disclosure and publication policy 36
12. Structured risk analysis 37
13. References 40
15. Appendix 2: Overview of measurements 44
16. Appendix 3: Monitor plan according to NFU-guidelines 46
17. Appendix 4: Postoperative neurosurgical (NCH) admissions to LUMC Medium Care (MC) / ICU 47

List of abbreviations and relevant definitions

**AE**	Adverse Event
**ALA-patch**	Cutaneous patch with 5-aminolevulinic acid
**APACHE**	Acute Physiology and Chronic Health Evaluation
**BP**	Blood pressure
**CE**	Conformité Europée ne, declaration that the medical product complies with the essential requirements of the European Directive
**COMET**	Cellular Oxygen METabolism
**DSMB**	Data Safety Monitoring Board
**EPD**	Electronic patient dossier
**ICH-GCP**	International Conference on Harmonisation of Technical Requirements for Registration of Pharmaceuticals for Human Use - Good Clinical Practice guideline
**HF**	Heart frequency
**ICU**	Intensive Care Unit
**MC**	Medium Care
**METC**	Medical research ethics committee (MREC); in Dutch: medisch ethische toetsing commissie (METC)
**$MitoPO_2$**	Mitochondrial oxygen tension
**NFU**	Nederlandse Federatie Universitair Medisch Centra
**P(a)O2**	Arterial oxygen tension
**PpIX**	Protoporphyine IX
**(S)AE**	(Serious) Adverse Event
**SaO2**	Arterial saturation
**SpO2**	Peripheral measured oxygen saturation
**Sponsor**	The sponsor is the party that commissions the organization or performance of the research, for example a pharmaceutical company, academic hospital, scientific organization or investigator. A party that provides funding for a study but does not commission it is not regarded as the sponsor, but referred to as a subsidizing party.
**TSLT**	Triplet State Lifetime Technique (protoporphyrin IX-triplet state lifetime technique)
**WMO**	Medical Research Involving Human Subjects Act (in Dutch: Wet Medisch-wetenschappelijk Onderzoek met Mensen

Summary

Rationale: Recent studies have shown the potential of a protoporphyrin IX-triple state lifetime technique to measure mitochondrial oxygen tension (mitoPO_2) in vivo, which possibly is an early indicator of oxygen disbalance in the cell. With the advent of the COMET measurement system, steps have been made to determine the feasibility of this measurement method. The INOX ICU-2 study (parent study) aims to tailor transfusion therapies to individual intensive care unit (ICU) patients based on mitochondrial oxygen tension. In the pilot study of the INOX ICU-2 study, in which the COMET measurement system was used on critically ill patients receiving red blood cell transfusion, an increase in the between- and within-subject variability was observed over time. This deviation was not explored during the development of the COMET measurement system. Therefore, we aim to determine the between- and within-subject variability of this measurement in healthy subjects and in hemodynamically stable subjects at the intensive care unit.

Main objectives: To describe the between- and within-subject variability of mitoPO_2 measurements during a 24 hour period after 5- aminolevulinic acid (ALA)-induction among healthy volunteers and among neurosurgical patients admitted postoperatively to the ICU or MC. Healthy subjects allow for the exploration of the effect of time-since-application of ALA-patch and neurosurgical patients allow for the exploration of a possible effect of ICU-admittance.

Study design: Prospective cohort study.
Study population: Healthy volunteers and neurosurgical patients admitted at the ICU or Medium Care(MC) of LUMC after surgery.

Main study endpoints:
Primary endpoint:

- Between- and within-subject variability (assessed by standard deviation) of mitoPO_2 measurements over a period of 24 hours after ALA-induction.
    - Initially in healthy volunteers
    - Followed by neurosurgical patients admitted postoperatively to the ICU or MC.

Secondary endpoints:

- Description of the differences in the between- and within-subject variability between 3 hour offset ALA-patches.
- Description of between- and within-subject variability in healthy volunteers and neurosurgical patients in relation to the between- and within-subject variability of the INOX ICU-2 pilot study (P16.303)
- Report on adverse and serious adverse events of the mitoPO_2 measurements.

Study procedure: MitoPO_2 measurements will be taken in healthy volunteers using two ALA-patches offset to each other by 3 hours, this has been done to observe possible differences in measurements at different time-points after ALA-induction while maintaining other variables stable. Following the healthy volunteers, neurosurgical patients will be included. Patients will undergo neurosurgery as planned and common practice is that no major interventions are needed in the postoperative phase on the ICU/MC. At multiple predefined moments after surgery, mitoPO_2 measurements will take place, again using two ALA-patches with a 3 hour offset.

Nature and extent of the burden and risks associated with participation, benefit and group relatedness: The risks are small in this study with no serious adverse events (SAE) known. The burden for participants is small since it involves a non-invasive measurement. We will perform this study first in health volunteers. Following the healthy volunteers, neurosurgical patients will be included. Patients will undergo neurosurgery as planned and common practice is that no major interventions are needed in the postoperative phase on the ICU. Normal clinical practice will continue and will not be altered. These patients will allow for the exploration of a possible effect of ICU admittance.

1. Introduction and rationale

Adequate tissue oxygenation is one of the cornerstones of therapy in critical care medicine, which is guided by intensive monitoring.[1] Monitors are used to assess global oxygen delivery and thus the macrocirculation.[2] However, tissue oxygenation is multifactorial and depends not only on the macrocirculation but also on the microcirculation and local cellular oxygen consumption.[3] In critically ill intensive care unit (ICU) or medium care (MC) patients disturbances in the microcirculation may lead to cellular hypoxia. While it is currently possible to monitor cellular oxygenation, these techniques are still in their infancy.[4 5]

Mik et al. have recently introduced the protoporphyrin IX (PpIX)-triplet state lifetime technique as the first method to measure mitochondrial oxygen tension (mitoPO_2) in living cells and tissues.[6] Experimental results have shown mitoPO_2's robustness and clinical potential as a parameter for cellular function. [6-9] Animal research has shown that mitoPO_2 is a capable predictor of lactate increase.[10-12] With the advent of the COMET measurement system, bedside monitoring of mitoPO_2 might allow for therapies to be tailored to individual patients.

During the pilot study of the ongoing INOX ICU-2 study an increase in the variation of mitoPO_2 results within one subject at each time point (within-subject variability) was observed the longer the 5-aminoulinic acid (ALA)-patch was in place (see appendix 1, table 1). The mean within-subject variability was 3.0 mmHg at baseline (before red blood cell (RBC) transfusion), 3.5 mmHg 2 hours after RBC transfusion and eventually increased to 7.3 mmHg 3 hours after RBC transfusion. Twenty-four hours after RBC transfusion an even higher within-subject variability of 8.7 mmHg was seen. Similarly, the variation over the group at each time point of mitoPO2 measurements (between-subject variability) seemed stable initially, but increased after 2 hours. The mean between-subject variability was 13 mmHg at baseline, 15.7 mmHg in the first hour after transfusion and gradually increased to 28.2 mmHg after 3 hours, up to 42.5 mmHg after 24 hours (see appendix 1, table 1 and figure 1).

After this result, we hypothesized why the within-subject variability increased over time. The increased within-subject variability might have been the consequence of a decreased measurement sensitivity, since animal studies have shown normalization of PpIX levels after 24 hours of ALA induction [13], thereby limiting the PpIX phosphorene detectability. However, this was probably not the case, as the median signal quality after 24 hours was still above the required 30% (median 46, IQR 34-64.5%). The increased between-subject variability might have been the consequence of different changes in the oxygen supply to the cells in the different patients over time as well. It is known that the intracellular oxygen content is dependent on multiple factors [6]. This oxygen supply can change dramatically in 24 hours due to the underlying disease and multiple other interventions besides the red blood cell transfusion [14].

To determine the reason of the increased within-subject variability, it would be ideal to repeat the measurements in healthy volunteers and in a cohort of patients admitted to the ICU that are expected to receive minimal interventions. In the Leiden University Medical Center, neurosurgical patients are admitted to the ICU or MC postoperatively. This cohort of patients consists of the most hemodynamically stable patients and receives the least amount of interventions compared to other cohorts of patients in the ICU or MC. Therefore, the cohort of neurosurgical patients would be ideal to investigate the course of the between- and within-subject variability of mitoPO_2 during a period of 24 hours. Measurements will initially be carried out in a cohort of healthy volunteers to evaluate the effect of time-since-application and continued in the neurosurgical patients to factor in the effect of ICU admittance, including the standard interventions patients admitted to the ICU receive. In the pilot a large increase in the between- and within-subject variability was observed after 3 hours. To account for influence of the ALA-patch on the measurement variability, 2 ALA-patches will be used offset relative to each other by 3 hours. This offset of 3 hours was chosen since an increase in between- and within-subject variability was observed after 3 hours in the INOX ICU-2 pilot study.

2. Objectives

2.1 General

The aim of the this project is to describe the between- and within-subject variability, as assessed by the standard deviation, of mitoPO_2 measurements during a period of 24 hours after ALA-induction with the COMET measurement system (see section 7.2), initially in healthy volunteers, followed by postoperative neurosurgical patients admitted to the ICU or MC. Healthy subjects allow for the exploration of the effect of time-since-application of ALA-patch and neurosurgical patients allow for the exploration of a possible effect of ICU-admittance, including the effect of interventions patients admitted to the ICU receive. Furthermore, we will compare this to the already observed change over 24 hours of mitoPO_2 in patients who received red blood cell transfusion in the ICU.

2.2 Endpoint

In this descriptive study we aim to investigate mitoPO_2's variability over the 24 hour period after ALA-induction

Specific objectives:

1. To describe the between- and within-subject variability (assessed by standard deviation) of mitoPO_2 measurements over a period of 24 hours after ALA-induction.
    a. Initially in healthy volunteers
    b. Followed by neurosurgical patients admitted postoperatively to the ICU or MC.
2. To describe the between- and within-subject variability between 3 hour offset ALA-patches.
3. Description of between- and within-subject variability in healthy volunteers and neurosurgical patients in relation to the between- and within-subject variability of the INOX ICU-2 pilot study (P16.303).
4. Report on adverse and serious adverse events of the mitoPO_2 measurements.

3. Design and population

3.1 Study design

We will prospectively study the between- and within-subject variability of mitoPO$_2$ measurements in the Leiden University Medical Center (LUMC). Initially, measurements will be taken among 17 healthy volunteers at predefined moments over a 24 hour period, to observe the between- and within-subject variability. Subsequently, clinical measurements will commence to factor in the critical care setting. Measurements will be taken among 17 patients, admitted to the ICU or MC after neurosurgery, at predefined moments during the first 24 hours after admittance. No specific interventions besides standard care will be performed. During the study, nurses and clinicians will be blinded for the results of the mitoPO$_2$, since the investigators execute the mitoPO$_2$ measurements and will not reveal the results.

Figure 1: Gross overview of the study design on healthy volunteers

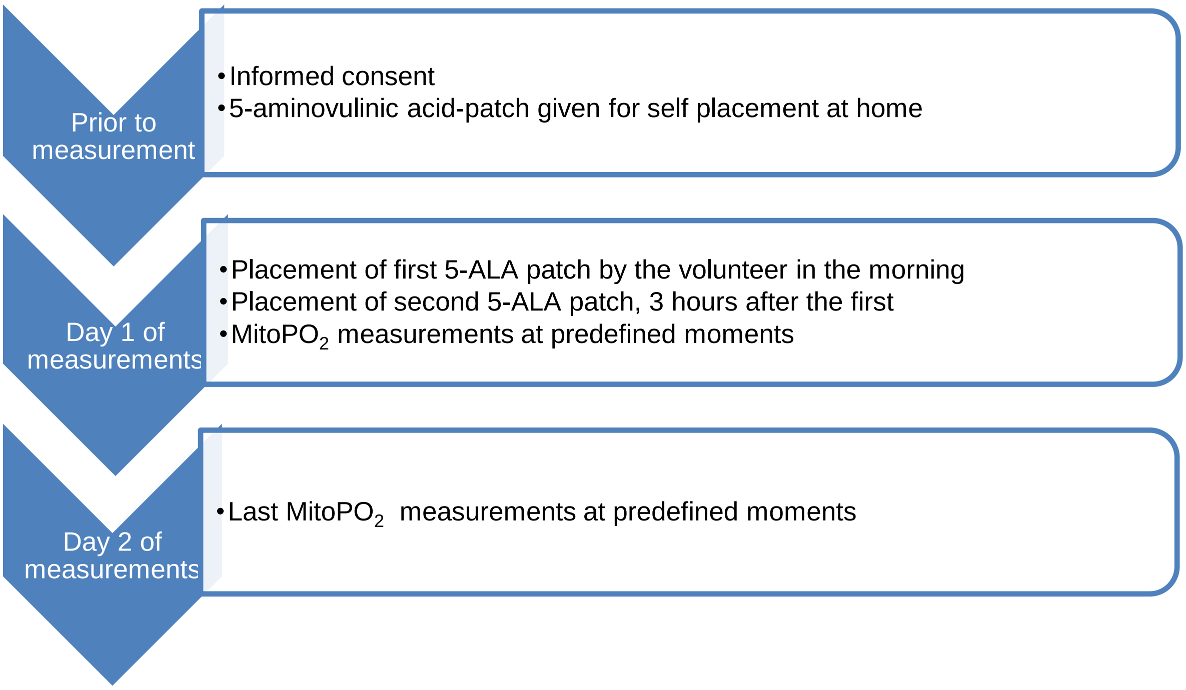

Figure 2: Gross overview of the clinical study design in neurosurgical patients

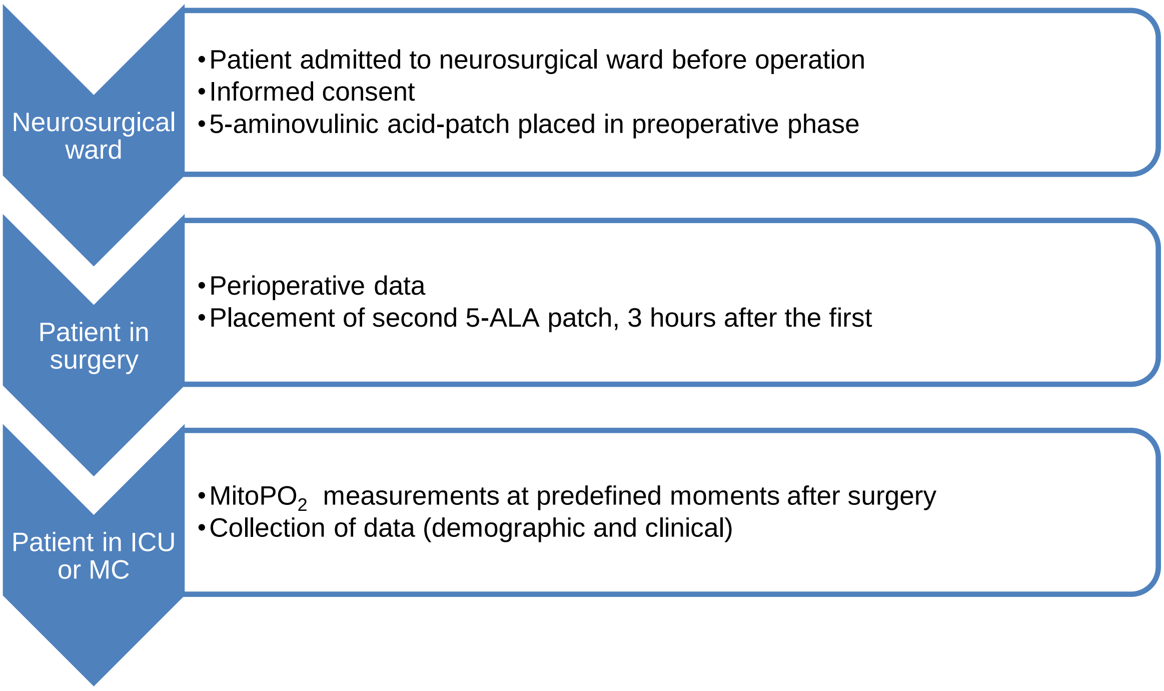

4. Study population

4.1 Population (base)

The study population will consist, initially, of healthy volunteers. This group was chosen to perform measurements on, to eliminate the effect of critical illness and interventions on the ICU and to better explore the effect of time-since-application of the ALA-patches. Neurosurgical ICU/MC patients will subsequently be included to further explore the effect of critical illness (ICU admission). Since this cohort of patients receives minimal interventions, they are ideal to limit potential affecters on the between- and therefore also the within-subject variability.

This study population will consist of patients undergoing elective neurosurgery with planned postoperative recovery of at least 24 hours in the ICU or MC. The LUMC is a neurosurgical center in which a wide variety of surgeries including tumor resection in the posterior cranial fossa (including vestibular schwannoma) are performed. These patients need to be monitored postoperatively due to the risk of bleeding, infections and the need for mechanical ventilation [15]. Clinical experience has shown that this cohort of patients are in general, hemodynamically the most stable patients and receive the least amount of interventions compared to other cohorts of patients in the ICU or MC. Furthermore, it is important to evaluate the COMET measurement system’s performance in the clinical setting. For these reasons, the cohort of elective neurosurgical patients would be ideal to investigate the reason of the increased between- and within-subject variability of mitoPO_2 in the ICU and MC setting.

4.2 Inclusion criteria

Patients meeting all these criteria will be included in the study:

- Age of patient is at least 18 years
- Healthy participants, defined as the absence of active or chronic disease (applicable only to healthy volunteer group).
- Patients are admitted to the ICU or MC after neurosurgery (applicable only to neurosurgery group).

4.3 Exclusion criteria

Patient meeting one of these criteria will be excluded from the study:

- patients without a legal representative in case the patient is not able to give informed consent
- pregnant or breast feeding women since there is no adequate data from the use of ALA in pregnant or breast feeding women[16]
- patients with porphyria and/or known photodermatosis
- patients with hypersensitivity to the active substance or to the plaster material of ALA
- insufficient comprehensibility of the Dutch language

4.4 Sample size calculation

The statistical significance level will be set at the 5% level. The sample size will be calculated to achieve a statistical power of 90%. In the pilot study preceding the INOX ICU 2 study, performed on 20 critically ill patients, the mean within-subject variability at the final timepoint (after 24 hours) was 8.7 mmHg with a standard deviation of 5.67 mmHg. In a stable ICU population, a within-subject variability greater than 4 mmHg will be deemed to be less precise. This is pragmatically chosen based on the initial course of within-subject variability calculated in the first 2 hours of measurements. Therefore, the sample size calculation would be as follows:

$Z_{\frac{\alpha}{2}} = 1.96 \text{ (two tailed for p} = 0.05)$

$Z_{1-\beta} = 1.28 \text{ (for } \beta = 0.10)$

$\mu = 8.7\ mmHg$

$\mu_0 = 4\ mmHg$

$\sigma = 5.67\ mmHg$

$n = \left(\sigma \frac{z_{\alpha/2} + z_{1-\beta}}{\mu - \mu_0}\right)^2$ = 15.

To allow for an expected 10% dropout rate the corrected sample size will be 15 x 1.1 = 17. The sample size will therefore be 17 subjects for both groups. The study will first commence in healthy volunteers. The study will continue in neurosurgical patients admitted to the ICU and MC of Leiden University Medical Center postoperatively. In 2018, on average 19 postoperative neurosurgical patients were admitted to the ICU / MC of the LUMC per month. For the period March 2019 – June 2019 (matching the period in which this study is expected to be executed) the average was 17.25 admissions per month. Taking these averages into account, 17 neurosurgical patients has been deemed feasible in the allotted time. Further information on the number of admissions of neurosurgical ICU/MC patients can be found in appendix 4.

5. Treatment of subjects

5.1 Investigational product: PpIX-TSLT with Comet

The COMET's non-invasive cutaneous mitoPO_2 measurements rely on the PpIX-TSLT (protoporphyrin IX-triple state lifetime technique). The PpIX-TSLT technique measures oxygen by oxygen-dependent quenching of delayed fluorescence lifetime of ALA (5-aminolevulinic acid)-induced mitochondrial PpIX. PpIX is the final precursor of haem in the haem biosynthesis pathway and is synthetized in the mitochondria. The conversion of PpIX to haem is the rate-limiting step and therefore causes a weak delayed fluorescence lifetime. Administration of exogenous ALA enhances PpIX to detectable levels and enhances mitochondrial origin of the delayed fluorescence signal.[6] Measurements are based on the detection of time of extinction of red light emitted by the tissue following excitation with green light (figure 2). This technique was first published in 2006.[17] The technique has been tested and calibrated for use in isolated organs and in vivo.[18-20] The PpIX-TSLT technique is described in detail by Mik (2013).[6]

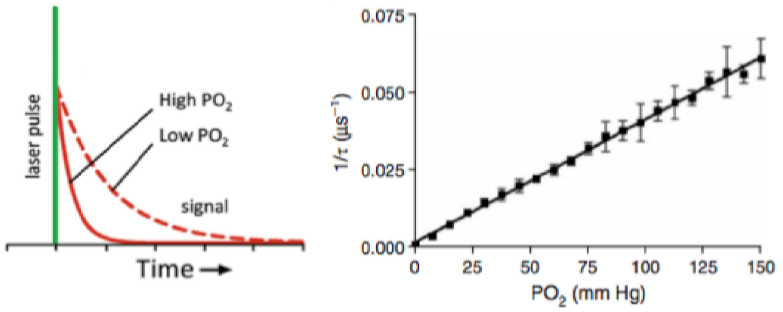

Figure 2: Detected delayed fluorescence. A short lifetime corresponds with high intra cellular oxygen concentration and low PO2 values correspond with a long lifetime (Mik, 2013). The lifetime can be translated to an oxygen tension with the help of a calibration

The COMET measurement system is an oxygen availability measurement tool that measures oxygen tension at the mitochondrial level by means of delayed fluorescence of protoporphyrin IX. The COMET monitor is used observationally. It will not be used to treat patients, nor is the information provided by the COMET used to alter patient treatment. The COMET measurement system is a non-invasive measuring system to determine cellular oxygen availability locally in human skin cells with a high concentration of protoporphyrin IX. It is CE marked as a medical device (DEKRA certificate number 2183975CE01, initially issued on 18 April 2016).

In this study, the mitoPO_2 of the skin will be measured. Reliable measurements can only take place with sufficient concentrations of protoporphyrin IX in the mitochondria. Therefore, a self-adhesive patch containing 8mg of ALA (Alacare®; Spirig AG, Egerkingen, Switzerland) will be placed on the anterior chest wall for induction of PpIX, for at least 4 hours before the first measurement can take place. To enhance ALA penetration, the skin will be cleaned with alcohol to remove the exposed layers of stratum corneum. Hair, if present, will be shaved.[21] Following at least 4 hours induction of PpIX, the ALA patch will be removed after the outline of the ALA patch is marked.

After placing the skin sensor (COMET Skin Sensor, Photonics Healthcare, the Netherland), calibration measurements will be made to ensure the quality of the measurements. Thereto, 5 measurements will be made in the first minute to establish a reliable and stable measurement. Occlusion of the microcirculation will be accomplished via local pressure with the measurement probe, which we use to validate the actual mitoPO_2 measurement. After these measurements, mitoPO_2 will be measured each minute for 5 minutes to obtain a mean mitoPO_2. To ensure that no other variables influence the measurements, measurement characteristics will be logged. In this log, the circumstances in which each measurement is made will be documented. Known intervening variables include, for example, skin temperature, thus skin temperature will be recorded. Other variables recorded in the log will be vasopressor use, dosage of the vasopressor, and nurse actions. Following mitoPO_2 measurement, the ALA patch will be re-used to protect the exposed skin for phototoxicity until the next measurement. After completion of the mitoPO_2 measurements the exposed skin will be protected from sunlight for 24 hours. An overview of the mitoPO2 measurement is given below.

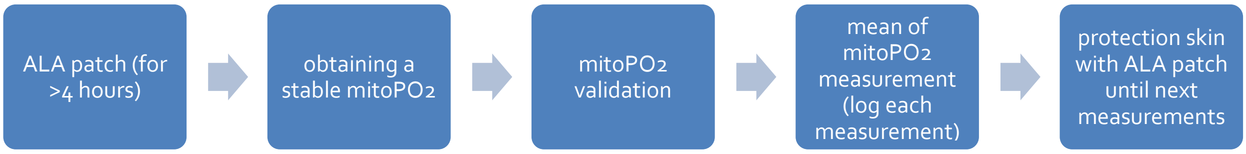

The skin sensor and other parts of the COMET will be cleaned according to cleaning guidelines for medical devices in the intensive care unit. In this case, the COMET will be cleaned with 70% ethanol. In the case of visible dirt, the COMET will be cleaned with water and soap prior to ethanol 70% application.

5.2 Use of co-intervention

Due to the risk of phototoxicity, it is important for the skin exposed to the ALA patch to be protected from sunlight during, between, and for 24 hours following measurements.

5.3 Escape medication

Should adverse effects take place, the skin would need to be protected from further damage by light exposure. Possible adverse effects include burning sensation of the skin, pruritis, hyperpigmentation, application site erythema, and local pain.[22 23] Pain killers could be administered, if local pain would persist despite these precautions.

6. Investigational product: the COMET

6.1 Name and description of medical device

Photonics Healthcare B.V. has developed an innovative non-invasive bedside monitoring system to measure Cellular Oxygen METabolism (the COMET). This device, for the first time, provides insight in the adequacy of oxygen supply and the actual oxygen demand. Available technologies only measure parameters in the blood, where oxygen is transported, but not where it is actually needed. The COMET measures availability and utilization of oxygen where oxygen is utilized: in the tissue cells.

The COMET measurement system consists of three parts:

1. The bedside COMET monitor: has a multi-touch screen that directly, in real time, displays the mitoPO2, the functional parameter of mitochondrial oxygen availability in mmHg. Additionally, signal quality and sensor temperature indication at the time of the most recent oxygen measurement are shown. The user can set the measurement interval and follow the reaction of cellular oxygen to various interventions, or perform a spot-check, a single measurement on a specific part of the skin of the situation at a particular time. A measurement series in a situation where pressure temporarily stops local blood flow can measure oxygen utilization. The device is a standalone unit that can be mounted on a moveable cart or used on a table.
2. The COMET Skin Sensor: is optically and electrically connected with the COMET measurement device. The optical fibers transmit optical signals for tissue excitation and fluorescence detection. The Skin Sensor creates a diverging excitation pulse allowing a superficial optical measurement of the oxygen in the mitochondria of the cells in the epidermis in a circular area of about 5 mm2. Fluorescence light from the tissue is collected and transmitted via the detection fiber towards the detector system. The sensor also detects ambient light and the temperature inside the sensor.
3. ALA patch and sensor fixation: The dermatologic agent ALA is applied to the patient skin. The sensor is fixated to the site with the help of a patch on which the skin sensor is placed. Simple ALA patches were developed by Photonamic and are marketed for photodynamic therapy, ALA crèmes are available from Galderma, photocure, DUSA and Biofrontera. This set up allows the physician to place the ALA-patch anywhere on the patient’s skin and, after four hours, place the sensor to locally determine the mitoPO2 level in the cells of the epidermis, which is then displayed on the monitor. Measurements can be performed every second for several minutes, or every few minutes for a period of 24 hours on the same spot of skin. After that, another location for the patch should be chosen.

6.1.1 The measurement specifications

Photonics Healthcare's COMET monitor measures absolute oxygen availability precisely and exactly where it is needed: in the mitochondria of the active cells. This provides doctors with quantitative insight in the availability and consumption of oxygen in their patients' cells and hence the need for a blood transfusion. To determine the oxygen tension in the mitochondria, the COMET measures the lifetime of the triplet state of PpIX, a precursor of haem and hence haemoglobin. The discovery that PpIX exhibits a 'glow-in-the-dark' effect with an oxygen-dependent life time was published in a Nature journal; patents are awarded in Europe and the US and exclusively licensed by Photonics Healthcare. The COMET probes the tissue with very short pulses of green light generated by a diode-pumped solid state laser. This light is brought to the site via an optical fibre. The COMET then observes the decay of the intensity of delayed fluorescence from the red spectrum of light collected from the tissue in the first milliseconds after the flash. Under normal conditions, PpIX is present in very low concentrations due to the presence of a negative feedback loop in which a product of PpIX, haem, acts to inhibit the production of ALA, a substrate for PpIX. This can be overcome by the exogenous administration of ALA, which on its turn leads to higher concentrations of protoporphyrin IX in the mitochondria.[24] ALA patches are already used in and approved for the therapy of benign skin disorders with photodynamic therapy. Its use is safe and provides good treatment results with excellent cosmetic outcome. The 2x2cm large Alacare patches (8mg) were developed by Photonamic and are marketed for the treatment of actinic keratosis, a benign skin disorder, with photodynamic therapy.

The key specifications of the COMET sensor system are:

- Detection range: 0 – 200 mmHg (arterial oxygen tension does not exceed 100 mmHg in normal physiologic situations).
- Tolerance of Monitor: +/- 15%, at least +/- 3 mmHg, for signal quality >75%, which is similar or better than many clinical measurement systems such as cardiac output (+/- 30%), tissue oxygen (+/- 30%) or haemoglobin (+/-20%).[25 26] Repeatability and stability of the measurement is high and absolute accuracy will improve as more information about the new measurement becomes known but already is higher than for some current parameters used in the clinic (for example cardiac output).
- Time to result: 1 second (for a prepared measurement location, i.e. placement of ALA-patch 4 hours previously to measurement).
- Simple handling (one hour of training is sufficient to train any doctoral student, nurse or doctor)
- All-in-one system, no need for other equipment or control mechanisms.

6.1.2 Current stage of development

The COMET measurement system has been developed based upon applicable regulations, standards (Medical device directive 93/42EEC, IEC 60601 rev 3, ISO 13485) and has been tested in preclinical tests and volunteer studies. The key milestones are described below.

The crucial step in the invention is the exploitation of the oxygen dependent optical properties of protoporphyrin IX. Because this substance is made in the mitochondria and can be measured on the skin, the problem of signal location, that has made all earlier attempts to directly measure cellular metabolism unsuccessful, is solved. First publication of the feasibility of the measurements was in 2006 in Nature Methods.[17]

Subsequently the method was tested in vivo on various organs,[27-29] validated against existing technologies[30 31] and it was shown that the measurement could be used to measure oxygen consumption i.e., cellular energy turnover in a first human volunteer.[6 11] Further studies demonstrated that cellular oxygen availability and consumption could safely be measured using ALA patches and COMET prototypes in a study in 30 human volunteers at Erasmus MC in Rotterdam.[21] It was also shown that information from the skin is representative for other parts of the body.[9] From September 2014 until April 2015, Van Diemen et al. measured the increase of cellular oxygen consumption with the COMET prototype in skin of 28 human volunteers after 4 weeks of daily statin use (Simvastatin, a cholesterol lowering drug suspected of modifying mitochondrial function) and its partial reversal by additional intake of ubiquinol (Q10), which contributes to mitochondrial function.[32] Next to the COMET the study also measured phosphocreatine recovery time after muscle exercise in 7-Tesla-31P-MR spectroscopy and mitochondrial membrane potential in white blood cells. The practically perfect correlation of the results in all groups is a strong validation of Photonics Healthcare's measurement against a very expensive and cumbersome technique.

Photonics Healthcare developed the COMET monitor, the first clinical monitor for cellular oxygen and conducted preclinical research with the help of partners, a Eurostars grant and investors.[33]

In patients receiving ALA assisted photodynamic therapy and during neurosurgery oxygen levels were measured with the COMET. These demonstrate that the COMET can be used in patients and measurements are stable.

6.2 Summary of known and potential risks and benefits

As described in the summary of product characteristics of Alacare, most known adverse and serious adverse reactions were reported when this product was used in combination with photodynamic therapy. Since patients in our study are exposed to a smaller light dose ($1mJ/cm^2$) than used in photodynamic therapy ($10J/cm^2$) and shorter duration of illumination, we expect less severe and less frequent adverse reactions as described in the summary of the product characteristics. Indeed, the study of Harms et al. acknowledges this by reporting minimal adverse reactions in their study with healthy volunteers.[21] Reported adverse reactions of ALA in combination with photodynamic therapy consisted of adverse reactions localised at the treatment site (local reactions) that were attributable to toxic effects of the photodynamic therapy (phototoxicity) in 99%

of patients. During application of Alacare and prior to photodynamic therapy illumination of the treatment site, 33% of patients show local reactions, most frequently pruritus, burning and erythema. During photodynamic therapy illumination, worsening of local reactions, namely erythema, burning and pain were reported most often. The symptoms are usually of mild or moderate severity and require early termination of illumination in 1% of the patients. Cooling of the treated area alleviated these symptoms. After therapy, pruritus, erythema, scabbing and exfoliation are the most frequent local reactions which are likewise mainly mild to moderate and persist for 1 to 2 weeks or occasionally longer. A common (< 10%) adverse reaction not involving the treatment site is headache. The incidence of adverse reactions in patients receiving Alacare plus photodynamic therapy illumination is shown in table 1.[16 34]

Table 1: Incidence of adverse reactions in patients receiving Alacare plasters in combination with photodynamic therapy

**Adverse reactions involving the treatment site (local reactions)**		
**General disorders and application site conditions**	Very common ≥ 1/10	Erythema, exfoliation, irritation, pain, pruritus, scabbing
	Common ≥ 1/100, < 1/10	Bleeding, desquamation, discharge, discomfort, erosion, hyper/hypopigmentation, oedema, reaction, swelling, vesicles
	Uncommon ≥ 1/1000, < 1/100	Burn, discolouration, excoriation, inflammation, ulcer
**Infections and infestations**	Common ≥ 1/100, < 1/10	Pustules
	Uncommon ≥ 1/1000, < 1/100	Infection
**Adverse reactions not involving the treatment site**		
**Nervous system disorders**	Common ≥ 1/100, < 1/10	Headache
**Infections and Infestations**	Uncommon ≥ 1/1000, < 1/100	Pyoderma
**Psychiatric disorders**	Uncommon ≥ 1/1000, < 1/100	Emotional distress
**Respiratory, thoracic and mediastinal disorders**	Uncommon ≥ 1/1000, < 1/100	Epistaxis
**Skin and subcutaneous tissue disorders**	Uncommon ≥ 1/1000, < 1/100	Skin discolouration
**Investigations**	Uncommon ≥ 1/1000, < 1/100	Alanine aminotransferase increased

In a feasibility study of mitoPO$_2$ measurement in 30 healthy volunteers, 45% of the volunteers had mild pruritus and/or erythema on the day of measurement, with only 2 volunteers experiencing transient hyperpigmentation of the skin due to premature exposure of the primed skin to sunlight [21]. No adverse reactions were seen in our INOX ICU-2 pilot study population (P16.303, results not

published yet). Due to the very low light dose (2 mJ/cm^2) and short duration (1 second) of illumination per measurement (on average 2 minutes per measurement moment) in comparison to conventional photodynamic therapy (10 J/cm^2 and a duration of 10 minutes on average), minimal adverse reactions are expected in our study population.

6.3 Description and justification of route of administration and dosage

In the current form, mitoPO_2 measurements need administration of ALA to induce enough mitochondrial PpIX for detection of the delayed fluorescence signal. ALA is a precursor in porphyrin synthesis, and its application induces the accumulation of PpIX inside mitochondria. ALA is clinically used in photodynamic diagnosis and therapy of cancer. While systemic administration of ALA in itself is safe, the transient photosensitization of the skin requires patients to limit exposure to (sun) light in the days after treatment. Obviously, this poses constraints to the use of systemically administered ALA in the general population. As an alternative to systemic administration, ALA can be topically applied. Mik et al. have already demonstrated that topical administration of ALA cream to the skin induces oxygen-dependent delayed fluorescence in rats and humans.[6]

6.4 Method of administration

Previous research with this technique has shown that ALA patches are a safe and reliable method to induce PpIX. Therefore, ALA patches are used in this study to enhance PpIX for the mitoPO_2 measurements. [21]

6.5 Preparation and labelling of medical device

In accordance with the Dutch ‘Kwaliteitswet zorginstellingen’, the medical device used for this study has to be controlled for multiple aspects like safety and quality before it can be used in the hospital. This is done by a department in the hospital, called health technology. The COMET has been through this department and has been approved. It received a label of approval from Leiden University Medical Centre.

7. Methods

7.1 Study procedures

7.1.1 General

The study population will initially consist of healthy volunteers recruited via LUMC notice boards. Subjects will be interviewed prior to participation by a member of the research team, at which time information about the study will be provided and eligibility for participation will be determined based on the inclusion and exclusion criteria. Due to the ‘off-label’ use of the Alacare patches, specific information pertaining to its current use will be given. A written informed consent will be obtained from all volunteers. Upon inclusion further instructions for ALA-patch application will be provided along with alcohol wipes and 1 ALA-patch.

A self-adhesive ALA-patch will be applied parasternal and a second ALA-patch 3 hours later to rule out influence of the ALA-patch on the measurement variability, while keeping all other possible influencing variables the same. This offset of 3 hours was chosen since an increase in between- and within-subject variability was observed after 3 hours in the INOX ICU-2 pilot study. The course of mitoPO_2 measurements will be measured at multiple predefined moments with several overlapping measurements. Subsequently, mitoPO_2 measurements will be performed in neurosurgical ICU/MC at multiple predefined moments. This information will be used to further explore the increased variability in the critical care setting where it was first observed.

The study will include patients undergoing elective neurosurgery with postoperative ICU or MC observation and recovery. Patients are typically admitted to the neurosurgical ward 1 day prior to operation. On that day an information leaflet will be given and informed consent will be sought. Patients or their legal representatives will be told that a person can only participate in the study if he/she meets the inclusion criteria. If written informed consent is given, on the day of the operation, the self-adhesive ALA-patch will be placed parasternal in the operating room in the preoperative phase. Three hours after placement of the first ALA-patch, the second offset ALA-patch will be placed either at the ICU/MC or perioperatively in coordination with the anaesthesiology team. This will be done by a member of the research team. The induction time of PpIX is at least 4 hours, therefore the first measurements will be taken postoperatively. MitoPO_2 measurements will follow the same schedule used in the volunteer group. After inclusion of a patient meeting the eligibility criteria, each patient will be assigned a unique study number.

7.1.1 Clinical Care

For the neurosurgical group patient management will take place according to usual care. When patients meet the inclusion criteria, an ALA-patch will be placed in the preoperative phase and 3 hours later. After at least 4 hours of PpIX induction, the cutaneous mitoPO2 sensor will be placed to make the first measurements after which measurements of the mitoPO2 will be made at predefined moments.

7.2 Data collection

While waiting for the induction of PpIX after placement of the ALA patch, baseline data will be gathered. Appendix 2 shows an overview of the data collection. First of all, demographic data will be collected: age in years, sex, weight, height, Body Mass Index, allergies, medication use, comorbidities (acute and chronic), and type of surgery. Also, APACHE (Acute Physiology and Chronic Health Evaluation)-II and APACHE-IV will be assessed. The demographic data can be obtained from the hospital's electronic patient dossier system (EPD). Information regarding perioperative characteristics will collected as well, including the length of operation, type of anaesthetics used and perioperative complications.

Clinical data will be assessed pre- as well as intra- and postoperatively including heart frequency (HF), blood pressure (BP), and peripheral measured oxygen saturation (SpO_2). Postoperatively, these will be assessed at the same time points as the $mitoPO_2$ measurements. Other data, if applicable, collected from the EPD are: vasopressor use, inotropic therapy, mechanical ventilation duration and settings, PaO_2/fractional inspired oxygen (FiO_2) ratio, and Alveolar-arterial gradient. Interventions given to patients in the ICU or MC, such as intravenous fluid therapy, transfusion, medication and/or procedures, will be noted. No extra samples will be taken from patients. Appendix 2, figure 2 gives an overview of measurements part of standard care, and which of those measurements are part of the study.

At certain time points, described in detail in the next section, $mitoPO_2$ measurements will be done. Signal quality will be measured using the COMET monitor device itself, where a higher signal quality corresponds with a stronger PpIX signal and a more sensitive $mitoPO_2$ measurement. In a previous study it was shown that for PpIX-based delayed fluorescence measurements a signal-to-noise ratio > 20 limits the noise-induced potential error in $mitoPO_2$ measurements below 2% over the physiological PO_2 range [30]. Signal-to-noise ratio (SNR) in time domain lifetime measurements (as implemented in the COMET) is commonly defined as the ratio of maximal signal amplitude (at the start of the decay) to the maximum signal of the noise (peak-to-peak). Since SNR was not regarded to be an intuitive measure for the end user of a COMET device, the software converts SNR to signal quality using a non-linear relationship in which e.g. a SNR of 100 provides a signal quality of almost 80%. In a previous study, it was shown that for PpIX-based delayed fluorescence measurements, a signal-to-noise ratio > 20 limits the noise-induced potential error in $mitoPO_2$ measurements below 2% over the physiological PO_2 range. [30] A signal quality of 25% corresponds to a SNR of 20, can easily be obtained in practice and keeps the noise-induced uncertainty in the measurement to an acceptable low level of less than 2%. [30] This is why we choose to (arbitrarily) define 25% as the minimum required signal quality for a feasible measurement. Between-subject variability is defined as the variation of $mitoPO_2$ at each measurement time point in the study population. Therefore, mean $mitoPO_2$ and standard deviation (SD) is calculated per measurement time point. This SD represents the between-subject variability. Within-subject variability is defined as the variation of $mitoPO_2$ within one subject at each measurement time point, since multiple measurements are taken at each timepoint. Since a mean $mitoPO_2$ at each timepoint is used, the

standard deviation of this measurement per participant is calculated. The mean of this SD represents the within-subject variability of the study population.

All data will be entered after validation in a study database for subsequent tabulation and statistical analysis. The data will be handled confidentially and anonymously. Patients re-admitted to the ICU or MC and already participating in this study will only be followed according to the study protocol. Thus re-admitted patients will not be included in the study for the second time.

7.2.1 Sample handling and Measurements:

Figure 4: MitoPO2 measurement with the COMET

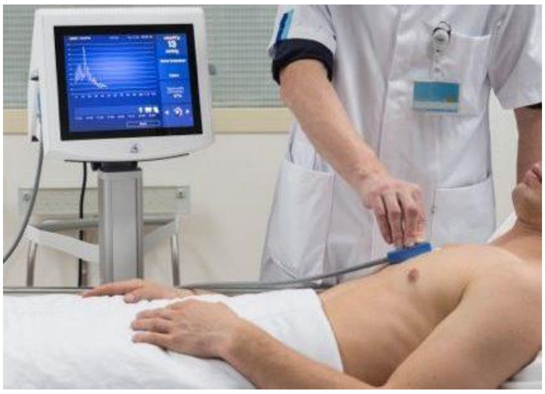

In order to further investigate the between- and within-subject variability of $mitoPO_2$ over time, $mitoPO_2$ measurements will be performed over a 24-27 hour period per patch.

For the first patch these moments are:
- 4 hours after ALA-induction (Directly after surgery in patient group) (=T0)
- 1 hour after T0 (=T1)
- 3 hours after T0 (=T2)
- 6 hours after T0 (=T3)
- 24 hours after T0 (=T5)
- 27 hours after T0 (=T6)

Patch 2 is applied 3 hours after the first patch, with measurement moments corresponding to patch 1 with overlap (see table 2):
- 4 hours after ALA-induction (=T0)
- 1 hour after T0 (=T1)
- 3 hours after T0 (=T2)
- 21 hours after T0 (=T4)
- 24 hours after T0 (=T5)

Table 2: Overview of measurement moments for each patch (shading indicates overlapping moments).

Patch #	Measurement	Hours after 4 hours ALA-induction	Comments
1	T0	0	Directly postoperatively (in patient group)
1	T1	1	
1	T2	3	
2	T0	0	3 hour offset relative to patch 1
2	T1	1	
2	T2	3	
1	T3	6	

1	T5	24	
2	T4	21	
1	T6	27	
2	T5	24	

7.3 Withdrawal of individual subjects

Subjects can leave the study at any time for any reason if they wish to do so without any consequences. The investigator can decide to withdraw a subject from the study for urgent medical reasons. These subjects will not be subjected to follow-up.

8. Safety reporting

8.1 Section 10 WMO event

In accordance to section 10, subsection 4, of the WMO, the coordinating investigator will suspend the study if there is sufficient ground that continuation of the study will jeopardise subject health or safety more than was foreseen in the research proposal. The coordinating investigator will notify the accredited METC without undue delay of a temporary halt including the reason for such an action. The study will be suspended pending a further positive decision by the accredited METC. The investigator will take care that all subjects are kept informed.

8.2 Possible (serious) adverse events and suspected unexpected serious adverse reactions

The ALA patches are already used in and approved for the therapy of benign skin disorders with photodynamic therapy . Its use is safe and provides good treatment results with excellent cosmetic outcome. The 2x2cm large Alacare patches (8mg) were developed by Photonamic and are marketed for the treatment of actinic keratosis, a benign skin disorder, with photodynamic therapy. In dermatology, its use has been investigated.[6] In phototherapy, the treatment effect depends on the production of singlet oxygen by photoactivation of PpIX. Excitation of PpIX induces apoptosis in cells as a result of oxygen-radical formation. In general, this requires illumination with continuous light and a high cumulative light dose (10 J/cm^2). In contrast, PpIX-TSLT uses short-pulsed excitation and a total light dosage, i.e., orders of magnitude less than used for photodynamic therapy (1 mJ/cm^2).[6] A recent systematic review showed no significant (serious) adverse effects of ALA patches.[35]

Side effects of ALA described with photodynamic therapy are burning of the skin (<50%, resolved in 1-4 days), burning sensation of the skin (<92%), pruritus, hyperpigmentation (22-36%), edematous lesion (35%), application site erythema (92-99%), local pain (1-92%, <30% severe), application site irritation (72%), local haemorrhage(1-4%), and headache (<10%). Most of these reactions resolved within 2 weeks. There are no reported (serious) adverse events.[16 22 23] Since the light load at the cutaneous-PpIX-TSLT is much lower than in phototherapy, no adverse events are expected. While PpIX-TSLT is likely to be safe, the effects of phototoxicity after PpIX induction should always be considered a potential risk requiring a risk and safety assessment in any intended application.[6]

8.3 Adverse events (AEs)

Adverse events are defined as any undesirable experience occurring to a subject during the study, whether or not considered related to the investigational product. All adverse events reported spontaneously by the subject or observed by the investigator or his staff will be recorded.

8.4 Serious adverse events (SAEs)

A serious adverse event is any untoward medical occurrence or effect that

- results in death;
- is life threatening (at the time of the event);
- requires hospitalisation or prolongation of existing inpatients' hospitalisation;
- results in persistent or significant disability or incapacity;
- Any other important medical event that did not result in any of the outcomes listed above due to medical or surgical intervention but could have, based upon appropriate judgement by the investigator.

An elective hospital admission will not be considered as a serious adverse event.

The investigator will report all SAEs to the sponsor without undue delay after obtaining knowledge of the events. Patients in the ICU and MC are critically ill and therefore more prone to SAEs. ICU mortality in the Netherlands is approximately 8.4%. Thirty-five percent of the patients are in need for vasopressor therapy, and 46% of the patients are mechanically ventilated. Furthermore, the National Intensive Care Evaluation Report of 2018 reports that the mean ICU admittance duration is 3 days with a mean in hospital admittance duration of 13 days. Around 75% of the total ICU patients have a low APACHE IV score, and therefore a mortality chance of less than 30%.[36] Thus, SAEs in relation to underlying disease and expected SAEs during the ICU course will not be reported.

The coordinating investigator will report the SAEs through the web portal *ToetsingOnline* to the accredited METC that approved the protocol, within 7 days of first knowledge for SAEs that result in death or are life threatening followed by a period of maximum of 8 days to complete the initial preliminary report. All other SAEs will be reported within a period of maximum 15 days after the sponsor has first knowledge of the serious adverse events.

8.5 Suspected unexpected serious adverse reactions (SUSARs)

Adverse reactions are all untoward and unintended responses to an investigational product (in this case the ALA patch as part of the mitoPO_2 measurement) related to any dose administered. No SUSARs of the ALA patch are reported. Therefore, no SUSARs are expected during this study.

Unexpected adverse reactions are SUSARs if the following three conditions are met:

1. the event must be serious (see chapter 7.4);
2. there must be a certain degree of probability that the event is a harmful and an undesirable reaction to the ALA patch, regardless of the administered dose;
3. the adverse reaction must be unexpected, that is to say, the nature and severity of the adverse reaction are not in agreement with the product information as recorded in:
   - Summary of Product Characteristics (SPC) for the COMET device;

- Investigator's Brochure for the ALA patch.

The coordinating investigator of LUMC will report expedited the following SUSARs through the web portal *ToetsingOnline* to the METC:

- SUSARs that have arisen in the clinical study that was assessed by the METC;
- SUSARs that have arisen in other clinical studies of the same sponsor and with the same medicinal product, and that could have consequences for the safety of the subjects involved in the clinical study that was assessed by the METC.

The remaining SUSARs are recorded in an overview list (line-listing) that will be submitted once every half year to the METC. This line-listing provides an overview of all SUSARs from the ALA patch, accompanied by a brief report highlighting the main points of concern.
The expedited reporting of SUSARs through the web portal *ToetsingOnline* is sufficient as notification to the competent authority.

The expedited reporting will occur not later than 15 days after the sponsor has first knowledge of the adverse reactions. For fatal or life threatening cases the term will be maximal 7 days for a preliminary report with another 8 days for completion of the report.

The coordinating investigator of LUMC is responsible for documentation of the SUSAR in the report and ToetsingOnline.nl. The principal investigators in the participating centres will inform the coordinating investigator in <24 hours after the occurrence of the SUSAR. In the case of occurrence of 2 or more SUSARs in 6 months' time, the study team will discuss possible termination of the study.

8.6 Annual safety report

In addition to the expedited reporting of SUSARs, the coordinating investigator will submit, once a year throughout the clinical study, a safety report to the accredited METC. This will be combined with the annual progress report.
This safety report consists of:

- a list of all suspected (unexpected or expected) serious adverse reactions, along with an aggregated summary table of all reported serious adverse reactions, ordered by organ system.
- a report concerning the safety of the subjects, consisting of a complete safety analysis and an evaluation of the balance between the efficacy and the harmfulness of the ALA patch.

8.7 Follow-up of adverse events

All AEs will be followed until they have abated, or until a stable situation has been reached. Depending on the event, follow up may require additional tests or medical procedures as indicated,

and/or referral to the general physician or a medical specialist. SAEs need to be reported until end of study within the Netherlands, as defined in the protocol

8.8 Data Safety Monitoring Board

This is a moderate risk study in which the side effect of ALA are well described. The side effects are based on studies with photodynamic therapy. Since the total lightload in photodynamic therapy is significantly higher and longer than during the measurements in this study, no SAEs and SUSARs are expected.[6 9] Indeed, studies using the ALA plaster for mitoPO2 measurement in Rotterdam reported no serious adverse events and no SUSARs.[21 37 38] These studies were performed on healthy volunteers,[21] surgical patients[37] and patients with chronic anaemia.[39] However, these studies reported local side effects of the ALA plaster consisting of erythema, pruritus and transient hyperpigmentation. All these effects were temporary and disappeared mostly within 1-2 weeks' time. No local side effects or other adverse events were seen in our pilot study. To ensure the safety of our study participants, monitoring will take place during the study. Furthermore, the in- and exclusion criteria are optimised to minimize the risk for participants. Since safety is an important of our study, we will have attention for AEs, SAEs and SUSARs. With an independent expert on data safety monitoring board confirming the needlessness of a DSMB for the INOX ICU-2 study, no DSMB was initiated for this study either.

9. Statistical analysis

9.1 Primary study outcome

The primary endpoint is the between- and within-subject variability of mitoPO_2 measurements using the COMET measurement system at various moments during a 24 hour period with offset ALA-patches in healthy volunteers and neurosurgical ICU/MC patients to describe the variance mitoPO_2 measurements and the effect of time-since-application of the ALA-patch and critical care interventions. Between-subject variability is defined as the variation of mitoPO_2 at each measurement time point over the whole group. A mean of the mitoPO2 measurements and the standard deviation (SD) is calculated. This SD represents the between-subject variability. Within-subject variability is defined as the variation of mitoPO_2 within one subject at each measurement time point. At each timepoint multiple measurements are taken. Since a mean mitoPO_2 at each timepoint is used, the standard deviation (SD) of this measurement per participant is calculated. The mean of this SD of the study population represents the within-subject variability. These will be taken separately per ALA-patch.

9.2 Secondary study outcomes

Secondary study outcomes include:

- Description of between- and within-subject variability in healthy volunteers and neurosurgical patients in relation to the between- and within-subject variability of the INOX ICU-2 pilot study (P16.303)
- Report on adverse and serious adverse events of the mitoPO_2 measurements.

9.3 Statistical methods to be employed

Descriptive statistics will be used to describe the characteristics of the study population. The mitoPO_2 at the different time points will be described with means, standard deviations (SD), medians and interquartile ranges (IQR) (Q1/Q3) as appropriate. For each time point, for each patient, a mean of 5 measurements of mitoPO_2 is calculated with a corresponding SD. The mean of these standard deviations will be used to calculate the within-subject variability and the standard deviation of the overall mean per time-point will be used to calculate the between-subject variability.

Normality of variable distribution will be inspected using histograms. Assuming normal distribution of variables, the p values of a paired samples t-test will be reported for comparing overlapping measurement moments between patch 1 and patch 2. If variables are not normally distributed a Wilcoxon signed ranked test will be performed.

Signal quality of the mitoPO_2 measurement will be described using the mean and SD of the signal quality. The statistical analysis will be conducted using the SPSS (Statistical Package for the Social Sciences) statistical package, release 23.0 (SPSS Inc., Chicago, IL) and STATA.

10. Ethical considerations

10.1 Regulation Statement

The study will be conducted according to the principles of the Declaration of Helsinki (accepted by the WMA General Assembly, October 2013, Fortaleza, Brazil) and in accordance with the Medical Research Involving Human Subjects Act (WMO).

10.2 Recruitment and Consent

The protocol of this study will be submitted to the Medical Ethics Committee of the Leiden- Den Haag-Delft. The study will not commence before formal approval has been granted.

The study population consists of patients undergoing elective surgery. Patients admitted to the neurosurgical department which will be admitted to the ICU or MC post-operatively, will be asked for an informed consent on the day before the neurosurgical operation. An information leaflet will be given and informed consent will be sought. Patients or their legal representatives will be told that a person can only participate in the study if he/she meets the inclusion criteria. If written informed consent is given the self-adhesive ALA-patch will be placed in the preoperative phase. The induction time of PpIX is at least 4 hours, therefore the first measurements will be taken postoperatively. After inclusion of a patient meeting the eligibility criteria, each patient will be assigned a unique study number.

10.3 Objection by minors or incapacitated subjects

Section 4, subsection 2, of the WMO stipulates that a legally incompetent adult cannot be forced to undergo a treatment or behave in a particular manner in the context of non-therapeutic research against his or her will. Subjects younger than 18 years are already excluded from the study. We don’t expect patients that are not capable for the informed consent procedure. If there are adults, that are incapable of giving informed consent, a legal representative will be asked to give informed consent according to the GCP guideline. When the subject is able to give informed consent after being included in the study by consent of the legal representative and denies any further participation, the subject will be excluded from the study without any consequences. The data collected up to that point will be used for analysis. No additional data will be collected.

10.4 Benefits and risk assessment, group relatedness

The proposed study aims to describe the between- and within-subject variability of mitoPO_2 measurements. The measurements necessary to assess the defined study endpoints are not expected to negatively influence the result of treatment.

10.5 Compensation for injury

The Leiden University Medical Centre has a liability insurance which is in accordance with article 7, subsection 6 of the WMO.

The LUMC (also) has an insurance which is in accordance with the legal requirements in the Netherlands (Article 7 WMO and the Measure regarding Compulsory Insurance for Clinical Research in Humans of 23th June 2003). This insurance provides cover for damage to research subjects through injury or death caused by the study.

1. € 650.000, -- (six hundred and fifty thousand Euro) for death or injury for each subject who participates in the Research;
2. € 5.000.000, -- (five million Euro) for death or injury for all subjects who participate in the Research;
3. € 7.500.000, -- (seven million and five hundred thousand Euro) for the total damage incurred by the organization for all damage disclosed by scientific research for the Sponsor as 'verrichter' in the meaning of said Act in each year of insurance coverage.

The insurance applies to the damage that becomes apparent during the study or within 4 years after the end of the study.

11. Administrative Aspects and Publication

11.1 Handling and storage of data and documents

All patients will be addressed to with a random patient identification code, which will be generated by CASTOR EDC, the electronic case report form. The codebook will be stored digitally, outside the investigator site file folder, with restricted access. Only the research team, monitor, and IGJ will have access to this folder. The code will be safeguarded by the coordinating investigator. The codebook will be encrypted with a password. All data will be stored for further publication. All handling of personal data will comply with the EU General Data Protection Regulation (GDPR) and the Dutch Act on Implementation of the General Data Protection Regulation (in Dutch: Uitvoeringswet AVG). Data will be stored for 20 years at the research location. Source data on the COMET device will be anonymous, it only contains date and time of measurement, it is not possible to enter patient name, personal number or date of birth. The COMET can only retrieve the data of the current/most recent measurement series until the user selects a new series or measurement spot. To store COMET data, it will be downloaded via an USB memory onto a computer memory conforming to the institution's privacy guidelines and the USB memory will be erased. The COMET data will be shared anonymously with the manufacturer.

11.2 Monitoring and Quality assurance

The risks associated with this study are moderate for the subjects (more information can be found in appendix 3). Therefore, only on-site monitoring is needed, which will happen in accordance of the NFU guideline of monitoring. Being labelled as a moderate risk study, the monitoring will be moderate to ensure the integrity and safety of the study participants. The monitor pool of Leiden University Medical Center, which are qualified for monitoring, will monitor this study. The monitor pool is an independent group of data managers or research nurses, who aren't involved in the study in any way other than monitoring. They will report to the principal investigator and to the coordinating investigator. The head of department will be notified by the monitor, when the monitor notices frequent or substantial omissions.

11.3 Amendments

Amendments are changes made to the research after a favourable opinion by the accredited METC has been given. All amendments will be notified to the METC that gave a favourable opinion. A 'substantial amendment' is defined as an amendment to the terms of the METC application, or to the protocol or any other supporting documentation, that is likely to affect to a significant degree:

- the safety or physical or mental integrity of the subjects of the trial;
- the scientific value of the trial;
- the conduct or management of the trial; or
- the quality or safety of any intervention used in the trial.

All substantial amendments will be notified to the METC and to the competent authority. Non-substantial amendments will not be notified to the accredited METC and the competent authority, but will be recorded and filed by the sponsor.

11.4 Annual progress report

The investigator will submit a summary of the progress of the trial to the accredited METC once a year. Information will be provided on the date of inclusion of the first subject, numbers of subjects included and numbers of subjects that have completed the trial, unexpected problems and amendments.

11.5 End of study report

The investigator will notify the accredited METC of the end of the study within a period of 8 weeks. The end of the study is defined as the 90th day after the last patients' admittance to the ICU. In case the study is ended prematurely, the investigator will notify the accredited METC within 15 days, including the reasons for the premature termination. Within one year after the end of the study, the investigator/sponsor will submit a final study report with the results of the study, including any publications/abstracts of the study, to the accredited METC.

11.6 Public disclosure and publication policy

The study protocol and analysis plan will be published before start of the study on clinicaltrials.gov. The results of the study will find their way into (inter–) national scientific journals and guidelines.

12. Structured risk analysis

In the following analysis we distinguish potential issues of concern related to the mitoPO_2 measurements

a. Level of knowledge about mechanism of action

The mechanism of action of mitochondrial oxygen tension measurements is well investigated since 2006 and published in several articles.[6 17 18 27-29] Compared with other techniques to measure oxygen at the tissue level, oxygen-dependent quenching of delayed fluorescence of PpIX has some distinct advantages. It is quantitative, and, unlike oxygen electrodes, once calibrated it does not need recalibration at the time of usage. As opposed to oxygen saturation measurements based on near-infrared spectroscopy, the measurement site within the tissue is well defined. Therefore, the signal is not sensitive to changes in vessel density, like capillary recruitment. Furthermore, the technique relies on lifetime measurements instead of intensity measurements and therefore is highly insensitive to changes in tissue optical properties occurring, e.g., in case of venous congestion.[6]

b. Previous exposure of human beings with the test product(s) and/or products with a similar biological mechanism

Topical application of ALA is registered for use of photodynamic therapy of skin lesions. Its use for measuring mitoPO_2 in healthy volunteers has been proven to be safe in the finished study with protocol number NL37911.078.11 in which the PpIX-TSLT technique was tested on 30 healthy volunteers. All healthy volunteers experienced the skin preparation and measurements as non-problematic. Due to either the skin preparation or the ALA patch, 45% of the volunteers suffered from mild pruritus and/or erythema on the actual measurement day; these minor complaints were no longer present the day after the measurements. Only two volunteers had transient hyperpigmentation of the skin after the measurements, possibly due to premature exposure of the primed area to sunlight (against our advice). The hyperpigmentation was temporary and disappeared within one month. None of the volunteers sustained long-term skin damage, as established one month after the experiments.[21]

ALA was also used in finished study with protocol number NL56686.091.16 in May and June 2016 at UMC St. Radboud in Nijmegen. This study induced endotoxaemia in human volunteers. Among other measurements, the COMET measurement system and Alacare were used to measure oxygen availability and consumption. ALA was applied on the evening before the endotoxaemia day. The study again confirmed the safety profile of the COMET's measurement also during endotoxaemia. The measurements of the COMET identified change of mitochondrial function in vivo during human endotoxemia.[38]

Other protocols use the same measuring technique: The running studies with Protocol nr: NL51187.078.14 (Photodynamic therapy study) and Protocol nr: NL51937.078.15 (Non-invasive monitoring of mitochondrial oxygen consumption and oxygenation (COMET): observational clinical study) and NL55664.078.15 (MOTIFATE pilot, a study of blood transfusion and fluid administration in chronic anaemia).

c. Can the primary or secondary mechanism be induced in animals and/or in ex-vivo human cell material?

Mitochondrial oxygen tension research has been performed in cells and animals prior to this research proposal.[9-12 29 31] PpIX-TSLT is one of the central techniques used in translational research in the Laboratory of Experimental Anaesthesiology of Erasmus Medical Center.

d. Selectivity of the mechanism to target tissue in animals and/or human beings

Not applicable.

e. Analysis of potential effect

The PpIX-TSLT technique as well as the COMET have been investigated and have been proven safe, as demonstrated by studies on volunteers and patients. The possible effect of phototoxicity after PpIX induction is a potential risk, but the PpIX-TSLT uses short-pulsed excitation and a fraction of the total light dosage used in photodynamic therapy. This risk is therefore considered to be very low.[17 34] We expect no effects of the measurement for the patients (aside from reported temporary mild discomfort like erythema and pruritus associated with the topical application of ALA). During the pilot study of the INOX ICU-2 study, no side effects besides mild pruritis in 1 patient was seen, confirming the expected low risk.

f. Pharmacokinetic considerations

Not applicable.

g. Study population

Postoperative patients in the ICU are vulnerable patients. According to GCP guidelines, a non-therapeutical study can only take place if it has a likelihood of benefit for group represented by the subject. With the results of this study potential factors affecting the variance of the mitoPO_2 measurements could be better understood, and applied, ranging from postsurgical to critical ill patients to reduce measurement error, and therefore assist us in the interpretation of the mitoPO_2 measurements. The effect of time-since-application of the ALA-patch will also be studied in a cohort of healthy volunteers. This study entails low risk and burden for the subjects since it is a non-invasive measurement with no known serious adverse reactions.

h. Interaction with other products

None expected, see Alacare product information.[16]

i. Predictability of effect

No effect of the measurements is expected. Minor temporary skin lesions can occur, as seen in the healthy volunteers. With the COMET we expect less effect, due to progressive technique since the study in healthy volunteers, so the light intensity and total light dosage will be even less.

j. Can effects be managed?

No effects of the mitoPO_2 measurements are expected, but when there is any indication of a skin lesion (e.g. unexpected pain during the measurements), the measurements can and will be stopped.

13. References

1. Bronicki RA. Hemodynamic Monitoring. *Pediatr Crit Care Med* 2016;17(8 Suppl 1):S207-14. doi: 10.1097/PCC.0000000000000779 [published Online First: 2016/08/05]
2. Biedrzycka A, Lango R. Tissue oximetry in anaesthesia and intensive care. *Anaesthesiol Intensive Ther* 2016;48(1):41-8. doi: 10.5603/AIT.2016.0005 [published Online First: 2016/03/12]
3. Bickler P, Feiner J, Rollins M, et al. Tissue Oximetry and Clinical Outcomes. *Anesth Analg* 2017;124(1):72-82. doi: 10.1213/ANE.0000000000001348 [published Online First: 2016/06/17]
4. Springett R, Swartz HM. Measurements of oxygen in vivo: overview and perspectives on methods to measure oxygen within cells and tissues. *Antioxid Redox Signal* 2007;9(8):1295-301. doi: 10.1089/ars.2007.1620 [published Online First: 2007/06/20]
5. Vincent JL, Rhodes A, Perel A, et al. Clinical review: Update on hemodynamic monitoring--a consensus of 16. *Crit Care* 2011;15(4):229. doi: 10.1186/cc10291 [published Online First: 2011/09/03]
6. Mik EG. Special article: measuring mitochondrial oxygen tension: from basic principles to application in humans. *Anesth Analg* 2013;117(4):834-46. doi: 10.1213/ANE.0b013e31828f29da [published Online First: 2013/04/18]
7. Konrad FM, Mik EG, Bodmer SI, et al. Acute normovolemic hemodilution in the pig is associated with renal tissue edema, impaired renal microvascular oxygenation, and functional loss. *Anesthesiology* 2013;119(2):256-69. doi: 10.1097/ALN.0b013e31829bd9bc [published Online First: 2013/07/11]
8. Balestra GM, Mik EG, Eerbeek O, et al. Increased in vivo mitochondrial oxygenation with right ventricular failure induced by pulmonary arterial hypertension: mitochondrial inhibition as driver of cardiac failure? *Respir Res* 2015;16:6. doi: 10.1186/s12931-015-0178-6 [published Online First: 2015/02/04]
9. Harms FA, Bodmer SI, Raat NJ, et al. Cutaneous mitochondrial respirometry: non-invasive monitoring of mitochondrial function. *J Clin Monit Comput* 2015;29(4):509-19. doi: 10.1007/s10877-014-9628-9 [published Online First: 2014/11/13]
10. Harms FA, Bodmer SI, Raat NJ, et al. Non-invasive monitoring of mitochondrial oxygenation and respiration in critical illness using a novel technique. *Crit Care* 2015;19:343. doi: 10.1186/s13054-015-1056-9 [published Online First: 2015/09/24]
11. Harms FA, Voorbeijtel WJ, Bodmer SI, et al. Cutaneous respirometry by dynamic measurement of mitochondrial oxygen tension for monitoring mitochondrial function in vivo. *Mitochondrion* 2013;13(5):507-14. doi: 10.1016/j.mito.2012.10.005 [published Online First: 2012/10/16]
12. Romers LH, Bakker C, Dollee N, et al. Cutaneous Mitochondrial PO2, but Not Tissue Oxygen Saturation, Is an Early Indicator of the Physiologic Limit of Hemodilution in the Pig. *Anesthesiology* 2016;125(1):124-32. doi: 10.1097/ALN.0000000000001156 [published Online First: 2016/05/14]
13. Kelty CJ, Brown NJ, Reed MW, et al. The use of 5-aminolaevulinic acid as a photosensitiser in photodynamic therapy and photodiagnosis. *Photochem Photobiol Sci* 2002;1(3):158-68. [published Online First: 2003/03/28]
14. Sadaka F, Aggu-Sher R, Krause K, et al. The effect of red blood cell transfusion on tissue oxygenation and microcirculation in severe septic patients. *Ann Intensive Care* 2011;1(1):46. doi: 10.1186/2110-5820-1-46 [published Online First: 2011/11/10]
15. Santafe Colomina M, Arikan Abello F, Sanchez Corral A, et al. Optimization of the neurosurgical patient in Intensive Care. *Med Intensiva* 2019 doi: 10.1016/j.medin.2019.02.011 [published Online First: 2019/04/16]
16. Geneesmiddelen CtBv. Alacare 8 mg pleister voor cutaan gebruik [Available from: http://db.cbg-meb.nl/ords/f?p=111:3:0:ATC:NO::P0_DOMAIN,P0_LANG,P3_RVG1:H,NL,113539 accessed 19-10-2016 2016.
17. Mik EG, Stap J, Sinaasappel M, et al. Mitochondrial PO2 measured by delayed fluorescence of endogenous protoporphyrin IX. *Nat Methods* 2006;3(11):939-45. doi: 10.1038/nmeth940 [published Online First: 2006/10/25]
18. Mik EG, van Leeuwen TG, Raat NJ, et al. Quantitative determination of localized tissue oxygen concentration in vivo by two-photon excitation phosphorescence lifetime

measurements. *J Appl Physiol (1985)* 2004;97(5):1962-9. doi: 10.1152/japplphysiol.01399.2003 [published Online First: 2004/07/13]
19. Mik EG IC, Eerbeek O, et al. Mitochondrial oxygen tension within the heart. *J Mol Cell Cardiol* 2009
20. Mik EG, Johannes T, Ince C. Monitoring of renal venous PO2 and kidney oxygen consumption in rats by a near-infrared phosphorescence lifetime technique. *Am J Physiol Renal Physiol* 2008;294(3):F676-81. doi: 10.1152/ajprenal.00569.2007 [published Online First: 2008/01/11]
21. Harms F, Stolker RJ, Mik E. Cutaneous Respirometry as Novel Technique to Monitor Mitochondrial Function: A Feasibility Study in Healthy Volunteers. *PLoS One* 2016;11(7):e0159544. doi: 10.1371/journal.pone.0159544
22. Braathen LR, Szeimies RM, Basset-Seguin N, et al. Guidelines on the use of photodynamic therapy for nonmelanoma skin cancer: an international consensus. International Society for Photodynamic Therapy in Dermatology, 2005. *J Am Acad Dermatol* 2007;56(1):125-43. doi: 10.1016/j.jaad.2006.06.006 [published Online First: 2006/12/28]
23. Reinhold U, Dirschka T, Ostendorf R, et al. A randomized, double-blind, phase III, multicentre study to evaluate the safety and efficacy of BF-200 ALA (Ameluz((R))) vs. placebo in the field-directed treatment of mild-to-moderate actinic keratosis with photodynamic therapy (PDT) when using the BF-RhodoLED((R)) lamp. *Br J Dermatol* 2016;175(4):696-705. doi: 10.1111/bjd.14498 [published Online First: 2016/02/28]
24. Kennedy JC, Pottier RH, Pross DC. Photodynamic therapy with endogenous protoporphyrin IX: basic principles and present clinical experience. *J Photochem Photobiol B* 1990;6(1-2):143-8. doi: 10.1016/1011-1344(90)85083-9 [published Online First: 1990/06/01]
25. Park SY, Kim DH, Joe HB, et al. Accuracy of cardiac output measurements during off-pump coronary artery bypass grafting: according to the vessel anastomosis sites. *Korean J Anesthesiol* 2012;62(5):423-8. doi: 10.4097/kjae.2012.62.5.423 [published Online First: 2012/06/09]
26. Critchley LA, Critchley JA. A meta-analysis of studies using bias and precision statistics to compare cardiac output measurement techniques. *J Clin Monit Comput* 1999;15(2):85-91. [published Online First: 2003/02/13]
27. Mik EG, Johannes T, Zuurbier CJ, et al. In vivo mitochondrial oxygen tension measured by a delayed fluorescence lifetime technique. *Biophys J* 2008;95(8):3977-90. doi: 10.1529/biophysj.107.126094 [published Online First: 2008/07/22]
28. Mik EG, Ince C, Eerbeek O, et al. Mitochondrial oxygen tension within the heart. *J Mol Cell Cardiol* 2009;46(6):943-51. doi: 10.1016/j.yjmcc.2009.02.002 [published Online First: 2009/02/24]
29. Harms FA, de Boon WM, Balestra GM, et al. Oxygen-dependent delayed fluorescence measured in skin after topical application of 5-aminolevulinic acid. *J Biophotonics* 2011;4(10):731-9. doi: 10.1002/jbio.201100040 [published Online First: 2011/07/20]
30. Bodmer SI, Balestra GM, Harms FA, et al. Microvascular and mitochondrial PO(2) simultaneously measured by oxygen-dependent delayed luminescence. *J Biophotonics* 2012;5(2):140-51. doi: 10.1002/jbio.201100082 [published Online First: 2011/11/25]
31. Harms FA, Bodmer SI, Raat NJ, et al. Validation of the protoporphyrin IX-triplet state lifetime technique for mitochondrial oxygen measurements in the skin. *Opt Lett* 2012;37(13):2625-7. doi: 10.1364/OL.37.002625 [published Online First: 2012/06/30]
32. van Diemen MPJ, Berends CL, Akram N, et al. Validation of a pharmacological model for mitochondrial dysfunction in healthy subjects using simvastatin: A randomized placebo-controlled proof-of-pharmacology study. *Eur J Pharmacol* 2017;815:290-97. doi: 10.1016/j.ejphar.2017.09.031
33. Gründerfonds H-T. Photonics Healthcare Berlin: High-Tech Gründerfonds Management GmbH; [Available from: https://high-tech-gruenderfonds.de/en/portfolio/photonics-healthcare-2/ accessed 21-10-2019 2019.
34. Peng Q, Warloe T, Berg K, et al. 5-Aminolevulinic acid-based photodynamic therapy. Clinical research and future challenges. *Cancer* 1997;79(12):2282-308. doi: 10.1002/(sici)1097-0142(19970615)79:12<2282::aid-cncr2>3.0.co;2-o [published Online First: 1997/06/15]
35. Gupta AK, Paquet M, Villanueva E, et al. Interventions for actinic keratoses. *Cochrane Database Syst Rev* 2012;12:CD004415. doi: 10.1002/14651858.CD004415.pub2 [published Online First: 2012/12/14]
36. Jaarboek 2018 Stichting NICE, 2018:27.

37. Ubbink R, Bettink MAW, Janse R, et al. A monitor for Cellular Oxygen METabolism (COMET): monitoring tissue oxygenation at the mitochondrial level. *J Clin Monit Comput* 2017;31(6):1143-50. doi: 10.1007/s10877-016-9966-x [published Online First: 2016/12/22]
38. Zwaag J WBM, Kox M, Mik EG. Change of mitochondrial function in vivo during human endotoxemia: preliminary data. *Ned Tijdschr Anesthesiologie* 2016
39. R. Ubbink PAWtB, E.G. Mik. Novel technique to monitor effect of transfusion on mitochondrial oxygenation. Poster ISICEM 2017, 2017.

14. Appendix 1: Results of the pilot study of the INOX ICU-2 study

Table 1 The between-subject and within-subject variability of mitoPO_2 per measurement moment. The within-subject variability is stable until 2 hours after transfusion, after which an increase is observed

Measurement moment	Between-subject variability		Within-subject variability
	Mean mitoPO_2 (mmHg)	SD mitoPO_2 (mmHg)	Mean SD of mitoPO_2 per measurement moment (mmHg)
**Before RBC**	70.5	13.0	3.0
**At the end of RBC**	68.1	11.7	3.4
**15 min after RBC**	69.5	17.7	3.5
**30 min after RBC**	68.4	18.7	5.8
**60 min after RBC**	68.1	15.8	4.6
**120 min after RBC**	70.0	20.4	3.6
**180 min after RBC**	67.5	28.2	7.3
**24 hours after RBC**	90.0	42.5	8.7

Fig. 1 Course of mitoPO_2 (mmHg) per subject during the measurements. Up to the first hour of measurements the between-subject variability seems stable, afterwards this variability of mitoPO_2 increases

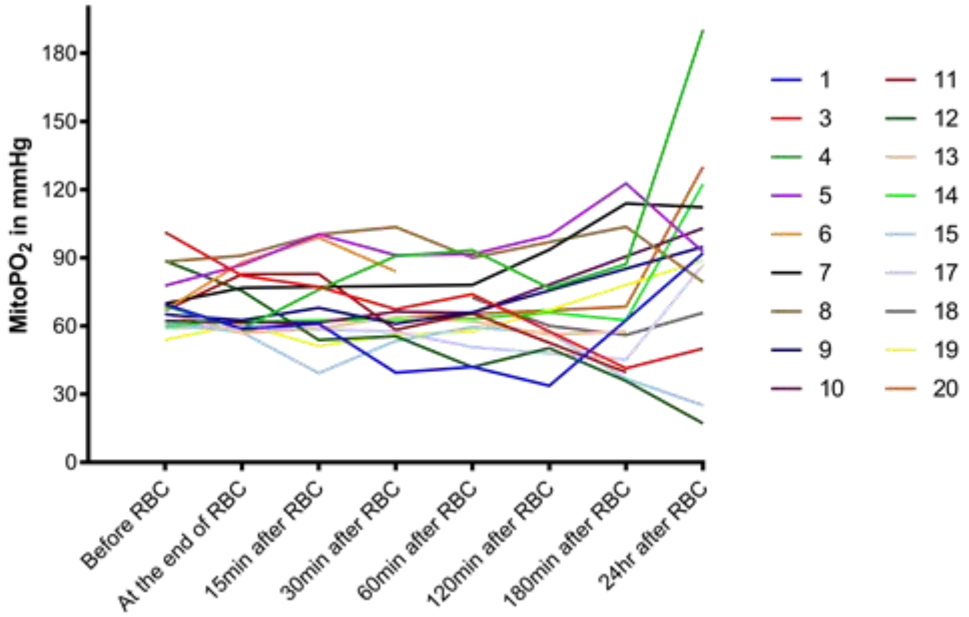

15. Appendix 2: Overview of measurements

Figure 1: Diagram showing relevant time-points in the clinical study

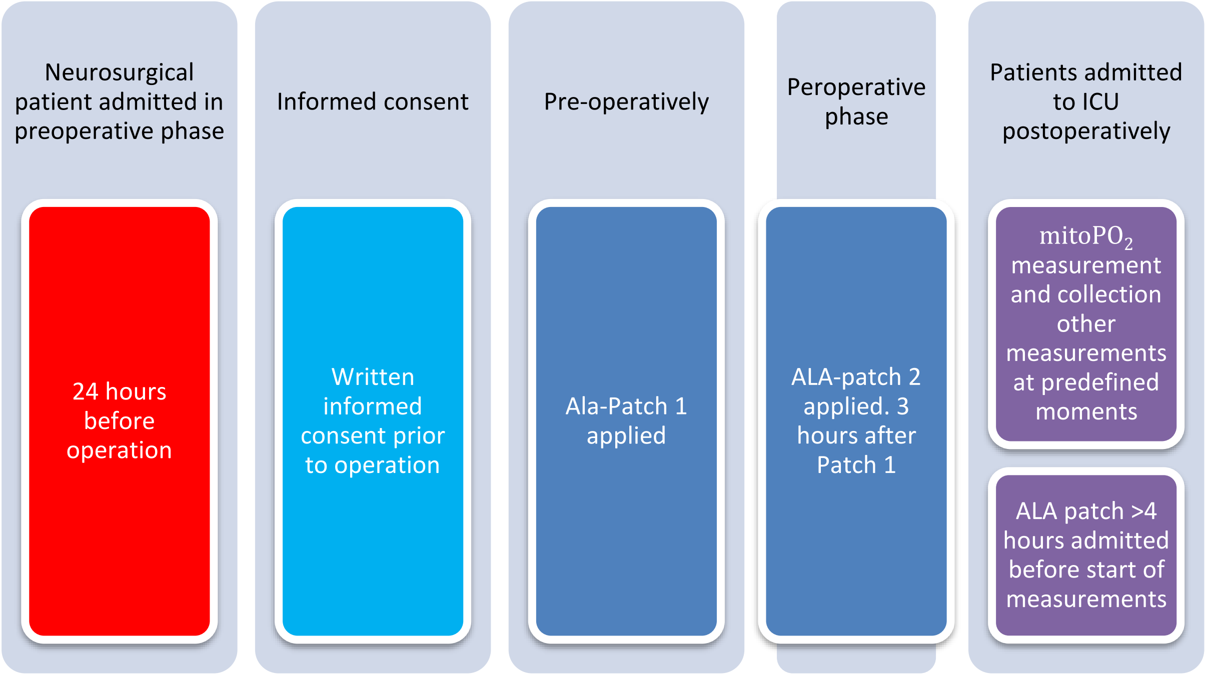

Figure 2: Diagram showing an overview of standard and extra measurements

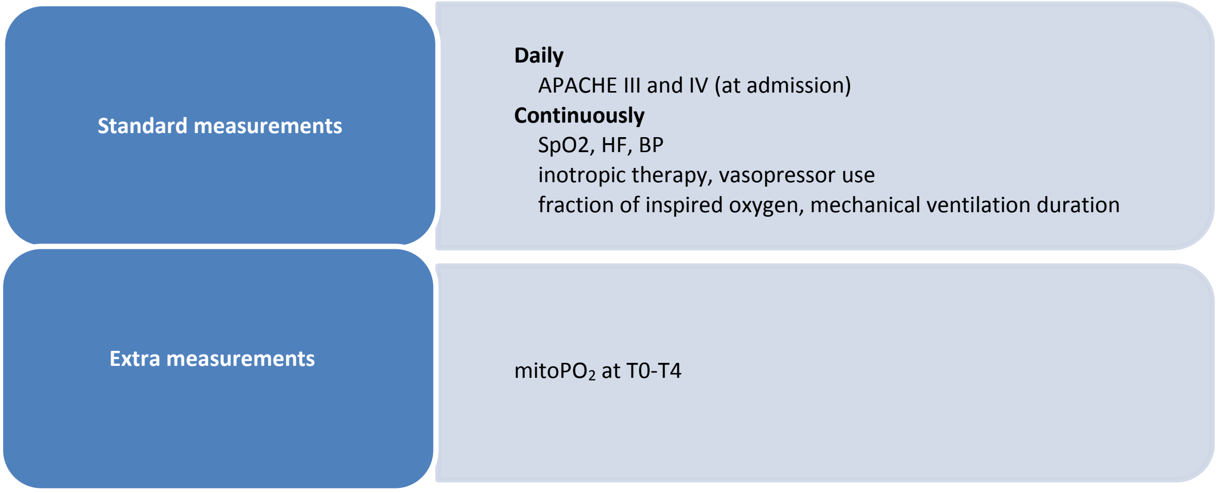

Figure 3: Flow diagram with measurement parameters for clinical study

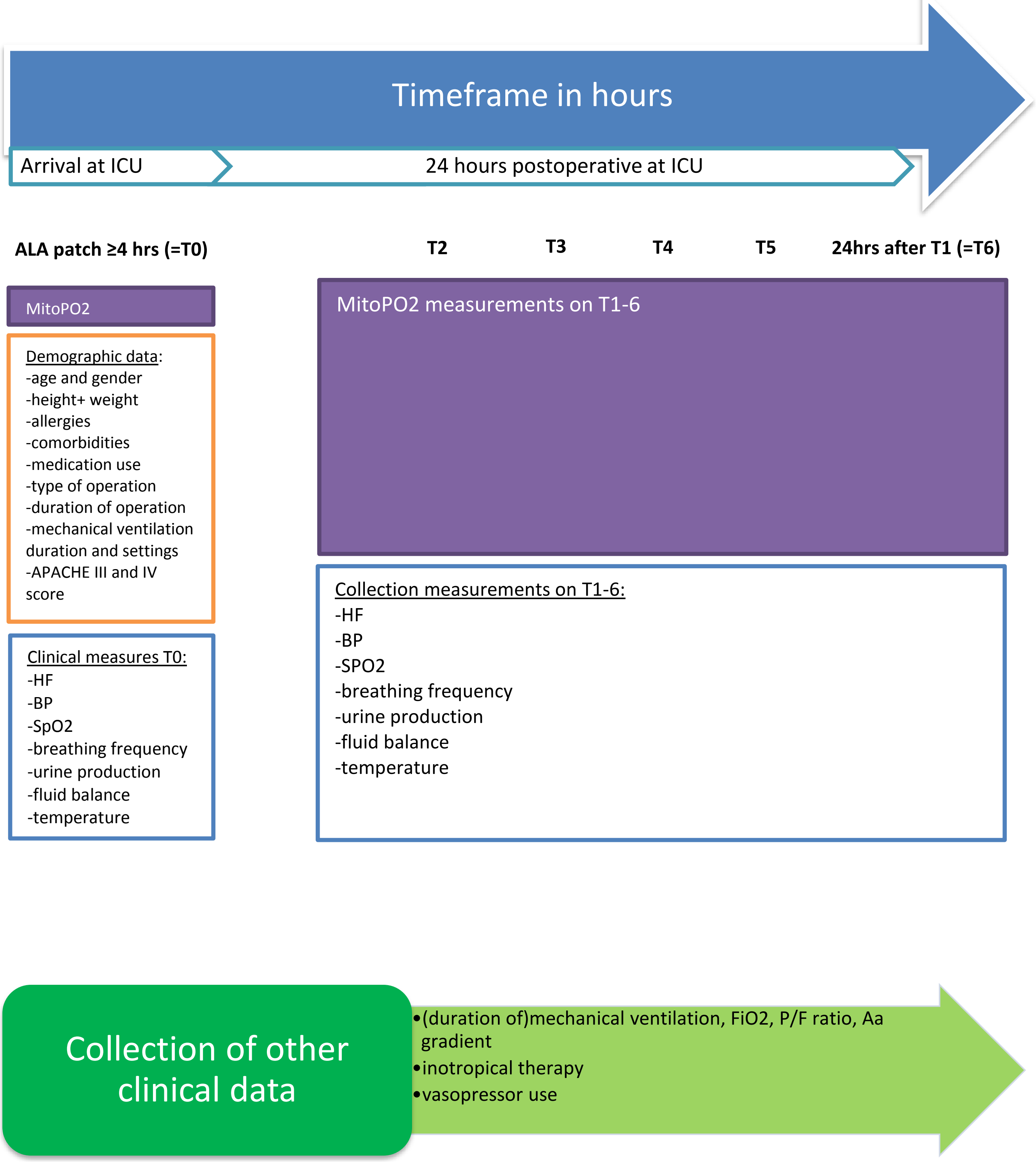

Abbreviations: *BP*=blood pressure; *FiO2*= fraction inspired oxygen; *Hb*= haemoglobin; *HF*=heart frequency; *Ht*= haematocrit; *ICU*=intensive care unit; *NYHA*= New York Heart Association class; *PaO2*= arterial oxygen tension; *SaO2*= arterial saturation;; *SpO2*=peripheral oxygen saturation.

16. Appendix 3: Monitor plan according to NFU-guidelines

	Moderate risk but intensive monitoring
Monitor frequency	1 visits per year
Patientflow[1]	Inclusion speed and percentage of patients fallen out (e.g. withdrawn, loss to follow up)
Trial Master File / Investigator File	Presence and integrity of investigation file
Informed consent	25%
In-/exclusion criteria	25% of all subjects of each centre[2]
**Source Data** Verification	25% (Based at predefined list of variables, including primary endpoint, which are in clear relation to safety and validity of the study)
**SAE[3]**	100% of the total subjects for possible missed SAE. It includes verification of procedures as well.[3]
**Investigated product**	Control of instructions given to subjects. Also, control of delivery, distribution, storage, return, expiration date and timely order of the ALA patch
**Study procedure**	Control of presence of instructions needed for study procedure. If needed, facilities and equipment will be checked as well.

1 Monitoring of patient flow regardless of the risk classification since inclusion speed going to slow can threaten completion of investigation.
2 If subjects are included wrongly, all files of the centre involved with that will be reviewed and controlled.
3 If the reporting and / or appropriate reporting of serious adverse events (SAE) is incomplete or incorrect, all files of that particular centre need to be checked, regardless of the degree of intensity of monitoring.

17. Appendix 4: Postoperative neurosurgical (NCH) admissions to LUMC Medium Care (MC) / ICU

Figure 3: Number of NCH MC admissions in 2018

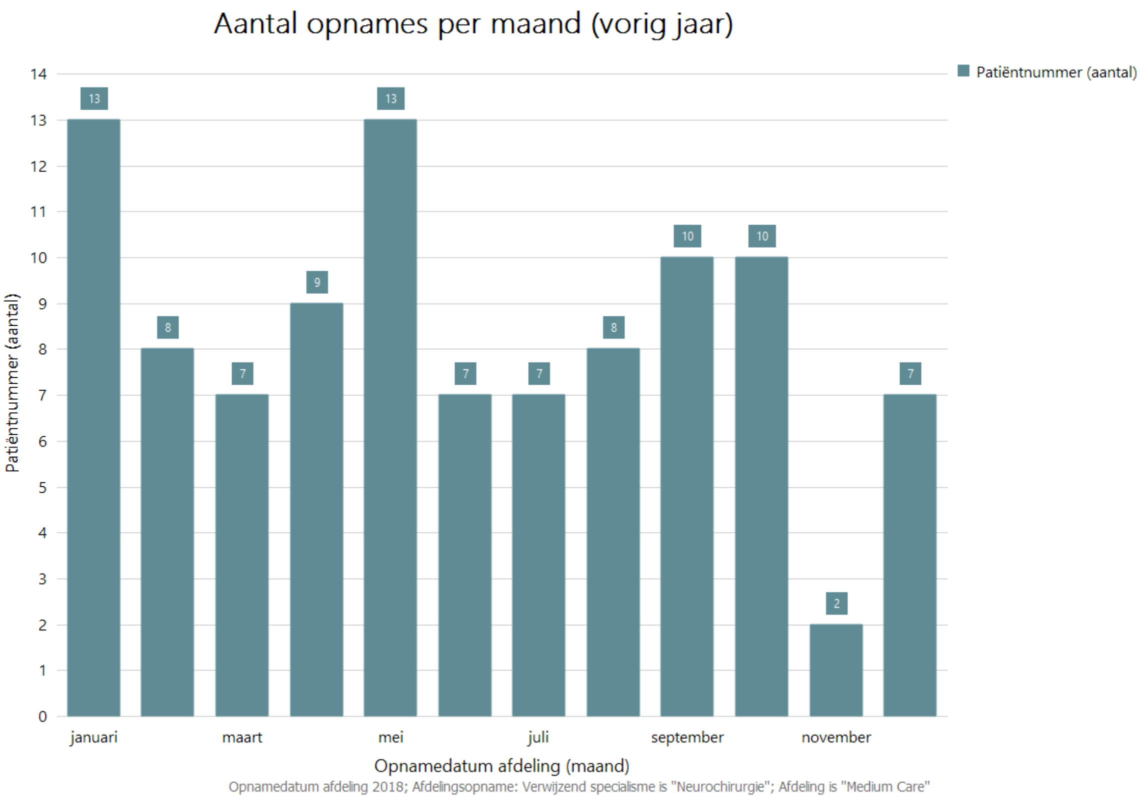

Figure 4: Number of NCH ICU admissions in 2018

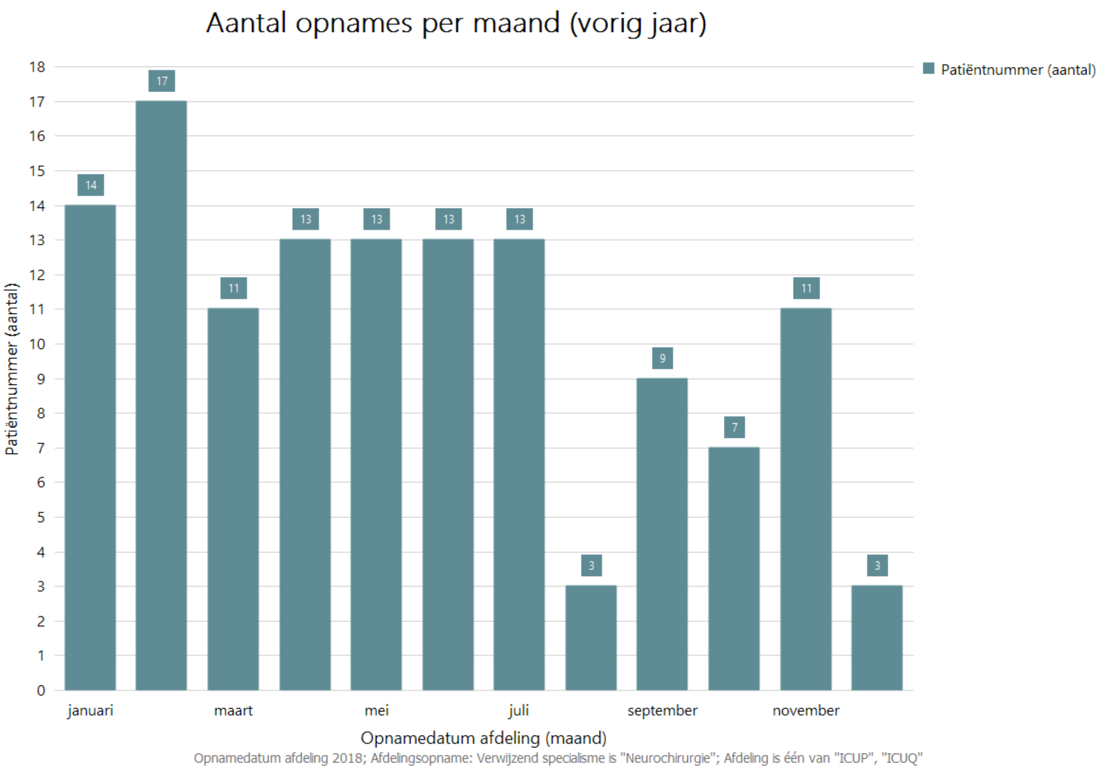

Figure 5: Number of NCH MC admissions in 2019

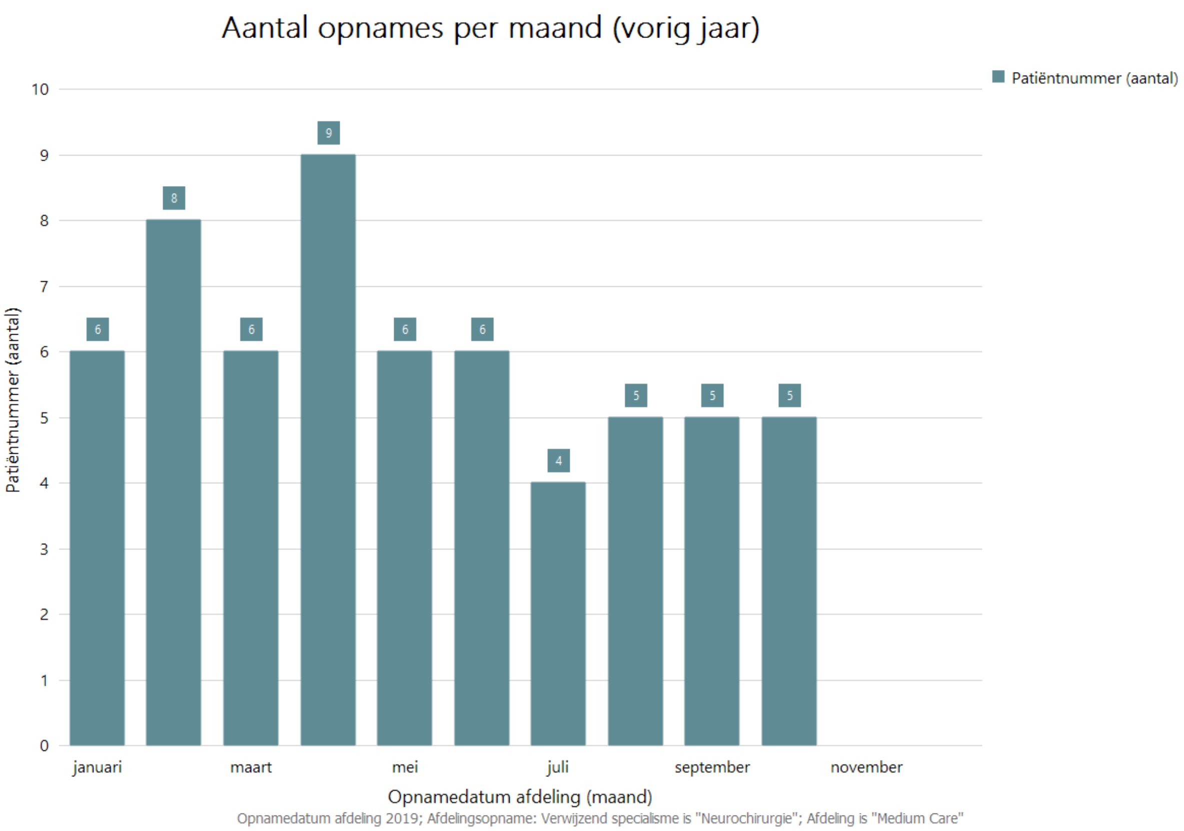

Figure 6: Number of NCH ICU admissions in 2019

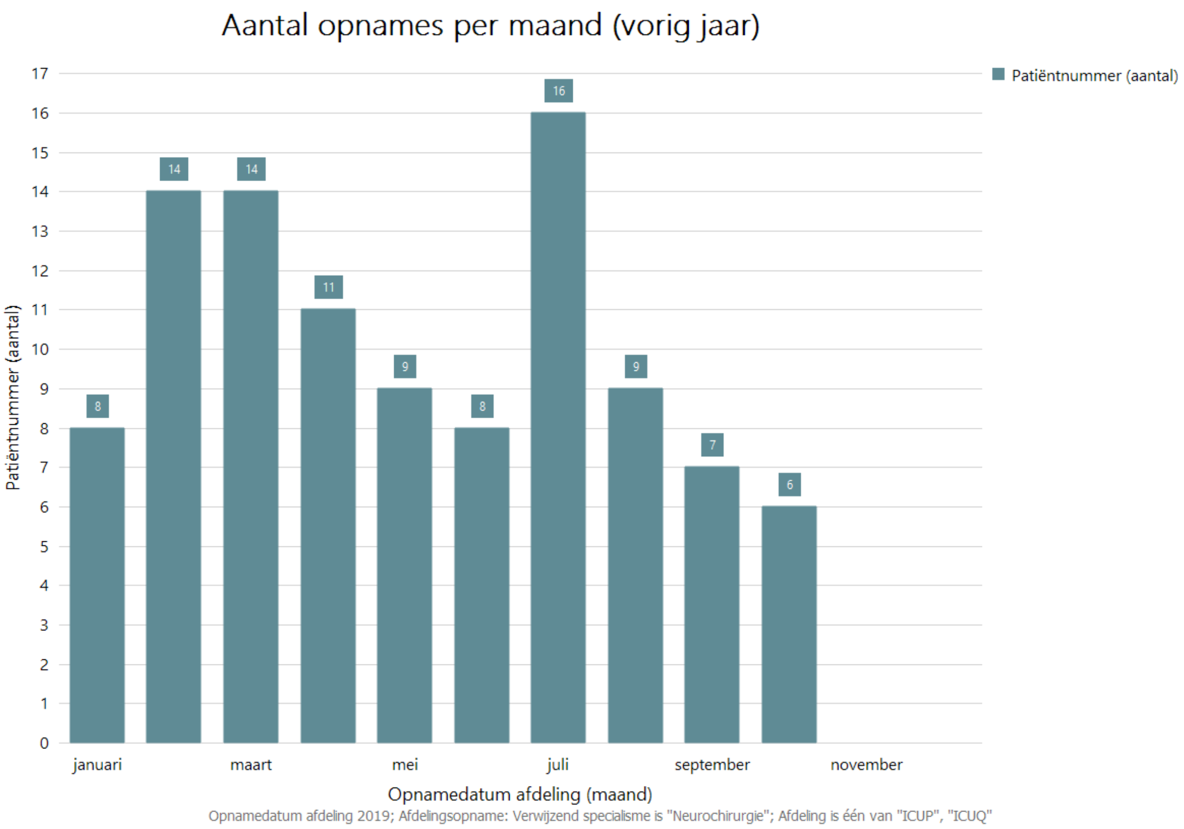

Table 1: Number of NCH MC/IC 2018 admissions with totals and averages

2018	MC	IC	Total
January	13	14	27
February	8	17	25
March	7	11	18
April	9	13	22
May	13	13	26
June	7	13	20
July	7	13	20
August	8	3	11
September	10	9	19
October	10	7	17
November	2	11	13
December	7	3	10
Total	101	127	228
Average per month	8,42	10,58	19

Table 2: Number of NCH MC/IC 2019 admissions with totals and averages

2019	MC	IC	Total
January	6	8	14
February	8	14	22
March	6	14	20
April	9	11	20
May	6	9	15
June	6	8	14
July	4	16	20
August	5	9	14
September	5	7	12
Total	55	96	151
Average per month	6,11	10,67	16,78

Table 3: Number of NCH MC/IC admissions with totals and averages for period November - February 2018/2019

Period March-June 2019	MC	IC	Total
Total	27	42	69
Average per month	6.75	10.5	17.25
